# A Collapse-Resistant Red-Billed Blue Magpie Optimizer for Employment-Policy Design: Programme Targeting and Occupational Retraining Pathways

**DOI:** 10.3390/biomimetics11100723

**Published:** 2026-10-10

**Authors:** Yang Shi, Xinyu Cai

**Affiliations:** 1 School of Labor Economics, Capital University of Economics and Business, Beijing 100070, China; 2 School of Accounting, Shandong Youth University of Political Science, Jinan 250103, China; 3 College of Business, Jiaxing University, Jiaxing 314001, China

**Keywords:** red-billed blue magpie optimizer, swarm intelligence, population diversity collapse, active labour-market policy, occupational retraining

## Abstract

Improvements to bio-inspired optimisers usually answer generic defects with generic operators. This paper instruments the red-billed blue magpie optimizer (RBMO) and measures what fails. Both operators scale their step by differences between population positions, and the attack places every candidate at the incumbent best, so the search scale shrinks with the spread the attack destroys. On five instrumented functions of the IEEE Congress on Evolutionary Computation 2017 (CEC2017) benchmark suite the population collapses to numerical identity, and on four the error stagnates over the last 28% to 58% of the run. The proposed collapse-resistant RBMO (CRBMO) breaks the loop with a dual-anchor attack, a radius control under the one-fifth success rule and an adaptive foraging allocation; it adds no chaotic map, Levy flight or opposition-based learning. On CEC2017 at 30,000 evaluations, against nine swarm algorithms, it attains the best Friedman rank at *D* = 30, 50 and 100 and 1.72 overall against 3.16 for RBMO. It outranks three published RBMO variants in every dimension, but the covariance matrix adaptation evolution strategy (CMA-ES) and the single-objective differential evolution algorithm jSO outrank it overall. At the standard CEC2017 budget its lead over RBMO is not significant; success-history-based adaptive differential evolution with linear population size reduction (L-SHADE), jSO and CMA-ES outrank it. Applied to programme targeting and retraining pathway design in employment policy, CRBMO beats RBMO in every instance, though the crested porcupine optimizer (CPO) ranks first.

## 1. Introduction

Metaheuristics drawn from collective animal behaviour have become the default instrument for optimisation problems that are large, nonlinear and non-differentiable. A cohort of them has appeared in the last four years—the dung beetle optimizer [1], the secretary bird optimisation algorithm [2] and the black-winged kite algorithm [3] among them—each introduced with reported wins over established baselines on the benchmark suites of the IEEE Congress on Evolutionary Computation (CEC). The red-billed blue magpie optimizer (RBMO) belongs to this cohort. It was introduced in 2024 with the best Friedman mean rank on the CEC2014 and CEC2017 suites at every dimension tested, against advanced variants, recently proposed algorithms and competition winners [4].

Such an algorithm quickly acquires a body of literature on improvements, and RBMO has one. That literature is the subject of this paper’s first objection. In a sample of 111 recent papers proposing new, hybrid or improved optimisers, 65% present an improved version of an established algorithm rather than a new one and only 43% so much as mention the No Free Lunch theorem [5]. The theorem matters here: because elevated performance on one class of problems is exactly offset by performance on another [6], an optimiser can be justified only against a stated class of problems, which is why the target class in this paper—two employment-policy design problems—is fixed before any claim of superiority is made. A second criticism concerns novelty: the field’s own critics have argued that the novelty in many proposals lies in the metaphor rather than in the search behaviour, and that algorithms drawn from natural, artificial and social phenomena keep being introduced without a clear motivation in terms of the operators they actually apply [7,8].

The published improvements to RBMO illustrate the pattern. They diagnose the algorithm in generic terms and answer with general-purpose operators added to the original update: one attributes premature convergence and degradation on high-dimensional constrained problems to an over-reliance on population mean vectors and adds a chaotic map, a dynamic balance factor and a Levy perturbation [9]; a second names declining diversity in the middle and later stages and adds a chaotic map, a Levy perturbation, a Cauchy–Gauss mutation and a stochastic differential mutation [10]; a third names trapping in local optima and adds a good-nodes-set initialisation and lens-imaging opposition-based learning [11]. Chaotic maps, Levy flights, opposition-based learning and Cauchy mutation can be attached to any population-based optimiser without knowing anything about RBMO. None of them follows from a measurement of what RBMO does.

This paper takes the opposite route. RBMO’s operators were instrumented and run, and the state of the population was recorded at every iteration. The measurement identifies a specific mechanism rather than a symptom: both operators scale their step by the displacement of an individual from the centroid of its foraging party, and the attacking operator places every candidate at the incumbent best. The scale of the search is therefore proportional to the population spread that the attack is destroying, which is a positive feedback loop. Measured on five CEC2017 functions at *D* = 30, the spread falls below the floor of 10^−12^ at a median of 56% to 75% of the evaluation budget on four of them and at 95% on the remaining one. On those four the error stops changing in its fourth significant figure over the last 28% to 58% of the run, and over the last quarter of the budget 91% to 100% of the attacking candidates return exactly their parent’s value; that part of the run is spent re-evaluating points that are numerically indistinguishable from their parents. The proposed algorithm, the collapse-resistant red-billed blue magpie optimizer (CRBMO), repairs that loop and nothing else: it adds no chaotic map, no Levy flight and no opposition-based learning.

The second half of this paper asks what such an algorithm is for. Labour markets are being reshaped by measurable shocks: one more industrial robot per thousand workers lowers a local employment-to-population ratio by 0.2 percentage points and wages by 0.42% [12], and higher local adoption of artificial intelligence reduces the change in that ratio across commuting zones [13]. The public response to such shocks is active labour-market policy, whose effects have been measured with meta-analytic precision across 207 studies and 857 estimates: close to zero shortly after a programme ends, larger at medium and long horizons, and sharply different by programme type and by jobseeker group [14]. What that literature measures, however, is what programmes did, not what a planner should fund. The distinction matters: in an adaptive targeted field experiment the interventions themselves barely moved employment while targeting them did [15], which locates the leverage in allocation rather than in treatment. Allocation is an optimisation problem, but the evaluation literature has rarely treated it as one to be solved with an optimiser. The nearest precedent designs a tax schedule by two-level deep multi-agent reinforcement learning, raising utilitarian social welfare by 8% over a Saez baseline [16]; that work learns a policy, whereas the problem here is a search over a constrained, budget-limited design space of fixed dimension, the kind of problem a metaheuristic addresses.

The contributions of this paper are as follows.

(1) A measured diagnosis of RBMO. Instrumenting both operators identifies a feedback loop between the step scale and the population spread, and shows the cost of that loop: a stagnant stretch occupying the last 28% to 58% of the run on four of the five instrumented functions. The diagnosis is falsifiable and is tested against a ten-fold evaluation budget.
(2) Three targeted repairs, and no new operator. A dual-anchor attack, a collapse-triggered radius control under the one-fifth success rule, and an adaptive foraging allocation. A complete factorial ablation attributes the gain to the first two and shows that the third pays only at high dimension.
(3) An evaluation designed to be falsifiable. The full CEC2017 suite in four dimensions against nine swarm algorithms over thirty runs, extended to three evolutionary algorithms, three published RBMO variants and the standard CEC2017 budget, with parameter sensitivity, factorial ablation and Friedman, Nemenyi, Holm and Wilcoxon analyses—and an explicit statement of where the repair does not pay.
(4) Two employment-policy design problems, calibrated from published quasi-experimental estimates, on which the algorithm is tested as an instrument rather than as a benchmark entry.

The remainder of the paper is organised as follows. Section 2 states RBMO and reviews its published improvements. Section 3 reports the measurement and the diagnosis. Section 4 develops CRBMO. Section 5 gives the CEC2017 evaluation and Section 6 the two applications. Section 7 discusses what the results do and do not support, and Section 8 concludes.

## 2. The Red-Billed Blue Magpie Optimizer

This section states RBMO in the form used throughout the paper and reviews the improvements published since. The statement matters because the diagnosis of Section 3 turns on two specific features of the update equations, and the review of Section 2.3 is the evidence that the present paper is answering a question the literature has left open rather than one it has already answered.

### 2.1. Biological Inspiration

The red-billed blue magpie (*Urocissa erythroryncha*) forages in parties. A party of two to five birds works a patch closely, while a cluster of ten or more sweeps a wider area; a bird watches where its companions are finding food and drifts towards them, and once prey has been located the party converges on it together. What a bird brings back is stored only if it is better than what the bird already holds. Three features of that account matter for an optimiser. The first is that the pull on a bird is towards a group of companions rather than towards one leader, which is a differential rather than a point attraction. The second is that the size of the group sets how wide the bird’s step is. The third is that storage never accepts a worse position, so the colony never loses ground.

**Figure 1:**
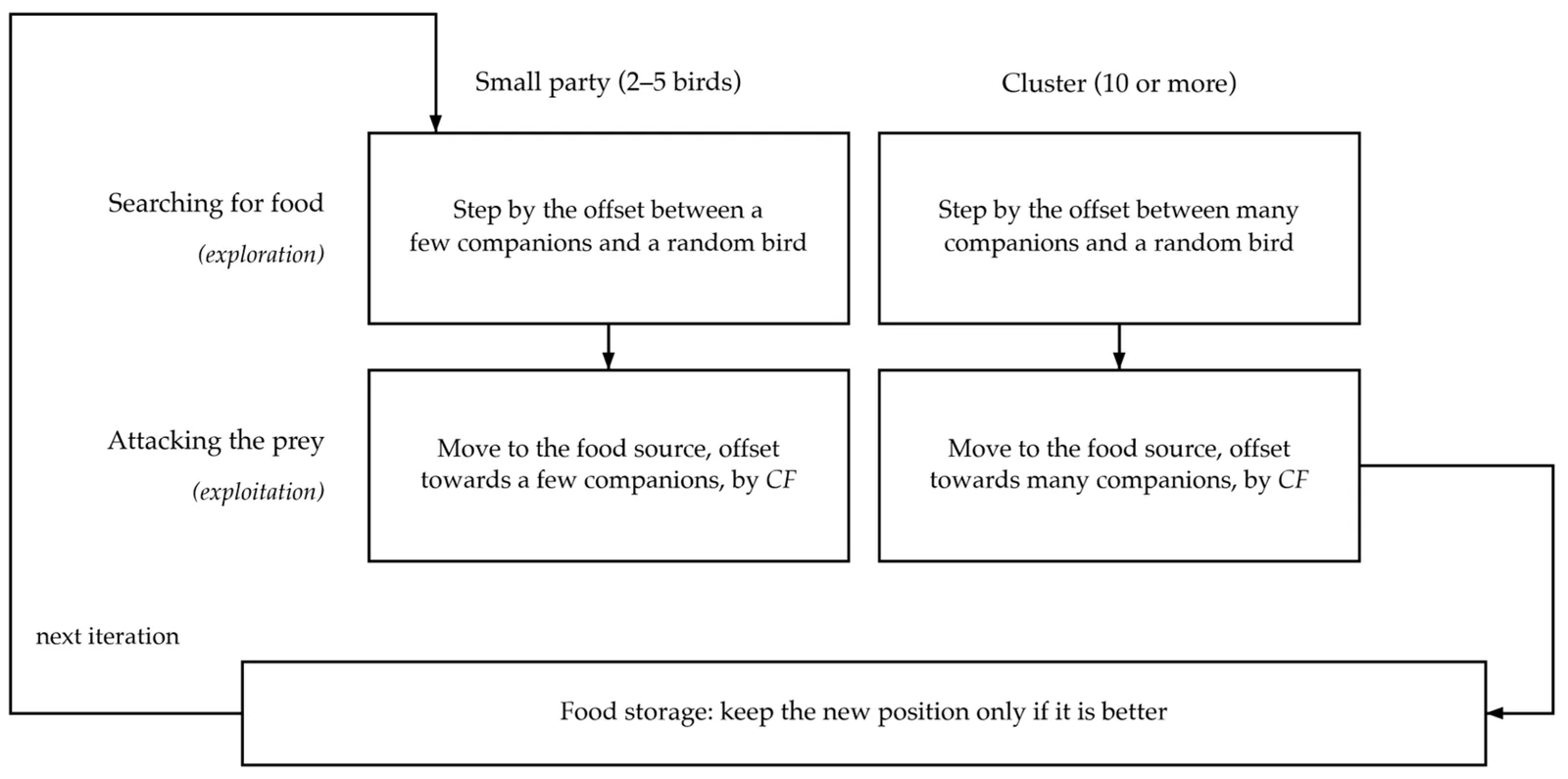
Correspondence between the foraging behaviour of the red-billed blue magpie and the search operators of RBMO.

The original paper turns this into four operators: searching for food in a small group, searching in a large group, attacking the prey with a small group and attacking with a large group, followed by the food-storage rule [4]. Figure 1 maps the correspondence. The first pair supplies exploration and the second exploitation, and the transition between them is carried by a single contraction factor rather than by an explicit schedule over the population. The description below follows the authors’ own released implementation, which resolves the places where the published equations leave room for interpretation.

### 2.2. Mathematical Model

Consider the minimisation of a *D*-dimensional objective *f*(**x**) over a box [**lb**,**ub**]. RBMO maintains *n* candidate solutions, initialised uniformly at random as in Equation (1). The source counts iterations; because every iteration costs the same number of evaluations, *t* and *T* are written here as consumed and total function evaluations, which is the same ratio. **x**_food_ is the best individual found so far.

**x***_i_* = **lb** + **r**⊙(**ub** − **lb**), *i* = 1,2,…,*n* (1)

where **r** is a vector of independent uniform variates on [0,1] and ⊙ is the elementwise product.

Searching for food. Each bird draws a foraging party of *g* companions uniformly at random from the population and steps by the displacement between that party’s centroid and one further individual **x***_r_*, also drawn uniformly at random, scaled by a single uniform variate, as in Equation (2). The party is small, *g*∼𝒰{2,…,5}, or a cluster, *g*∼𝒰{10,…,*n*}, with probability one half each.

(2) xi new=xi+1g∑q=1gxq−xrρ,ρ∼U(0,1)

Two properties of this rule are used in Section 3. The step does not reference the moving bird’s own position at all, and it is a difference between two positions drawn from the population, so its length is proportional to how widely that population is spread.

Attacking the prey. The party converges on the incumbent best, contracted by the convergence factor *CF* of Equation (3), as in Equation (4).

(3) $$\mathrm{CF}={\left(1-\frac{t}{T}\right)}^{2t/T}$$

(4) xi new=xfood+CF1g∑q=1gxq−xi⊙η

where *η* is a vector of independent standard normal variates, one per coordinate. *CF* falls from one to zero over the run, so the attacking operator places candidates ever closer to **x**_food_. Note that the candidate is anchored on the food source and not on the attacker: the attacking bird’s own position enters only through the displacement term, and that displacement is now measured from the bird itself rather than from another draw.

Food storage. Both stages are followed by the greedy replacement of Equation (5), so an individual never moves to a worse position. Equation (5) is written with a strict inequality, as in the published description and in CRBMO; the released code also keeps a candidate whose value equals the current one. The difference is immaterial on the continuous functions of Section 5, and Section 6.3 shows that it is immaterial on the plateaus of the applications as well.

(5) $$x_{i}\leftarrow \left\{\begin{array}{c} x_{i}^{\mathrm{new}},\;\;\;f(x_{i}^{\mathrm{new}})<f(x_{i})\\ x_{i},\;\;\;\mathrm{otherwise} \end{array}\right.$$

One iteration therefore costs 2*n* function evaluations, *n* for each stage. Two properties of this design are used in Section 3: both steps are scaled by a difference between positions drawn from the population, and every attacking candidate is placed at the food source.

### 2.3. Published Improvements and What They Diagnose

Table 1 records five representative papers, published in 2025 and 2026, that propose improved versions of RBMO, with the defect each states and the operators each adds. The defects are essentially the same throughout—premature convergence, loss of population diversity, and an imbalance between exploration and exploitation—usually stated together in a single sentence of the introduction and then left without further analysis.

Two things stand out. The first is that the remedies are interchangeable. Chaotic initialisation, opposition-based learning, Levy flights and Cauchy or differential mutation are general-purpose devices; none of them is derived from anything RBMO does, and any of them could be attached to any population-based optimiser. The second is that two papers in Table 1 point at a mechanism rather than a symptom. One attributes RBMO’s premature convergence and its degradation on high-dimensional constrained problems to an over-reliance on population mean vectors [9], and the other reports that RBMO’s attraction is unopposed and adds an explicit repulsion term to counter it [17]. Both are pointing at the same part of the algorithm, but neither measures it; Section 3 does.

## 3. Where RBMO Loses, Measured

The improvement literature reviewed in Section 2.3 is nearly unanimous about what is wrong with RBMO and nearly silent about why. All five variant papers of Table 1 name a defect; premature convergence, declining population diversity and entrapment in local optima account for almost all of them, and the remedies are correspondingly generic—a chaotic map on the initial population, a Levy flight added to one operator, an opposition-based learning step, a Cauchy mutation. Only two of them locate the defect inside the update rules, and the first names the right thing: an over-reliance on population mean vectors [9]. This section takes that reading seriously and measures it.

Three measurements are reported. The first is a rank table already in hand: how RBMO stands against the other leading algorithms, broken down by function group. The second asks what a ten-fold budget buys it. The third instruments both of its operators and follows the state of the population through a run. Together they identify a single feedback loop, and the loop is what Section 4 repairs.

### 3.1. What the Rank Tables Already Say

Every rank reported in this paper is produced the same way: algorithms are ranked on each function by mean error, the ranks are aggregated by the Friedman test, and a significant result is followed by the post hoc analysis that current guidelines prescribe for comparing swarm and evolutionary algorithms [19,20]. Per-function differences are reported separately, and are descriptive rather than a second inferential layer: the family-wise error rate is controlled by the post hoc procedure, not by the per-function tests. Section 5.1 gives the details; what is needed here is only that the numbers below and the numbers in Section 5.4 are commensurable.

Table 2 ranks RBMO against the three strongest of the other comparison algorithms of Section 5.4 within each CEC2017 function group. RBMO is the first of the four on the unimodal functions (1.50), first on the simple multimodal (1.93), joint first on the hybrid (2.08) and third on the composition functions (2.60), a spread of 1.10 rank units on a four-point scale. The unimodal functions reward precise convergence within a single basin, and this is where RBMO does best; the composition functions reward locating and moving between basins, and this is where it does worst. The profile is that of a search that converges well but loses the ability to move once it has converged, which is what the loop of Section 3.3 predicts.

The second reading is a control. If the stagnant stretch were simply slowness, a larger budget would buy it away; if the search were permanently stalled, more evaluations would buy nothing. Neither is what happens. At the standard CEC2017 budget of 10,000*D* evaluations (Section 5.7), RBMO’s mean error at *D* = 30 is lower by more than a tenth than at 30,000 evaluations on 22 of the 29 functions, so the search is not permanently stalled. What a larger budget does not reduce, except on F1, is the share of the run it wastes. Instrumented at both budgets on the same five functions (Section 3.2), the stretch over which RBMO’s error no longer changes in its fourth significant figure occupies 29% of a 30,000-evaluation run and 31% of a 300,000-evaluation one on F5, 6% and 15% on F15, 28% and 29% on F21 and 58% and 76% on F28; only on F1 does it shrink (51% to 13%), because the ten-fold budget lets RBMO converge on this unimodal function before its population collapses. On four of the five functions, then, the loss is a share of whatever budget the algorithm is given rather than a fixed quantity that a larger budget amortises, which is what a step scale tied to the population spread predicts. Section 5.7 reports the full-budget comparison; the instrumentation of Section 3.2 shows the mechanism.

### 3.2. Instrumenting the Operators

Rank tables do not show the “why”. Both RBMO operators were therefore instrumented directly. At every iteration of every run the following were recorded: the normalised population spread of Equation (6), the mean over coordinates of the population standard deviation in that coordinate, rescaled by the box width; the fraction of candidates the food-storage rule of Equation (5) accepts, separately for the searching and the attacking stage; the mean Euclidean length of the step each stage proposes; and the convergence factor. The measurement covers five CEC2017 functions, one from each group and two from the composition group, at *D* = 30 with 30 independent runs each, at the population size and budget used throughout this study and at ten times that budget. For every run the point at which the best error stops changing in its fourth significant figure (a relative change below 10^−4^ up to the end of the run) and the point at which the spread first falls below 10^−12^, the floor used throughout this section, are recorded.

(6) $$\sigma (X^{t})=\frac{1}{D}\sum_{j=1}^{D}\frac{{\mathrm{sd}}_{i}\left(x_{\mathrm{ij}}^{t}\right)}{ub_{j}-lb_{j}}$$

A value of 10^−3^ means the population occupies a thousandth of the box in each coordinate on average; a value at the level of the double-precision epsilon means the individuals are numerically indistinguishable.

**Figure 2:**
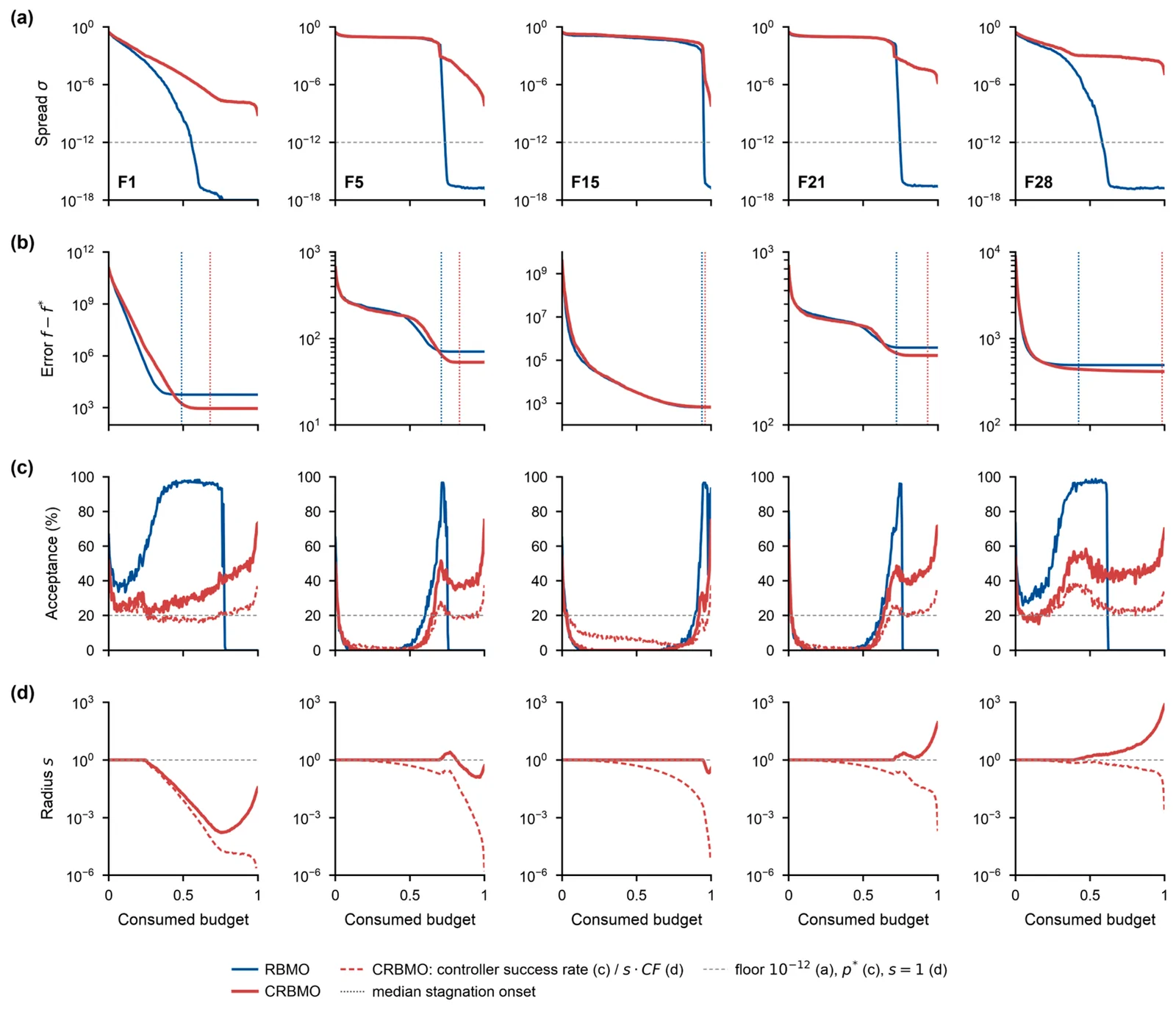
The search state of RBMO and CRBMO against the consumed budget (*D* = 30): (**a**) population spread; (**b**) error; (**c**) acceptance rate; (**d**) radius *s*. In (**b**), the blue dotted vertical lines mark the median onset of RBMO stagnation and the red vertical lines the corresponding onset for CRBMO.

Figure 2 shows the result for each function separately, as the median over 30 runs (the geometric mean for the error) and on a logarithmic scale wherever a quantity spans orders of magnitude, so that no function’s error scale dominates the others. The normalised population spread of RBMO falls below the floor of 10^−12^ in every run on every function, but at different points of the budget: at a median of 56% on F1, 58% on F28, 74% on F5, 75% on F21 and 95% on F15. The best error stops changing in its fourth significant figure before that, in every run and by a median of 1% to 16% of the budget, and on F1, F5, F21 and F28 the stagnant stretch occupies the last 28% to 58% of the run, against 6% on F15. Once the spread is at the floor the attacking stage re-evaluates copies of its parents: over the last quarter of the budget, 91% to 100% of its candidates on F1, F5, F21 and F28 return exactly their parent’s value, against 3% on F15.

The same instrumentation applied to CRBMO, plotted alongside, shows what breaking the loop does. Its spread does not reach the floor in any of the 150 runs; at the end of the run its median lies between 7.9 × 10^−10^ and 1.4 × 10^−5^. Its error keeps changing until a median of 68% to 98% of the budget, and its median final error is lower than RBMO’s on F1, F5, F21 and F28 (F1: 1147 against 5885; F5: 56.7 against 66.1; F21: 250 against 283; F28: 411 against 462) and slightly higher on F15 (640 against 607). Row (d) shows the controlled radius *s* of Section 4.2: it follows a trajectory of its own rather than the population’s, and Section 4.2 describes what it does.

### 3.3. The Loop

The three measurements compose into one mechanism. The attacking operator of Equation (4) places every accepted candidate at the food source, so acceptance moves an individual towards the incumbent and the population contracts. Both operators take a step whose length is a difference between positions drawn from that same population: the searching stage steps by a party centroid minus a randomly drawn individual, the attacking stage by a party centroid minus the individual itself. A contraction therefore shortens the next step, a shorter step contracts further, and the loop closes on itself. The convergence factor *CF* of Equation (3) accelerates the process but is not its cause: *CF* reaches zero only at the end of the budget, whereas the spread reaches the floor of 10^−12^ by three quarters of the budget on four of the five instrumented functions.

The loop also explains the dimensional pattern of the rank tables. A larger box needs more evaluations to cover, so at higher dimension the population is still far from any optimum when the contraction begins, and the evaluations lost to the stagnant stretch are lost at a worse point. This is a prediction, not a restatement: it says that a repair aimed at the loop should buy more at high dimension than at low. Section 5.4 finds this at the 30,000-evaluation budget, and Section 5.7 finds it only in part at the standard CEC2017 budget.

Three design choices carry the loop, and each can be changed without touching the foraging metaphor. The attack is anchored on the food source rather than on the attacker. The step scale is a statistic of the population rather than a controlled quantity, in both stages. And the evaluation budget is split evenly between the two stages however unproductive one of them has become. Section 4 repairs them one at a time.

## 4. The Collapse-Resistant Red-Billed Blue Magpie Optimizer

Section 3 located RBMO’s failure in a single feedback loop: every step is scaled by a difference between positions drawn from the population, and every attacking candidate is placed at the food source, so the operators drive the population onto one point and then inherit the vanishing scale of the population they have collapsed. CRBMO breaks that loop three times. Nothing is added to the foraging metaphor. None of the three devices the RBMO improvement literature reaches for appears anywhere in CRBMO: no chaotic map is substituted for a uniform draw and no Levy-distributed step is grafted onto an operator, as in [9,10], and no opposition-based refresh is applied to the population, as in [11]. The group sizes, the convergence factor and the food-storage rule are inherited unchanged. What changes is where a candidate is anchored, what sets the scale of a step, and how the evaluations are divided between the two stages.

### 4.1. Dual-Anchor Attack

The attacking operator of Equation (4) places every candidate at **x**_food_. Accepting such a candidate moves the individual to the neighbourhood of the incumbent, so a run of successful attacks empties the population into one basin, and the measurement of Section 3 shows this is what drives the spread to machine precision. The repair is to let half the party attack from where it stands, as in Equation (7).

(7) xi new=ai+CF1g∑q=1gxq−xi⊙η,   ai=xfood,   ri<1/2xi,   otherwise

with *r_i_*∼𝒰(0,1) drawn per individual per iteration. The food-anchored half keeps RBMO’s convergence mechanism intact; the self-anchored half keeps a population for the searching stage to work with. The change costs nothing: the same *n* candidates are generated and evaluated. Replacing a single attractor by a mixture of an incumbent and the individual itself is the same device that the current-to-*p*best mutation of adaptive differential evolution uses to keep a population from emptying into the incumbent [21], reached here from a measurement of RBMO rather than by analogy.

### 4.2. Collapse-Triggered Radius Control

Anchoring alone does not restore the scale of the step, because both operators still take it from a population difference. Write $d_{i}^{0}$ for that difference—the party centroid minus **x***_r_* in the searching stage of Equation (2), the party centroid minus **x***_i_* in the attacking stage of Equation (4). CRBMO multiplies it by a scalar radius *s* and adds a small isotropic term so that the step does not vanish merely because the population has contracted onto a point, as in Equation (8); the radius itself can still be driven to its lower bound (Section 5.7).

(8) $$d_{i}=sd_{i}^{0}+s\varepsilon (\mathrm{ub}-\mathrm{lb})⊙\xi ,\;\;\;\xi ∼N(0,I)$$

with *ε* = 10^−3^ fixed, and the radius *s* is kept within [10^−14^, 10^6^]. While the normalised spread of Equation (6) is above the collapse threshold *τ*, which is set below, the control is dormant: *s* is neither applied nor updated and no isotropic term is added, so the displacement is RBMO’s own, and Equation (8) replaces it only while the population is contracted below *τ*. The radius starts at *s* = 1; if the spread rises above *τ* again after the control has acted, *s* keeps its last value and the control resumes from it. Every candidate is then clipped to the box, the bound handling that every algorithm in this paper shares except the three evolutionary algorithms of Section 5.8 (Section 5.1). In the attacking stage the displacement of Equation (8) is multiplied by *CF*, so the isotropic term enters the attack with a per-coordinate standard deviation of *CF s ε* (*ub_j_* − *lb_j_*), not *s ε* (*ub_j_* − *lb_j_*).

The radius is controlled by one of the oldest step-size rules of evolution strategies. Let *α_t_* be the success rate of the previous iteration, *α_t_* = (*A*_s_ + *A*_a_)/(2*n*), where *A*_s_ and *A*_a_ are the numbers of searching and attacking candidates that strictly improved on their parents; an individual that does not scout (Section 4.3) counts as a failure of the searching stage. The one-fifth success rule of Equation (9) expands the radius while the search is succeeding and contracts it when it is not [22].

(9) $$s\leftarrow \left\{\begin{array}{c} s/c,\;\;\;\alpha_{t}>p^{*}\\ s\;c,\;\;\;\mathrm{otherwise} \end{array}\right.$$

with *c* = 0.94 and p^*^ = 0.20. The control is not active from the first iteration. RBMO’s own scaling is adequate while the party is genuinely spread, and switching the rule on immediately costs performance at low dimension, where the collapse does not bite within the budget. The rule is therefore dormant until the normalised population spread of Equation (6), the quantity Section 3.2 instrumented, falls below a threshold *τ*. The radius control is a rescue, not a replacement: it does nothing until the loop that Section 3 measures has actually started to close.

Figure 2d shows the trajectories this produces. The radius is not monotone, and its median over 30 runs stays well inside those bounds, ranging from 1.7 × 10^−4^ (F1) to 728 (F28). On F1 it falls to that minimum at 75% of the run and recovers to 0.035 by its end; on F28 it rises from about 40% of the run to 728 at its end and on F21 it rises from about 70% of the run to 86 at its end. On F5 it rises to 2.6 when the control first acts, at about 70% to 77% of the run, falls to 0.12 and turns upward again only in the last few per cent, and on F15, where the control is dormant until about 95% of the run, it falls to 0.21 and likewise turns upward only at the very end. On F1, F21 and F28 the radius is rising over most of the last quarter, because *α_t_* exceeds p^*^ at most of its iterations (70% to 80% of them in the median run). On every function *α_t_* ends at 0.35 to 0.38, where *CF* approaches zero. The product *s* ⋅ *CF*, which sets the scale of the attacking step, nevertheless falls on every function from the middle of the run to its end, by a factor of 431 on F28, 2.5 × 10^3^ on F21 and of more than 10^5^ on F1, F5 and F15. Over the last quarter of the run the attacking stage accepts 41% to 48% of its candidates on the four functions where the control is live, more than p^*^ = 0.20, because *α_t_* averages the two stages with equal weight and the searching stage adds almost nothing to it: only about an eighth of the population scouts, every individual that does not counts as a failure, and the scouts’ candidates are accepted in 2.2% to 3.5% of cases. *α_t_* itself averages 0.21 to 0.24 over that stretch on the functions where the control is live. Once the population has contracted, the isotropic term carries most of the attacking step: over the last quarter of the run its norm exceeds that of the rescaled population difference by a factor of 2.3 (F28) to 6.1 × 10^4^ (F1). The late attack is then a zero-mean perturbation of the anchor of Equation (7), with a per-coordinate standard deviation set jointly by the convergence factor and the success rule and a mean step length of 4.1 × 10^−7^ to 8.6 × 10^−3^ at the end of the run in a box of width 200: a local search around the incumbents rather than a search of the box. The searching stage, whose displacement is not multiplied by *CF*, is where the isotropic term does produce large steps: as the radius rises on F21 and F28, the mean searching step over the last quarter of the run reaches 4.2 and 31, and 3.3% and 3.5% of these candidates are accepted. The searching stage takes about a tenth of the evaluations in that stretch, a share the foraging allocation reduces to zero by the end of the run. Section 5.2 measures how much of CRBMO’s performance depends on *ε*, including *ε* = 0, and Section 5.7 reports where the rule fails.

A scalar radius is a deliberately small piece of machinery. The alternative is to adapt the whole mutation distribution from an evolution path, as covariance matrix adaptation does [23]. That would replace RBMO’s operators rather than rescale them, it would cost *O*(*D*^2^) memory per individual and it would make the attribution of any gain ambiguous. One scalar under one classical control law is the smallest change that breaks the dependence Section 3 measured, and the ablation of Section 5.3 can therefore say what it bought.

### 4.3. Adaptive Foraging Allocation

RBMO spends exactly half of every iteration’s evaluations on the searching stage, from the first iteration to the last. That stage is undirected, and late in a run its acceptance rate is the lower of the two. CRBMO lets only a fraction of the party scout, declining linearly with the consumed budget as in Equation (10).

(10) $$\phi (t)=1-(1-\phi_{\min})\frac{t}{T}$$

Individual *i* scouts if *r_i_* < *ϕ*(*t*), so an iteration costs (*ϕ*(*t*) + 1)*n* evaluations rather than 2*n*, and the evaluations RBMO spends late on an undirected walk are spent on the attack instead. Averaged over the ablation of Section 5.3 this changes little, and its effect depends on dimension: it costs rank at low dimension and pays at *D* = 100, where the collapse costs most. A success-history scheme would learn the split from the moves that recently produced improvement [21]; a deterministic schedule is used instead because the measurement of Section 3.2 already fixes the sign and the timing of the reallocation, and a learned split would add a second adaptive loop whose contribution the ablation could not separate from the radius.

### 4.4. Implementation

**Figure 3:**
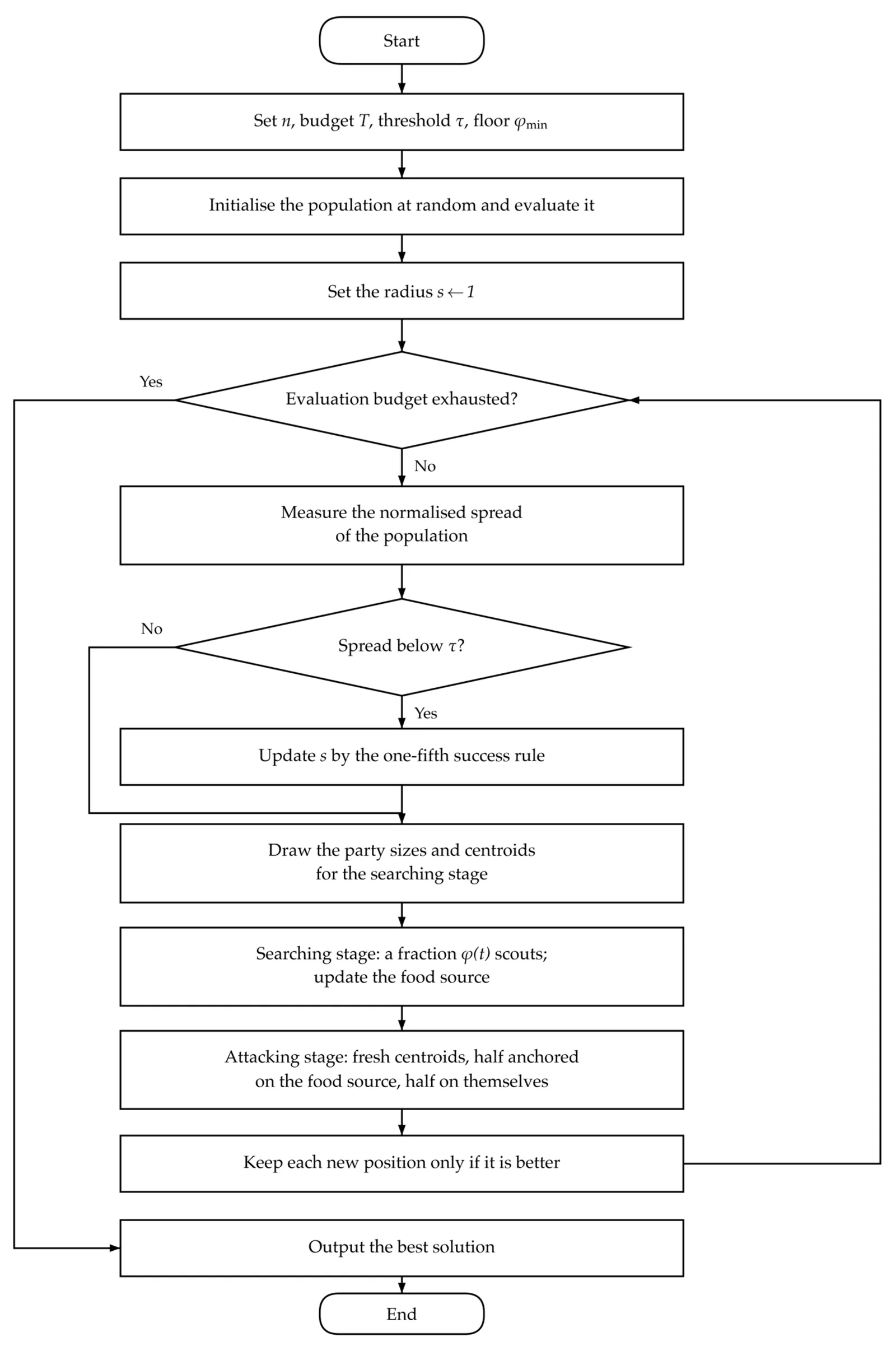
The flow of the proposed CRBMO, with the radius updated from the acceptance rates of the previous iteration.

Algorithm 1 labels the three repairs as the dual-anchor attack (DAA), the collapse-triggered radius control (CRC) and the adaptive foraging allocation (AFA). Figure 3 gives the flow of CRBMO and Algorithm 1 states it step by step. Beyond the parameters inherited from RBMO the algorithm has two: the collapse threshold *τ* and the scouting floor *ϕ*_min_. Section 5.2 reports their sensitivity. Every algorithm in this paper, CRBMO included, is run from one implementation, through one evaluation wrapper, with seeds fixed as a function of algorithm, function, dimension and run index, so that every number reported can be regenerated exactly [24]; the code and the raw results are provided in the Supplementary Materials.
**Algorithm 1:** Collapse-Resistant Red-billed Blue Magpie Optimizer (CRBMO)1:**Input:** objective *f*, bounds **lb**, **ub**, population size *n*, evaluation budget *T*, collapse threshold *τ*, scouting floor *ϕ*_min_2:**Output:** best solution **x**_food_ and *f*(**x**_food_)3:Initialise the population by Equation (1) and evaluate it; set *s* ← 14:**while** the evaluation budget is not exhausted **do**5:Record **x**_food_, the best individual of the population6:Compute the normalised spread by Equation (6); the radius control is active only if it is below *τ*//CRC, Section 4.27:Draw a party size for every individual and form the party centroids8:Build the searching candidates by Equation (2), with the displacement of Equation (8) if the radius control is active9:Let a fraction *ϕ*(*t*) of Equation (10) scout; evaluate only their candidates and accept by Equation (5)//AFA, Section 4.310:Compute *CF* by Equation (3) and draw the anchors of Equation (7) //DAA, Section 4.111:Build the attacking candidates by Equation (7), with the displacement of Equation (8) if the radius control is active; evaluate them and accept by Equation (5)12:**if** the radius control is active **then** update *s* by Equation (9)13:**end while**14:**return x**_food_ and *f*(**x**_food_)

### 4.5. Computational Complexity

Let *n* be the population size, *D* the dimension and *T* the evaluation budget. RBMO spends *O*(*nD*) on initialisation and, per iteration, *O*(*n*^2^log *n*) on the party draws and *O*(*nD*) on the position updates, over *T*/(2*n*) iterations, giving Equation (11).

(11) ORBMO=OnD+T2nn2log n+nD=OTD+Tnlog n

CRBMO adds three terms, all of them cheap. The dual anchor is one Bernoulli draw and one selection per individual, *O*(*nD*) per iteration. The spread of Equation (6) is *O*(*nD*) per iteration and the one-fifth update is *O*(1). The scouting mask is *O*(*n*), and because it removes evaluations rather than adding them the number of iterations rises from *T*/(2*n*) to $T/\left((\bar{\phi }+1)n\right)$ with $\bar{\phi }<1$ the mean scouting fraction. The total is therefore Equation (12).

(12) OCRBMO=OnD+T(ϕ¯+1)nn2log n+nD=OTD+Tnlog n

which is the same order as RBMO. Section 5.6 reports the measured cost, which is what matters when an objective evaluation is a policy simulation rather than a closed-form function.

## 5. Benchmark Evaluation

### 5.1. Experimental Setting

All experiments were run on a workstation with 16 CPU cores and 64 GB of memory under macOS, in Python 3.13 with NumPy and SciPy; all experiments were executed in Python, and MATLAB was not used. The benchmark is the CEC2017 real-parameter suite [25], used in full: 29 functions, of which F1 and F3 are unimodal, F4–F10 simple multimodal, F11–F20 hybrid and F21–F30 composition. F2 is excluded, as the competition’s own definitions exclude it, because it shows unstable behaviour at higher dimensions and large performance variation between implementations of the same algorithm [25]. The search domain is [−100,100]*^D^* and the dimension is set to 10, 30, 50 and 100. Every algorithm of Section 5.1, Section 5.2, Section 5.3, Section 5.4, Section 5.5 and Section 5.6 uses a population of *n* = 30 and a budget of 30,000 function evaluations, and is run 30 times independently on every function; the reported quantity is the error *f*(**x**_best_) − f^*^. Every algorithm is driven through the same evaluation wrapper, which counts evaluations and stops the run at exactly the same budget; every candidate is kept in the box by clipping, except in the three evolutionary algorithms of Section 5.8: L-SHADE and jSO use the midpoint repair of their source publications, and CMA-ES maps its samples into the box through the default boundary transformation of its reference implementation (pycma 4.5.0). Across all 34,800 runs of Section 5.4 no non-finite result occurred.

The suite is evaluated by the competition’s own released C implementation, called through a thin binding, rather than by a reimplementation. An earlier draft of this study used a widely installed Python package for the suite, and checking it against the released C code function by function showed that it reproduces only three of the 29: it pairs the renumbered function forms of the updated definition report with the data files of the original release, and several of its definitions omit the per-function domain scaling the official code applies before rotation. Every number in this paper was regenerated after that was found. The binding is checked against a separately compiled standalone build of the same source and agrees with it to the last bit on all 29 functions in all four dimensions, and 28 of the 29 attain the official optimum value exactly at the official shift vector, the exception being the Levy function, whose small residual is a property of the official code itself. The check is scripted and its output accompanies this paper.

CRBMO is compared with nine algorithms. The first is RBMO itself [4], so that every difference is attributable to the three strategies of Section 4. Three more are the recent cohort named in Section 1—the dung beetle optimizer [1], the secretary bird optimisation algorithm [2] and the black-winged kite algorithm [3]—all published within two years of RBMO and all reported to be competitive on CEC2017. The crested porcupine optimizer [26] is added as the strongest recent algorithm available to us. The remaining four are the baselines the field measures itself against: particle swarm optimisation [27,28], the grey wolf optimizer [29], the whale optimisation algorithm [30] and Harris hawks optimisation [31]. Section 5.8 adds L-SHADE [21], jSO [32] and CMA-ES [23], and three of the published RBMO variants of Section 2.3. Table 3 lists the parameter settings of CRBMO and the nine. For the nine they are the values of the source publications, except for two settings of PSO: its inertia weight [28] decreases linearly from 0.9 to 0.4, as is common practice, and its velocity clamp, which the inertia-weight formulation leaves open, is fixed at the conventional fifth of the box width. Of CRBMO’s parameters, *τ* and *ϕ*_min_ are set by the sweeps of Section 5.2, and *ε* keeps the value fixed before its own sweep. The algorithms added in Section 5.8 follow their source publications, as described there.

The grey wolf optimizer and the whale optimisation algorithm are included for their update equations, not for their metaphors; a component-level deconstruction has shown that what those two algorithms do is a particular parameterisation of operators that were already in the literature [8], and it is the operators that are being compared here. Every comparator except RBMO was reimplemented in the same vectorised style: the whole population is updated from its pre-iteration positions, whereas the released MATLAB source files, which were consulted as code and not executed, update one individual at a time and so let an individual see moves already made in the same pass. Where a published description and its released code disagree, the choice made here is recorded in the module that implements it, and the modules accompany this paper. For RBMO, the algorithm this paper repairs, the choice was measured: against a line-by-line transcription of its released code, over 12 representative functions at *D* = 30 and ten runs, the vectorised form is worse on three of them at the 0.05 level and better on none. Every comparison in this paper therefore uses RBMO in the sequential form of its authors’ own code, including its food-storage rule; the vectorised form, on which CRBMO is built, appears only as variant V1 of the ablation in Section 5.3, where the three strategies have to be isolated on a common base. The statistical protocol follows current guidelines for comparing swarm and evolutionary algorithms [19,20]. Algorithms are ranked on each function by mean error and the ranks are aggregated by the Friedman test; the Iman–Davenport modification of the Friedman statistic is reported alongside the chi-square approximation, which is the more conservative of the two [19]. All-pairs comparison uses the Nemenyi critical difference, and control-versus-all comparison uses Holm’s step-down correction with CRBMO as the control, which controls the family-wise error rate over the nine comparisons that matter here [19,20]. Per-function differences are summarised with the Wilcoxon rank-sum test at the 0.05 level over the 30 runs, reported as win, tie and loss counts; because repeated pairwise tests do not control the family-wise error rate, those counts describe where a difference lies and are not a second inferential layer [19]. Two design choices follow from recent criticism of this literature: every claim below is accompanied by the dimension and function group it holds on, because a re-evaluation of eleven highly cited recent proposals against a strong baseline found most of the reported advantage did not survive [33]; the suite is used in its shifted and rotated form and is followed in Section 6 by two problems whose optimum is unknown, because results on functions whose optimum sits at the centre of the search box do not transfer [34].

### 5.2. Parameter Sensitivity

**Figure 4:**
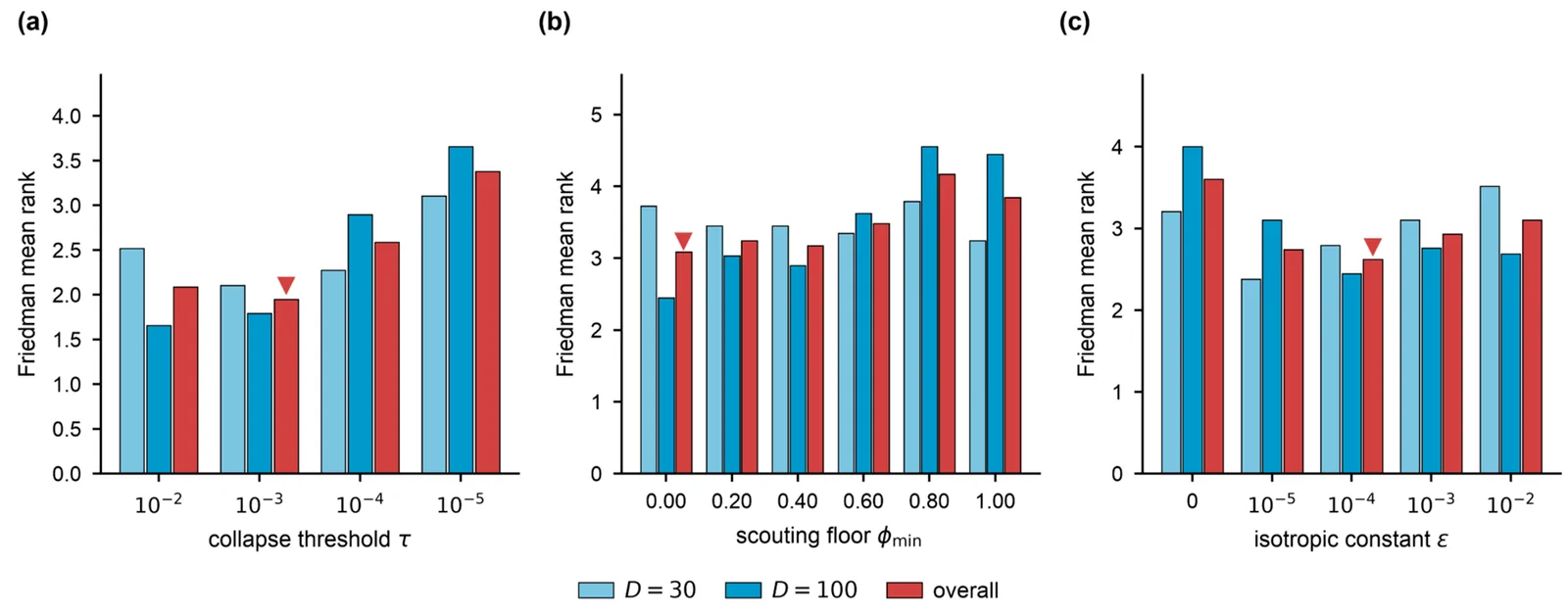
The parameter sensitivity of CRBMO on the CEC2017 suite: (**a**) collapse threshold *τ*; (**b**) scouting floor *ϕ*_min_; (**c**) isotropic constant *ε*. Red inverted triangles mark the value with the minimum overall Friedman rank in each sweep, which is not necessarily the adopted default.

CRBMO adds two parameters of its own: the collapse threshold *τ* of Section 4.2, which decides when the radius control wakes up, and the scouting floor *ϕ*_min_ of Equation (10), which decides how far the searching stage is allowed to be run down. The constants of the one-fifth success rule take their classical values and are not tuned; the constant *ε* of the isotropic term in Equation (8) is swept as well, over five values including *ε* = 0, which removes the term. Each parameter is swept with the others held at their defaults and everything else unchanged, over the 29 CEC2017 functions at *D* = 30 and *D* = 100 with 30 runs per function. Table 4, Table 5 and Table 6 give the Friedman mean ranks and Figure 4 plots them.

The parameters behave differently, and the difference is informative. The four values of *τ* span 1.43 rank units, 0.48 per step of the grid, and *τ* = 10^−3^ is best; *τ* = 10^−2^ is within the critical difference of 0.62 of it, so the control is insensitive to when exactly it is switched on within that range, whereas waking it later costs, because by then the population it is meant to rescue has already contracted. The six values of *ϕ*_min_ span 1.09 rank units, 0.22 per step, with *ϕ*_min_ = 0.00 best and an upward but not monotone trend; only *ϕ*_min_ = 0.80 lies outside the critical difference of the best. Read as a statement about the algorithm rather than as a tuning result, the second sweep says that once the radius control supplies the step scale, the undirected searching stage has less left to contribute late in a run, and every evaluation kept in it is an evaluation taken away from the attack. The five values of *ε* span 0.98 rank units (critical difference 0.80), with *ε* = 10^−4^ best overall. Removing the isotropic term altogether (*ε* = 0) gives an overall rank of 3.60 against 2.93 for the default (3.21 against 3.10 at *D* = 30 and 4.00 against 2.76 at *D* = 100). Without the term CRBMO still beats RBMO, the algorithm it repairs, on 18 and 22 of the 29 functions at *D* = 30 and *D* = 100 by the Wilcoxon rank-sum test and loses on three and zero, against 19 and 25 wins and one and zero losses for the default in the same sweep: the term adds to the gain at *D* = 100 but does not produce it. The defaults used everywhere else in this paper are *τ* = 10^−3^, *ϕ*_min_ = 0 and *ε* = 10^−3^; *ε* keeps the value fixed before its sweep, because the best value, *ε* = 10^−4^, is 0.31 rank units ahead of it, within the critical difference of 0.80.

### 5.3. Ablation Study

**Figure 5:**
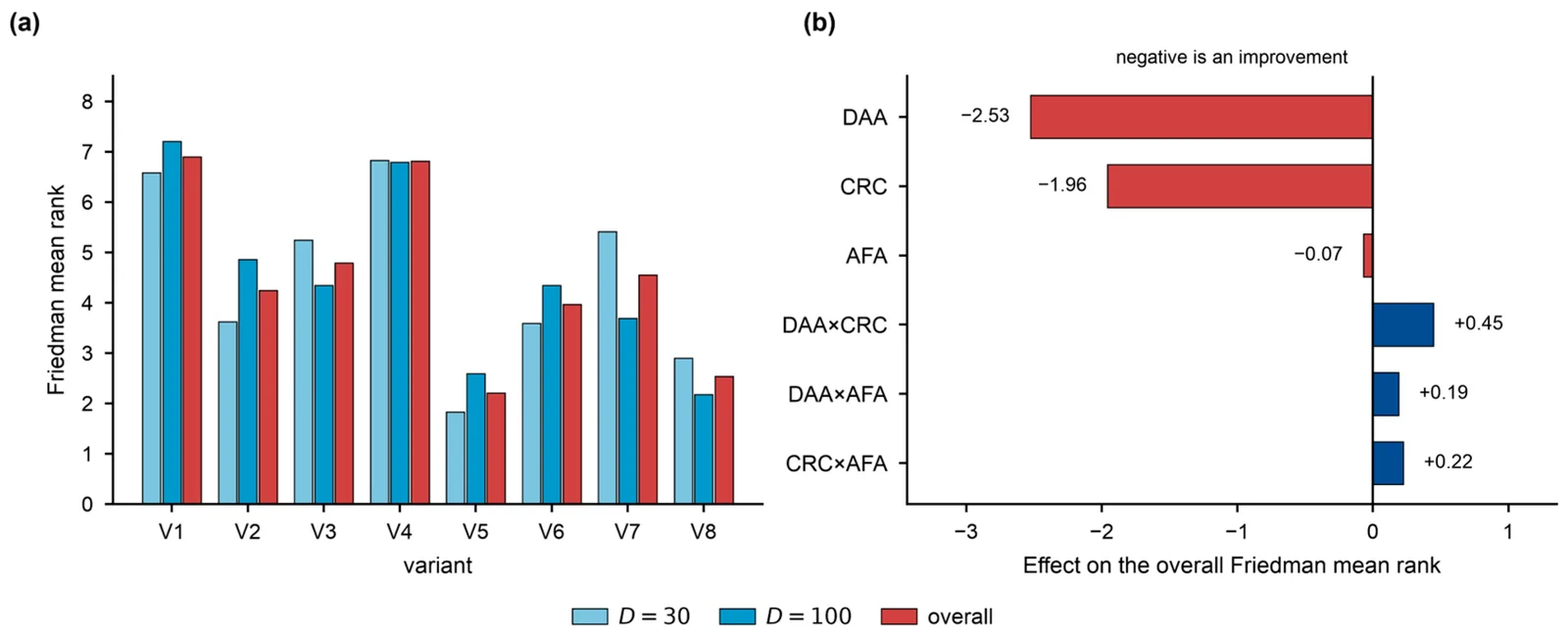
Ablation of the three strategies: (**a**) the Friedman mean rank by variant and dimension; (**b**) the factorial effects on the overall rank.

The three strategies are switched on and off in all eight combinations, giving the complete 2^3^ factorial design of Table 7. Every variant is run over the 29 CEC2017 functions at *D* = 30 and *D* = 100 with 30 independent runs, under exactly the protocol of Section 5.1. A factorial design is used rather than the customary sequence of one-at-a-time removals because the three strategies are not independent by construction: the radius control replaces a step scale that the dual-anchor attack has already changed, and the foraging allocation moves evaluations between the two stages the other two strategies act on. The customary one-at-a-time ablation reports each strategy against the full algorithm and reads the difference as that strategy’s contribution; the factorial design reports the same main effects and, in addition, the interactions those differences confound. Table 8 gives the resulting Friedman mean ranks and Figure 5 plots them together with the factorial effects.

Table 8 gives the result. The best variant is V5 at 2.21, with the full algorithm V8 second at 2.53—a gap of 0.33 rank units against a Nemenyi critical difference of 1.38, so the two are not distinguishable—and the base algorithm V1 last at 6.90, a separation of 4.36 rank units between the base algorithm and the full one on an eight-point scale. The factorial effects are more useful than the ordering. Averaged over the other two strategies, switching on the dual-anchor attack moves the mean rank by −2.526, the radius control by −1.957 and the foraging allocation by −0.069, where a negative number is an improvement.

Two of those three numbers are large and one is not. The dual-anchor attack and the radius control each account for most of the gain on their own, which is what Section 3 predicts, since they are the two halves of the feedback loop: one stops the attack from destroying the spread, the other stops the step scale from depending on it. The foraging allocation is worth 0.069 rank units averaged over the design, which on an eight-point scale is nothing. It is not uniformly inert, though; its effect is +0.36 at *D* = 30, −0.50 at *D* = 100, so of these two dimensions it pays only at *D* = 100. That is the dimension at which the collapse of Section 3 bites hardest, which is where reallocating evaluations away from an undirected stage should be worth most. The three interactions are +0.448 for DAA-CRC, +0.190 for DAA-AFA and +0.224 for CRC-AFA—all positive, meaning the strategies overlap rather than compound. They repair one loop from three sides, and having repaired it once there is less left for the next to fix.

Because V5, which omits the foraging allocation, ranks ahead of the full algorithm, the allocation needs a justification of its own, and two further experiments provide it. The complete design was repeated at *D* = 10 and *D* = 50 under the same protocol. The main effect of the allocation on the Friedman rank is +0.77 at *D* = 10, +0.36 at *D* = 30, −0.02 at *D* = 50 and −0.50 at *D* = 100; it costs at low dimension, pays at high dimension and falls monotonically in between, which is the trend expected of a device that moves evaluations away from an undirected stage as the collapse of Section 3 bites harder. Compared directly by the Wilcoxon rank-sum test, V8 is worse than V5 on six, six, two and two of the 29 functions at *D* = 10, *D* = 30, *D* = 50 and *D* = 100, and better on one, two, one and seven. On the eight instances of Section 6, whose dimensions run from 80 to 850, V8 and V5 are statistically indistinguishable on all eight. The allocation is therefore retained for the high-dimensional problems this paper targets, where it is neutral or beneficial; for problems of dimension 30 or less, V5 is the better choice.

### 5.4. Comparison with Swarm Algorithms

CRBMO is now compared with the nine algorithms of Table 3 over the whole CEC2017 suite in four dimensions, 30 runs each. The complete error statistics—mean and standard deviation for every algorithm, function and dimension—are given in Table A1, Table A2, Table A3 and Table A4 of Appendix A; this section reports what they add up to.

**Figure 6:**
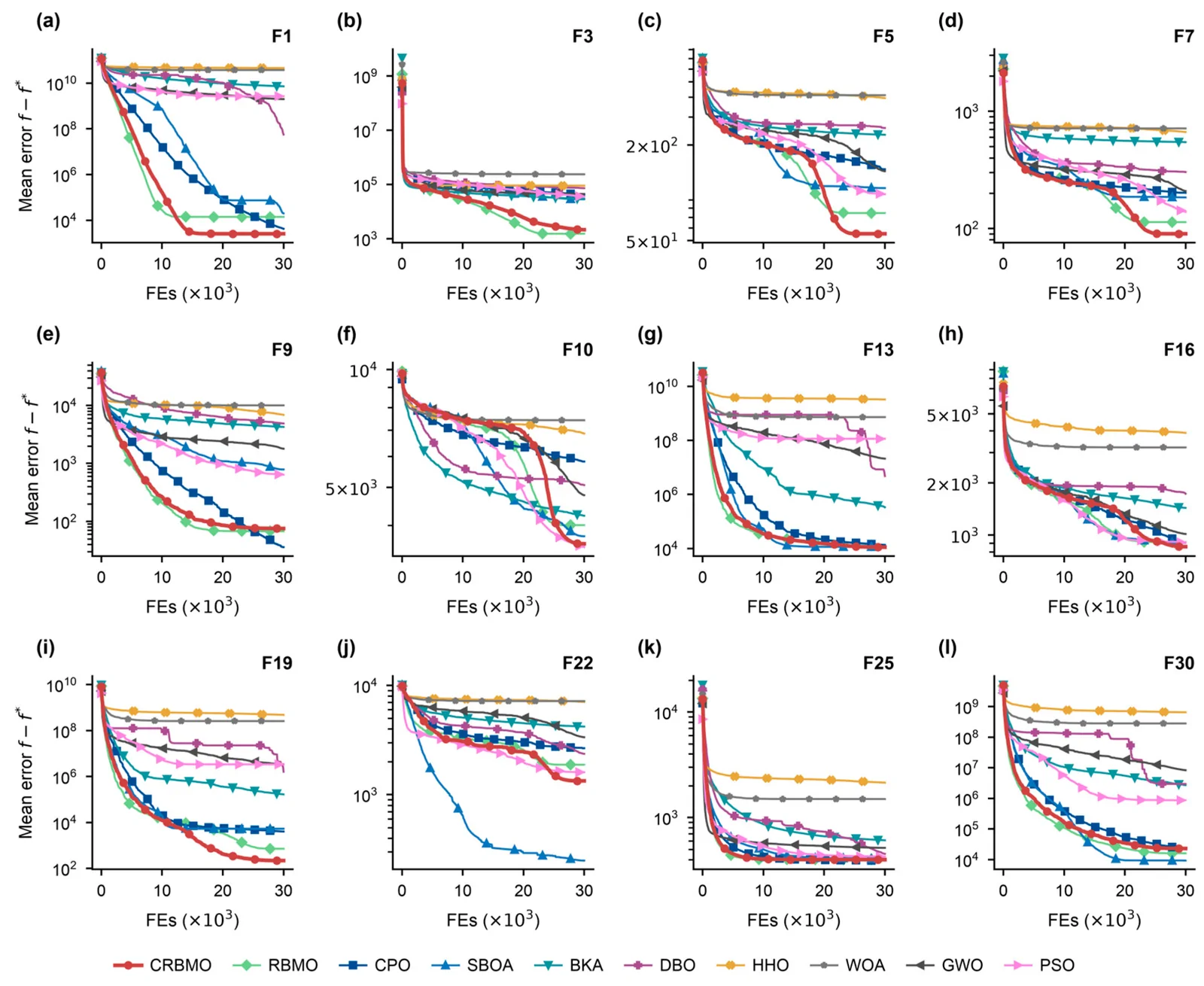
The convergence curves of CRBMO and the comparison algorithms on twelve representative CEC2017 functions (*D* = 30).

**Figure 7:**
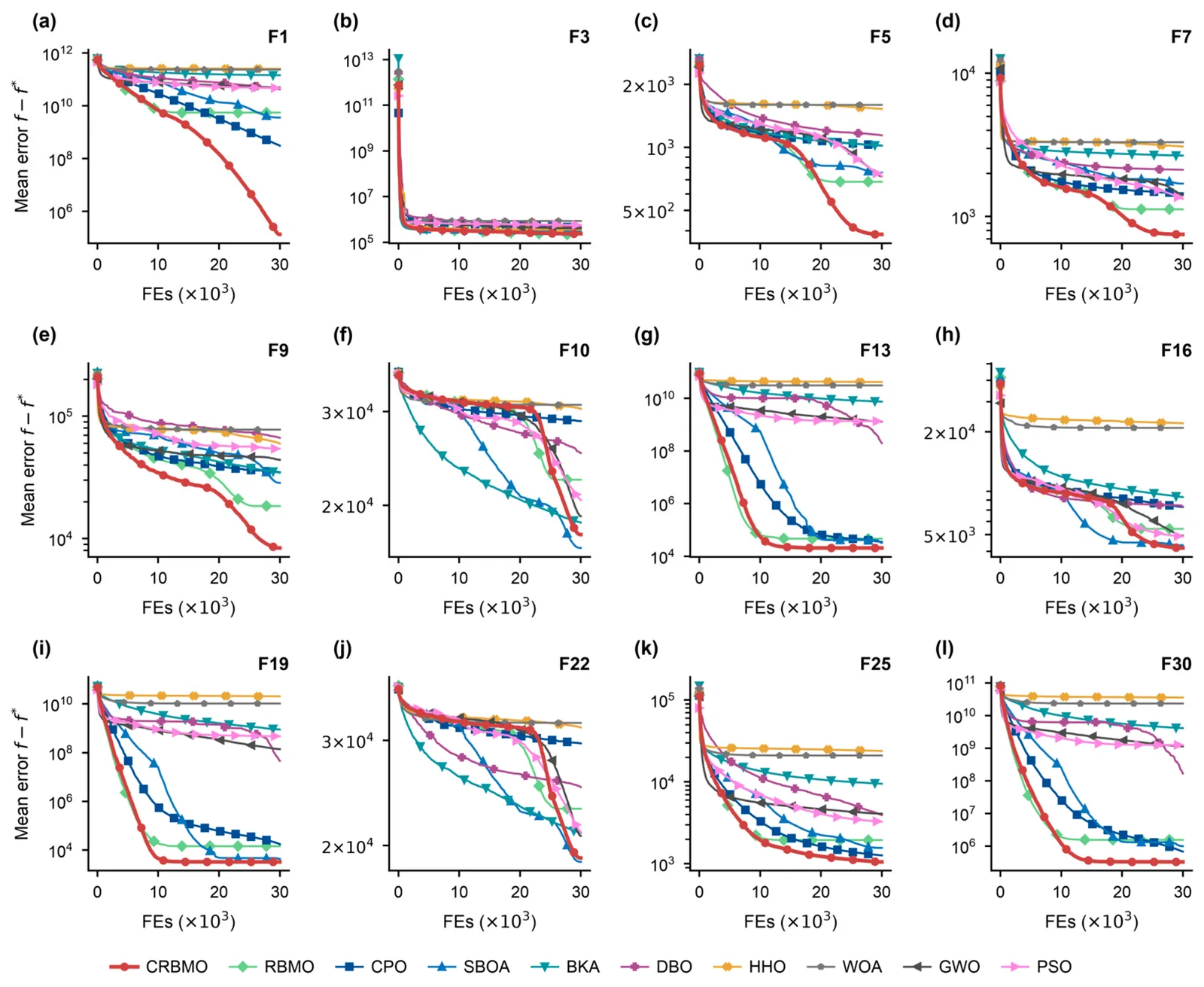
The **c**onvergence curves of CRBMO and the comparison algorithms on the same twelve CEC2017 functions (*D* = 100).

**Figure 8:**
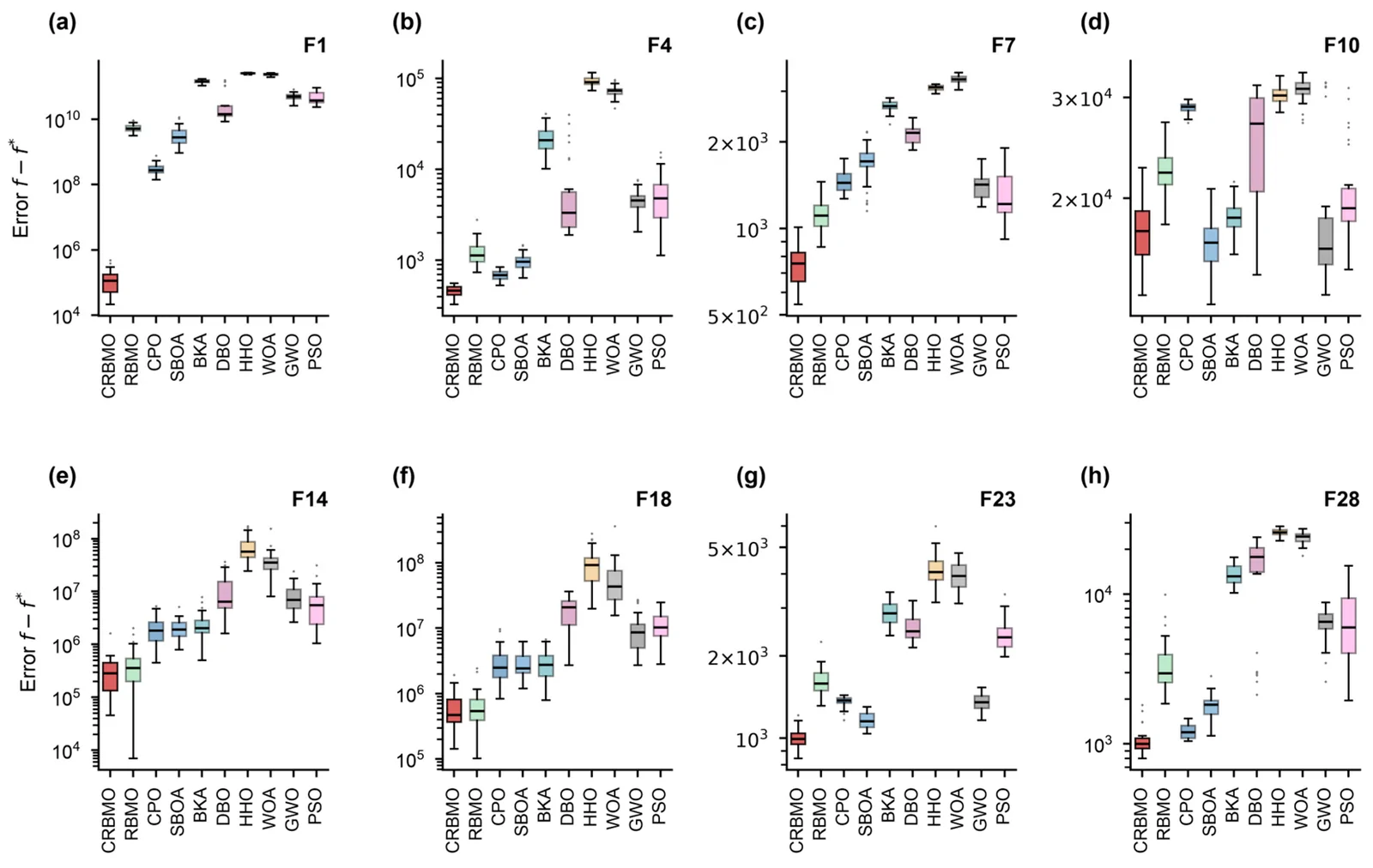
The distribution of the final error of CRBMO and the comparison algorithms on eight representative CEC2017 functions (*D* = 100).

**Figure 9:**
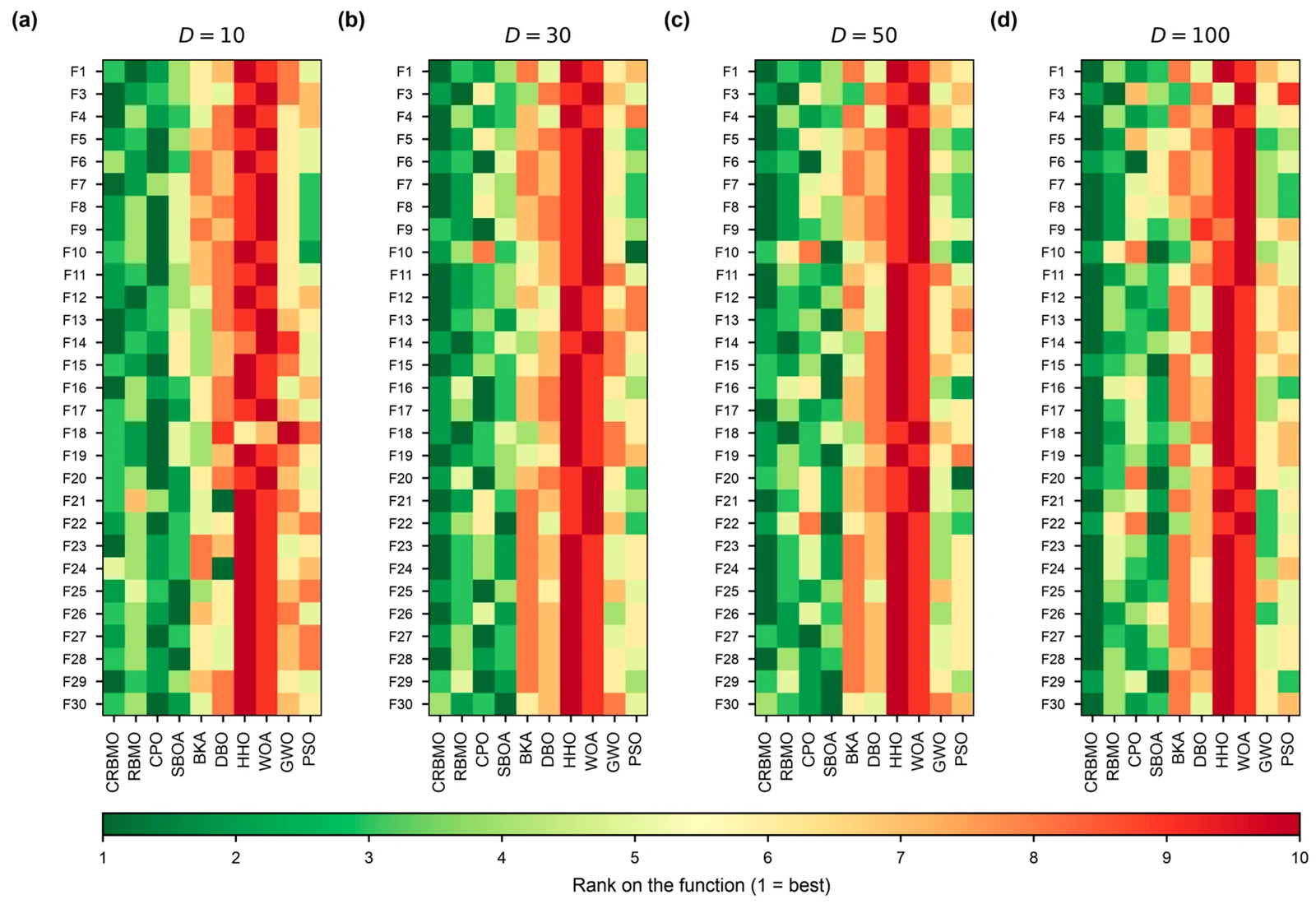
Ranking heatmaps of CRBMO and the comparison algorithms on the CEC2017 suite: (**a**) *D* = 10; (**b**) *D* = 30; (**c**) *D* = 50; (**d**) *D* = 100.

Figure 6 and Figure 7 show convergence curves on twelve representative functions at *D* = 30 and *D* = 100, Figure 8 the distribution of the final error at *D* = 100 and Figure 9 the per-function rankings in all four dimensions.

Table 9 collects the Friedman mean ranks. CRBMO is first at *D* = 30, *D* = 50 and *D* = 100 and first overall. Against RBMO the improvement is 1.43 rank units, and it grows with dimension: 1.03 at *D* = 10, 1.24 at *D* = 30, 1.31 at *D* = 50, and 2.14 at *D* = 100. That is the pattern Section 3.3 predicts, because the loop CRBMO breaks closes earlier, in useful-progress terms, the larger the box is.

**Figure 10:**
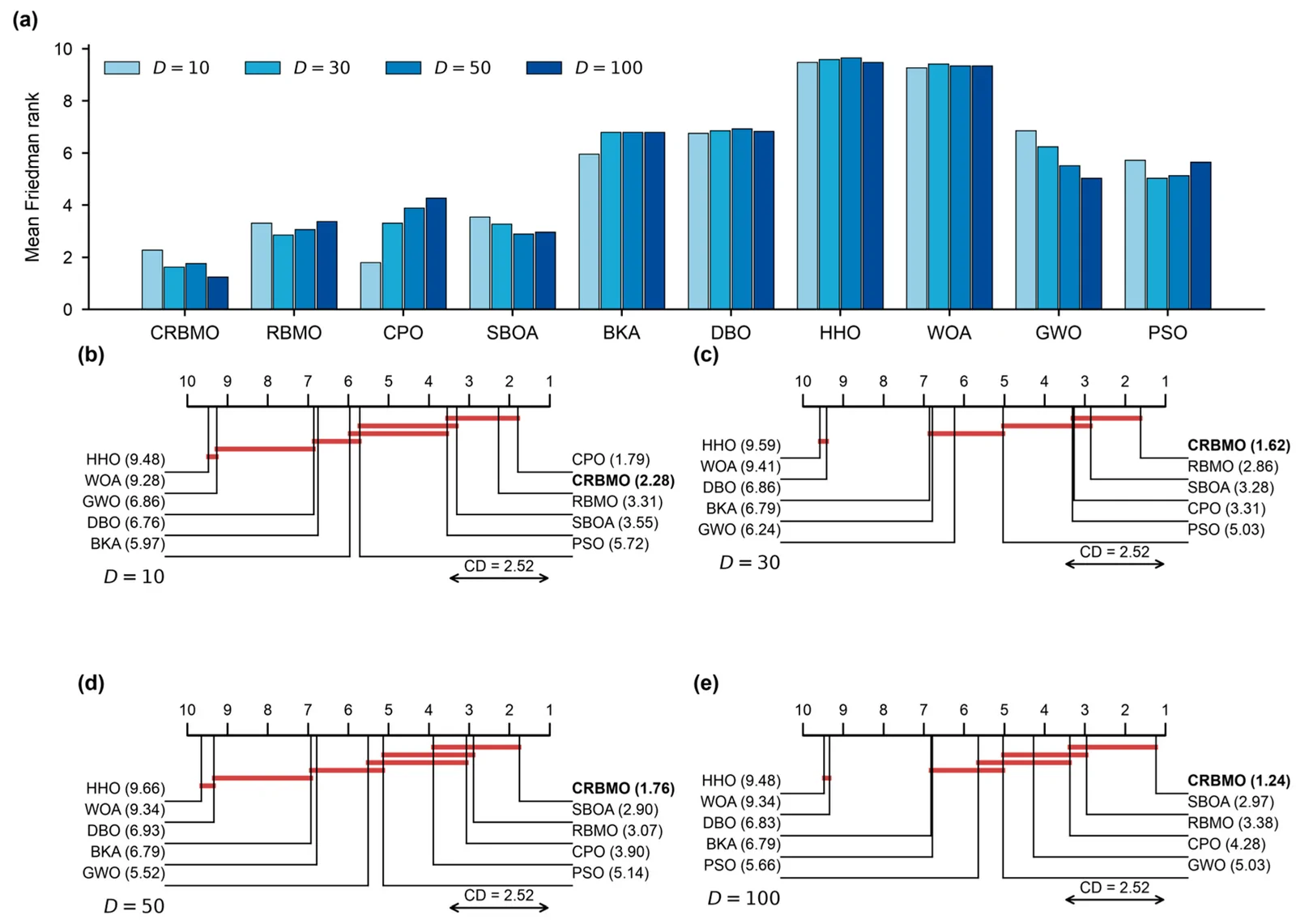
The Friedman ranks and Nemenyi post hoc test of CRBMO and the comparison algorithms (*α* = 0.05): (**a**) mean ranks by dimension; (**b**–**e**) *D* = 10,30,50,100. Bold labels identify the proposed CRBMO and do not indicate the best rank in every dimension.

The Friedman test rejects the hypothesis that the ten algorithms are equivalent in every dimension and over the pooled suite (*χ*^2^ = 813.7, *p* < 0.001; Iman–Davenport *F* = 406.3, *p* < 0.001), so the mean ranks may be read. Table 10 breaks them down by function group and Figure 10 shows the Nemenyi critical-difference diagrams.

A Nemenyi diagram compares all ten algorithms with one another and is therefore conservative. Holm’s step-down procedure with CRBMO as the control tests only the nine comparisons that matter here. Taking each dimension on its own, so that the 29 functions are the units and no pooling is involved, the picture sharpens with dimension: six at *D* = 10, six at *D* = 30, seven at *D* = 50, and all nine at *D* = 100, including against RBMO (*p*_Holm_ = 0.014). Pooled over all four dimensions it rejects all nine. Pooling needs care. The pooled test treats each of the 116 function–dimension pairs as a unit, which counts every function four times. Repeated with the standard error of 29 units, the number of distinct functions, it leaves six of the nine rejections standing and weakens the comparison against RBMO to *p*_Holm_ = 0.137. The claim this paper rests on is therefore the per-dimension one, which uses 29 units and needs no pooling at all. On this suite at 30,000 evaluations, Holm’s procedure separates CRBMO from all nine swarm comparators, RBMO included, only at *D* = 100, the dimension at which the collapse costs most. At that dimension it does not separate CRBMO from ERBMO or CMA-ES (Section 5.8), and at the standard budget it does not separate CRBMO from RBMO in any dimension (Section 5.7).

**Figure 11:**
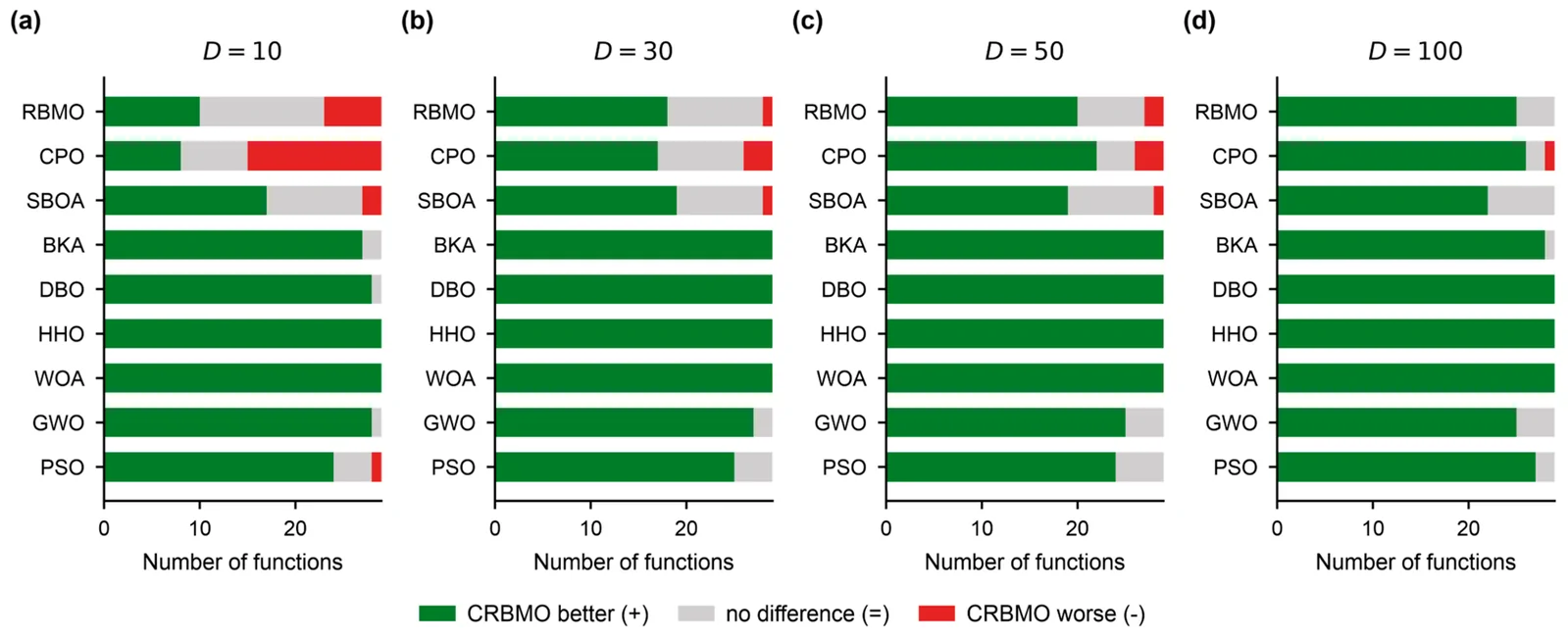
The Wilcoxon rank-sum outcomes of CRBMO versus the comparison algorithms (*α* = 0.05): (**a**) *D* = 10; (**b**) *D* = 30; (**c**) *D* = 50; (**d**) *D* = 100.

The Friedman test is a statement about the suite; the Wilcoxon rank-sum test is a statement about a function. Table 11 and Figure 11 give the per-function outcome at the 0.05 level. Against RBMO, CRBMO wins 73, ties 34 and loses nine of the 116 function–dimension pairs, and at *D* = 100 the record is 25/4/0. Against CPO the record is less one-sided: 73/22/21, with 14 of the losses at *D* = 10; against SBOA it is 77/35/4. Over the nine comparisons together CRBMO wins 888 and loses 35 of 1044 pairs.

### 5.5. Search Behaviour

**Figure 12:**
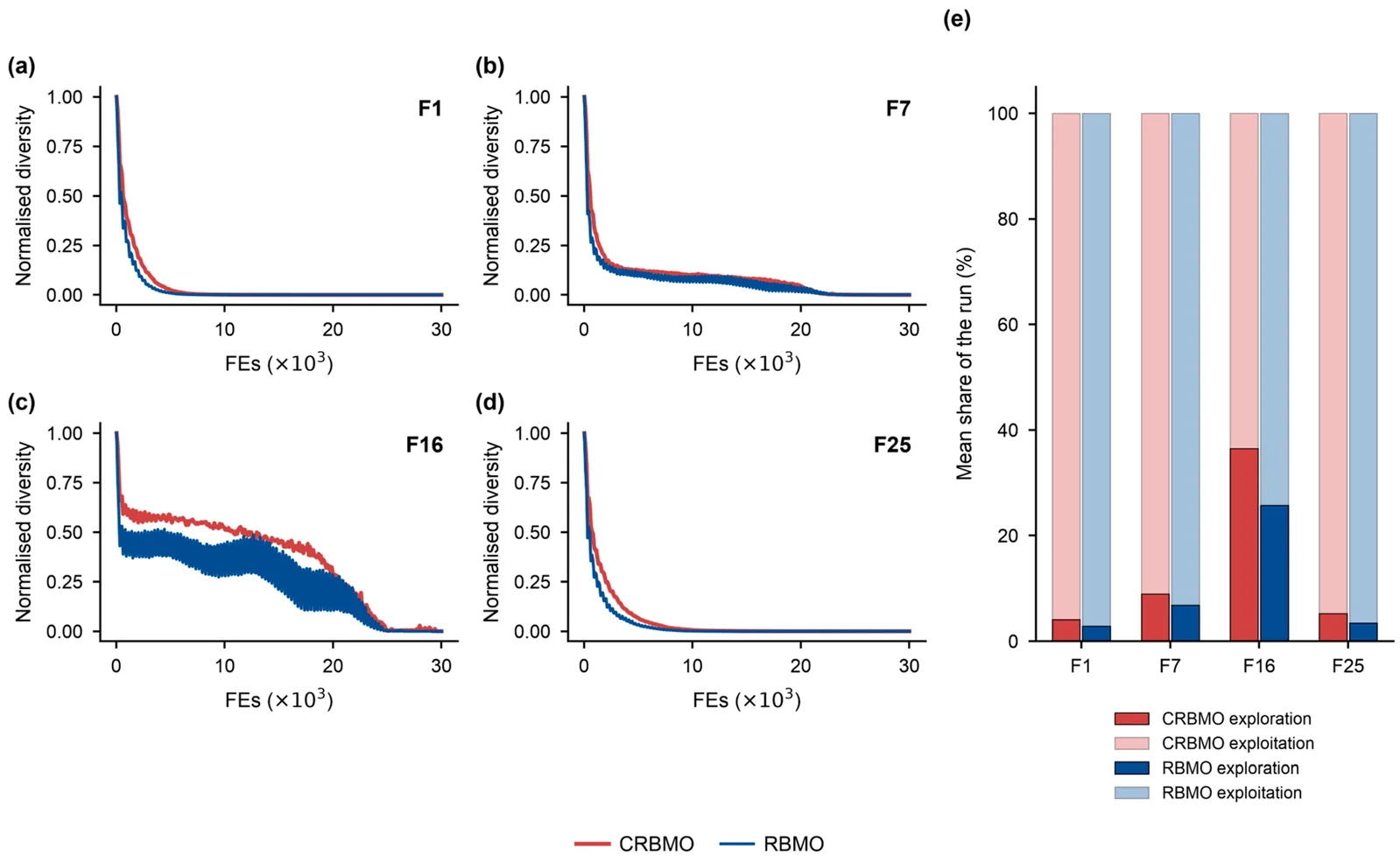
Search behaviour of CRBMO and RBMO (*D* = 30): (**a**–**d**) normalised population diversity on F1, F7, F16 and F25; (**e**) exploration and exploitation shares.

The ranks say which algorithm is better; they do not say what it is doing differently. The diversity decomposition of Morales-Castaneda et al. [35] separates a run into an exploring part and an exploiting part from the population itself. Population diversity is measured as the mean normalised deviation from the dimension-wise median,

(13) $$\mathrm{Div}(t)=\frac{1}{\mathrm{nD}}\sum_{i=1}^{n}\sum_{j=1}^{D}\frac{\left|x_{\mathrm{ij}}^{t}-{\mathrm{med}}_{j}\left(X^{t}\right)\right|}{ub_{j}-lb_{j}}$$

(14) $$\mathrm{XPL}\%(t)=100\frac{\mathrm{Div}(t)}{{\mathrm{Div}}_{\max}},\;\;\;\mathrm{XPT}\%(t)=100\frac{\left|\mathrm{Div}(t)-{\mathrm{Div}}_{\max}\right|}{{\mathrm{Div}}_{\max}}$$

where Div_max_ is the largest diversity seen during the run. Figure 12 applies the decomposition to CRBMO and RBMO on one function from each CEC2017 group at *D* = 30.

The two algorithms differ in the way the diagnosis predicts. RBMO’s diversity falls steeply at the start of the run and drops below 1% of its initial value for good at 17% and 24% of the run on F1 and F25 and at 74% and 82% on F7 and F16, ending between 9.1 × 10^−20^ and 7.8 × 10^−18^ of the box width, the level of machine precision; its exploration share over the whole run is 2.8% on F1, 6.8% on F7, 25.7% on F16, and 3.5% on F25. CRBMO’s falls too—it is a converging optimiser, not a random walk—but it levels off well above machine precision, ending between 4.8 × 10^−11^ and 3.3 × 10^−6^, and it gives exploration shares of 4.1%, 8.9%, 36.5%, and 5.3% on the same four functions, a factor of 1.3 to 1.5 higher. The difference is not that CRBMO explores more in the usual sense of covering the box; it is that CRBMO still has a length scale to explore with at the end of the run, because that scale is now supplied by a controller instead of by the population.

### 5.6. Computational Cost

The three strategies add no objective evaluation and no operation whose cost grows faster than the operators already present: the dual-anchor attack replaces one broadcast subtraction by another, the radius control maintains three scalars, and the foraging allocation draws one Bernoulli mask per iteration. The complexity therefore remains *O*(*TD* + *Tn*log *n*), as derived in Equations (11) and (12). The measured cost agrees. Averaged over all 3480 runs, CRBMO takes 1.47 s, the longest of the nine vectorised implementations (0.52–1.03 s for the other eight), and 1.35 times its vectorised base V1 (1.09 s), the implementation from which it differs only by the three strategies. RBMO in the sequential form of its released code takes 3.88 s, but that reflects its per-individual loop rather than the algorithm. In a real application the objective function dominates these differences by orders of magnitude.

### 5.7. Behaviour at the Full CEC2017 Budget

Every experiment above uses 30,000 evaluations, which at *D* = 100 is 300 evaluations per dimension. The CEC2017 definition sets the budget at 10,000*D* evaluations [25], between 3.3 and 33 times as many. CRBMO and RBMO were therefore re-run at 10,000*D* evaluations on the 29 functions in all four dimensions, with every parameter unchanged and 10 runs per function, together with the three evolutionary algorithms of Section 5.8, L-SHADE, jSO and CMA-ES, whose population rules scale with the budget as their sources prescribe. The Friedman test rejects equivalence in every dimension (*χ*^2^ from 59.1 to 92.9, *p* < 0.001), and Table 12 gives the mean ranks with the Wilcoxon record of CRBMO against each algorithm.

At the standard budget CRBMO has a better mean rank than RBMO at *D* = 30, *D* = 50 and *D* = 100 and the same mean rank at *D* = 10, and it wins 28, ties 78 and loses 10 of the 116 Wilcoxon comparisons with it. The rank gap is 0.00, 0.48, 0.62 and 0.45 from *D* = 10 to *D* = 100, so it grows up to *D* = 50 but not beyond, unlike at 30,000 evaluations, and Holm’s procedure does not separate the two in any dimension. Against the evolutionary algorithms the picture is different from that at 30,000 evaluations: CRBMO has the best mean rank in no dimension; jSO ranks first in all four dimensions; and overall CRBMO ranks fourth, behind jSO, L-SHADE and CMA-ES. L-SHADE, jSO and CMA-ES rank ahead of CRBMO in every dimension, and by the Wilcoxon test CRBMO loses to them on 103, 109 and 72 of the 116 function–dimension pairs respectively; Holm’s procedure separates L-SHADE and jSO from CRBMO in all four dimensions. This is the budget at which L-SHADE and jSO were entered in the CEC 2014 and 2017 competitions, whereas the parameters of CRBMO were set by the sweeps of Section 5.2 at 30,000 evaluations. CRBMO and RBMO both use the extra budget: the mean error of RBMO is lower by more than a tenth than at 30,000 evaluations on 97 of the 116 function–dimension pairs, and that of CRBMO on 79. The extra evaluations do not dissolve RBMO’s collapse, however: in the instrumentation of Section 3.2 its stagnant share stays the same or grows on four of the five functions at ten times the budget (Section 3.1). The instrumentation also shows where the radius control fails. At ten times the budget CRBMO’s radius reaches its lower bound in 30 of 30 runs on F5, 23 on F28 and eight on F15. The spread falls below the floor in 83% of the runs on F28 and 27% on F15, and in every run in which the radius also reaches its bound the spread reaches the floor first, by up to 5% of the budget; on F28 its stagnant stretch occupies 76% of the run, as long as RBMO’s. On F5 the spread stays above the floor in 97% of the runs, but a median of 87% of the late attacking candidates return exactly their parent’s value. Once the success rate stays below p^*^, the one-fifth rule contracts the radius geometrically, together with the population, down to its lower bound. In the runs where this happens the population collapses on F28 and F15, and on F5 the step becomes too small to change the candidates. On these functions the repair postpones the loop of Section 3.3 rather than removing it. The largest loss at ten times the budget is on F1, where the radius never reaches its bound. The population contracts to a spread of about 2 × 10^−7^ within the first tenth of the run; from 20% to 70% of the run the rule holds *α_t_* between 0.18 and 0.24 and the radius between 4.2 × 10^−5^ and 1.4 × 10^−4^, and the radius then grows by more than four orders of magnitude, to about six at the end of the run, as *CF* falls to zero. The error hardly moves, ending at a median of 2571 against 0.39 for RBMO over the 30 instrumented runs. In the 10-run benchmark of Table 12, CRBMO’s mean error on F1 is above RBMO’s in every dimension (2045 against 0.61 at *D* = 30). F1 is the bent cigar function, whose condition number is 10^6^, and the isotropic term carries almost all of CRBMO’s late attacking step there (Section 4.2); a likely reason is that a single isotropic step scale cannot follow so elongated a valley, which RBMO’s population differences can.

### 5.8. Comparison with Evolutionary Baselines and Published RBMO Variants

Two further groups provide a stricter test than the nine swarm metaheuristics of Section 5.4: published improvements of RBMO, which modify the same base algorithm by other means, and evolutionary algorithms from outside the swarm family. The first group comprises CLD-RBMO [10], MRBMO [11] and ERBMO [18], three of the variants in Table 1, each implemented from its paper and, for ERBMO, from its authors’ released code. The other two could not be implemented faithfully: the IRBMO paper [9] leaves the constants of its chaotic map, its Levy step and one sampling distribution undefined, and a transcription of AR-RBMO [17] from its paper does not reproduce the published results on five of the eleven functions checked; neither has released code. The second comprises L-SHADE [21] and jSO [32], success-history-based differential evolution algorithms entered in the CEC 2014 and 2017 competitions, and CMA-ES [23] with independent restarts. All six were run under the protocol of Section 5.1, on the same 29 functions in four dimensions, with 30 runs each and a budget of 30,000 evaluations. The three evolutionary algorithms size their populations by their own published rules: L-SHADE starts from 18*D* individuals and jSO from 25Dln D, both reducing the population linearly to four over the run, and CMA-ES samples 4+⌊3ln *D*⌋ candidates per iteration. The Friedman test rejects the hypothesis that the eight algorithms are equivalent in every dimension (*χ*^2^ from 95.5 to 127.2, *p* < 0.001), and Table 13 gives their mean ranks with the Wilcoxon record of CRBMO against each.

CRBMO has a better mean rank than each of the three published variants in every dimension. Over the 116 function–dimension pairs it wins 82, 115 and 65 of the Wilcoxon comparisons against CLD-RBMO, MRBMO and ERBMO respectively, and loses seven, zero and 19. Holm’s procedure, with the 29 functions of each dimension as its units, separates CRBMO from MRBMO in all four dimensions and from CLD-RBMO at *D* = 50 and *D* = 100, but from ERBMO in none. The closest is ERBMO, whose rank improves at every step in dimension, from 5.76 at *D* = 10 to 3.21 at *D* = 100, where CRBMO’s is 2.28. None of the three has a better mean rank than RBMO itself in all four dimensions.

Against the evolutionary algorithms the ordering changes with dimension. At *D* = 10 jSO and L-SHADE rank first and second, and by the Wilcoxon test CRBMO is worse than them on 26 and 25 of the 29 functions respectively and better on none; Holm’s procedure separates both from CRBMO. At *D* = 30 and *D* = 50 CMA-ES ranks first and beats CRBMO on 21 and 23 of the 29 functions by the Wilcoxon test, although Holm’s procedure does not separate the two there. At *D* = 100 CRBMO has the best mean rank, 2.28 against 2.86 for CMA-ES, and Holm’s procedure separates it from L-SHADE and jSO but not from CMA-ES; by the Wilcoxon test, CMA-ES still beats CRBMO on 18 of the 29 functions and loses on 11. Pooled over the four dimensions, CRBMO ranks third, behind CMA-ES and jSO. The ranks of L-SHADE and jSO worsen at every step in dimension, from 2.19 and 1.64 at *D* = 10 to 5.52 and 4.34 at *D* = 100. Over the same range the fixed budget falls from 30% to 3% of the standard CEC2017 budget of 10,000*D* evaluations [25], while the initial populations of L-SHADE and jSO grow from 180 and 182 to 1800 and 1151 individuals. This experiment therefore cannot separate an effect of dimension from an effect of budget; Section 5.7 compares the three algorithms with CRBMO at the standard budget, where CRBMO no longer leads them at *D* = 100. On this evidence CRBMO improves on RBMO more than the published variants do, but at this budget it leads the evolutionary algorithms only at *D* = 100, and at the standard budget in no dimension.

## 6. Employment-Policy Applications

**Figure 13:**
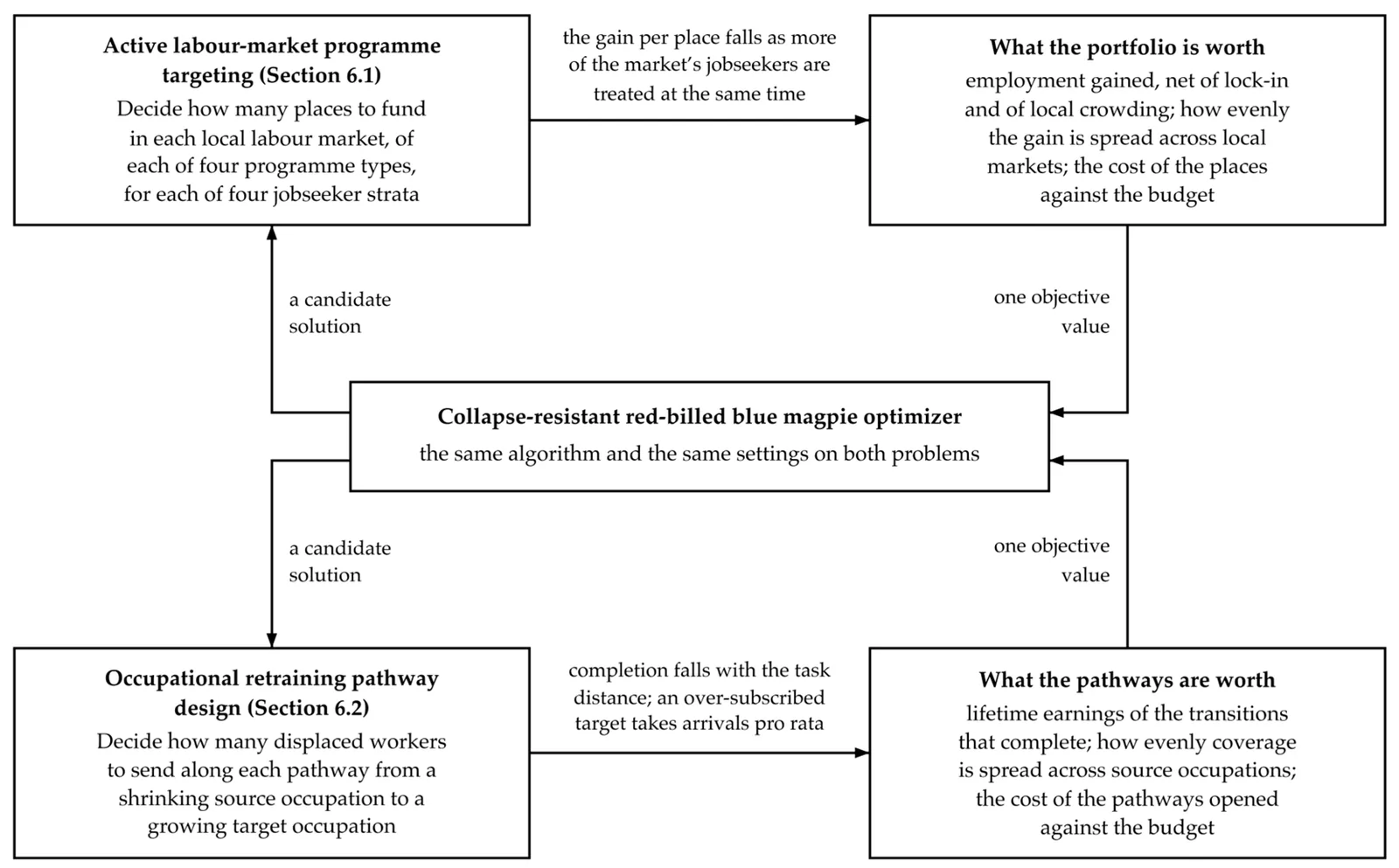
The two employment-policy design problems, what makes each objective non-separable, and how the optimiser enters them.

A benchmark suite shows that an algorithm searches well; it does not show what the algorithm is for. This section applies CRBMO to two design problems in employment policy. They are chosen because they are genuinely hard by the standards of the benchmark tests—both reduce, when stripped of their economics, to NP-hard combinatorial cores—and because the quantities in their objective functions are not invented: every coefficient is taken from a published quasi-experimental or meta-analytic estimate, cited where it is used. The first allocates an active labour-market programme budget across local markets, programme types and jobseeker strata. The second designs retraining pathways between occupations exposed to automation and occupations with unmet demand. Figure 13 shows how the optimiser enters each of them, and marks on each the feature of the objective that rules an exact method out.

In both applications the ten algorithms are run with exactly the parameter settings of Table 3 and exactly the protocol of Section 5.1: a population of 30, a budget of 30,000 objective evaluations and 30 independent runs on every instance. Nothing is retuned for the applications.

### 6.1. Active Labour-Market Programme Portfolio and Targeting

#### 6.1.1. The Planner’s Problem

The evidence on active labour-market programmes is rich and it is ex post. It tells a planner what a programme did for the people who received it, and it tells them that the answer depends on which programme and which people. It does not tell them how many places of which type to fund in which local market, and three features of the evidence make that question harder than a ranking exercise on average effects.

The first is lock-in. Participation suppresses employment while it lasts: participants’ employment probability runs about 15 percentage points below non-participants over the first three months and the gap closes only around month sixteen [36]. A programme that pays off at a two-year horizon can be a net loss inside a programming year, and the size of that loss differs by an order of magnitude between a two-week job-search workshop and a year of classroom training.

The second is that a place does not only help its holder. In a two-level randomised experiment in the Swedish public employment service, job-search assistance raised the treated jobseeker’s exit rate from unemployment and lowered it for untreated jobseekers at the same office [37]. The size of that absorption is governed by the local treatment rate rather than by the national total, which is what a two-step clustered experiment that randomised the treated share at 0, 25, 50, 75 or 100 per cent across 235 local labour markets was designed to identify [38]. This is not a correction to be applied after a per-programme evaluation: in the Danish activation experiment the stable-unit-treatment-value assumption fails outright, and non-participants’ outcomes move with the programme [39]. An objective function that sums per-programme effects over markets is therefore not a simplification of the policy problem but a different problem.

The third is that the benchmark a planner is measured against is the status quo, not random assignment. A causal-machine-learning assignment rule estimated on large German administrative records improves programme effects over observed practice by a margin that is real but bounded [40], which is the right order of magnitude to expect from better targeting and the right yardstick for what follows. The budget side is similarly pinned down: across the four providers of the randomised WorkAdvance sectoral-training demonstration, net programme cost per participant runs from about $4459 to $7527, against persistent earnings gains of 12% to 34% [41]; other sectoral programmes evaluated in the same literature cost more, so this is the lower end of the range a planner faces. Taken together, these features turn the choice of an allocation into a constrained search over assignment policies rather than an estimation problem.

Call this the active labour-market programme targeting problem, ALPT. A national agency has a fixed budget *B* for one programming year and must decide how many places to fund in each local labour market, of each programme type, for each group of jobseekers. Write *q*_𝓁_*_ks_* for the number of places funded in market 𝓁 ∈ {1,…,*L*}, of programme type *k* and for stratum *s*. The four programme types are job-search assistance, classroom training, private-sector wage subsidy and public employment, and the four strata are prime-age recipients of unemployment insurance, the long-term unemployed, young jobseekers and other disadvantaged jobseekers; these are the cells the meta-analytic evidence is reported in, so they are the cells a planner can act on. The decision variable is a vector of random keys in [0,1]*^L^*^×4×4^, decoded to integer places against a per-cell capacity and rounded down, with any cell below a viable size *q*^min^ set to zero. The objective has three parts: what the places buy, what they cost and how unevenly the gain is spread.

(15) $$\Delta e_{l\mathrm{ks}}=\sigma \left(\mathrm{logit}e_{ls}^{0}+\Delta_{\mathrm{ks}}\right)-e_{ls}^{0},\sigma (u)=\frac{1}{1+e^{-u}}$$

Programme effects are applied on the log-odds scale rather than as a percentage-point shift, so that a market with a high baseline cannot be pushed above one. Here $e_{ls}^{0}$ is the baseline employment probability of stratum *s* in market 𝓁 and Δ*_ks_* is the log-odds effect of programme *k* on stratum *s*, built additively from the programme-type and participant-group rows of the meta-analysis at the medium-run horizon.

(16) $$\rho_{l}(q)=\frac{\sum_{k,s}q_{l\mathrm{ks}}}{\sum_{s}u_{ls}},\kappa_{l}(q)=\max\left(1-\lambda \rho_{l}(q),0\right)$$

A place does not only help its holder. Where many jobseekers in one market are treated at once, part of the direct effect is absorbed by the untreated, so the gain is scaled by a congestion factor that falls with the local treatment rate *ρ*_𝓁_, the share of the market’s jobseekers who hold a place. The slope *λ* is set so that at a treated share of one half the displacement absorbs the measured fraction of the direct effect. Congestion is local, not national: the externality is indexed by 𝓁 because the experimental evidence randomises the treated share within a labour market.

(17) $$G(q)=\sum_{l,k,s}q_{l\mathrm{ks}}\Delta e_{l\mathrm{ks}}\kappa_{l}(q)-\frac{1}{2}\sum_{l,k,s}q_{l\mathrm{ks}}\frac{L_{k}}{12}$$

The gross gain is the sum over cells of places times congested effect. Against it is set the lock-in loss: a participant’s employment probability is depressed while the programme lasts, by an amount that differs sharply between a two-week job-search workshop and a year of classroom training. *L_k_* is the cumulated lock-in of programme *k* in employment-months and the one-half converts the cumulated loss to the share that falls inside the programming year.

(18) $$C(q)=\sum_{l,k,s}c_{k}q_{l\mathrm{ks}}+\sum_{l,k}F_{k}1\left[\sum_{s}q_{l\mathrm{ks}}\geq q^{\min}\right]$$

(19) $$\mathrm{CV}(q)=\frac{1}{\bar{g}}\sqrt{\sum_{l}w_{l}{\left(g_{l}(q)-\bar{g}\right)}^{2}},g_{l}=\frac{\sum_{k,s}q_{l\mathrm{ks}}\Delta e_{l\mathrm{ks}}\kappa_{l}}{\sum_{s}u_{ls}}$$

The equity term is the population-weighted coefficient of variation of the gain per jobseeker across markets, where *g*_𝓁_ is the gain per jobseeker in market 𝓁 and $\bar{g}$ its weighted mean. The coefficient of variation is used rather than the Gini or the relative mean deviation because the choice of inequality measure decides the model class: of the standard measures, several are piecewise linear in the decision variable and preserve a linear programme, whereas the coefficient of variation does not [42]. The coefficient of variation is kept because it represents the equity requirement without linearising it, and this nonlinearity is the reason a metaheuristic is used rather than a solver.

(20) minz∈[0,1]L×K×SFALPT(z)=−G(q)Γ+μ CV(q)+P maxC(q)−BB,0

*Γ* is a scale constant, *μ* the weight on equity and *P* the penalty on exceeding the budget. Stripped of the congestion factor and the equity term, what remains is a multiple-choice knapsack problem: exactly one programme intensity is chosen for each market-and-stratum class under a single budget constraint [43]. That linear core is already intractable in the worst case, since the 0–1 knapsack problem is the special case in which each class holds one item [43], and the programme menu adds a setup structure on top of it, because a programme type must be opened in a market at a fixed charge before any place in it can be filled [44]. Exact methods for that linear core reach instances of this size. What they cannot take is the rest of the objective: the congestion factor couples every cell of a market to every other, and the equity term is a coefficient of variation over markets, so the objective is neither linear nor separable. A metaheuristic is used because the model is nonlinear, not because the instances are large.

#### 6.1.2. Instances

Four instances are used, of eight, 14, 24, and 40 local labour markets, giving search dimensions of 128, 224, 384, 640. Table 14 gives their size. Market sizes are lognormal, stratum shares Dirichlet and baseline employment probabilities are drawn so that the spread across markets is wide: if every market were alike there would be no targeting problem. All quantities are drawn once from fixed seeds and are identical across algorithms and runs.

#### 6.1.3. Results

**Figure 14:**
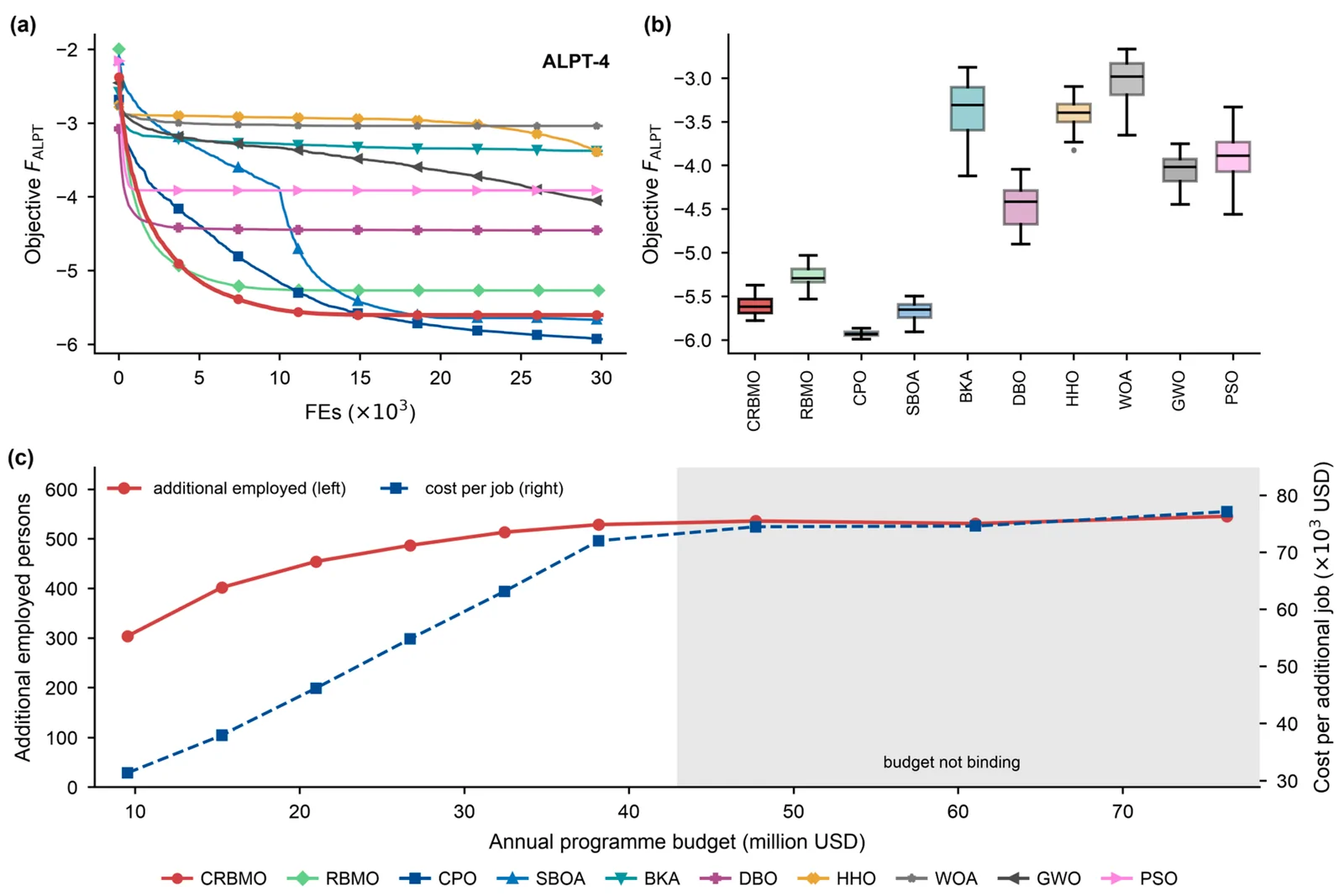
Performance on the ALPT problem: (**a**) convergence (ALPT-4); (**b**) the final objective over 30 runs (ALPT-4); (**c**) the budget curve (ALPT-3).

Table 15 gives the result. CRBMO has the best mean objective on none of the four instances and a mean rank of 2.75, which places it third, behind CPO (1.00) and SBOA (2.25), and ahead of RBMO (4.00). Against RBMO the Wilcoxon rank-sum test at the 0.05 level gives four wins, zero ties and zero losses over the four instances. Figure 14 shows the convergence curves and the distribution of the final objective on the largest instance, and the optimised portfolio against the budget.

**Figure 15:**
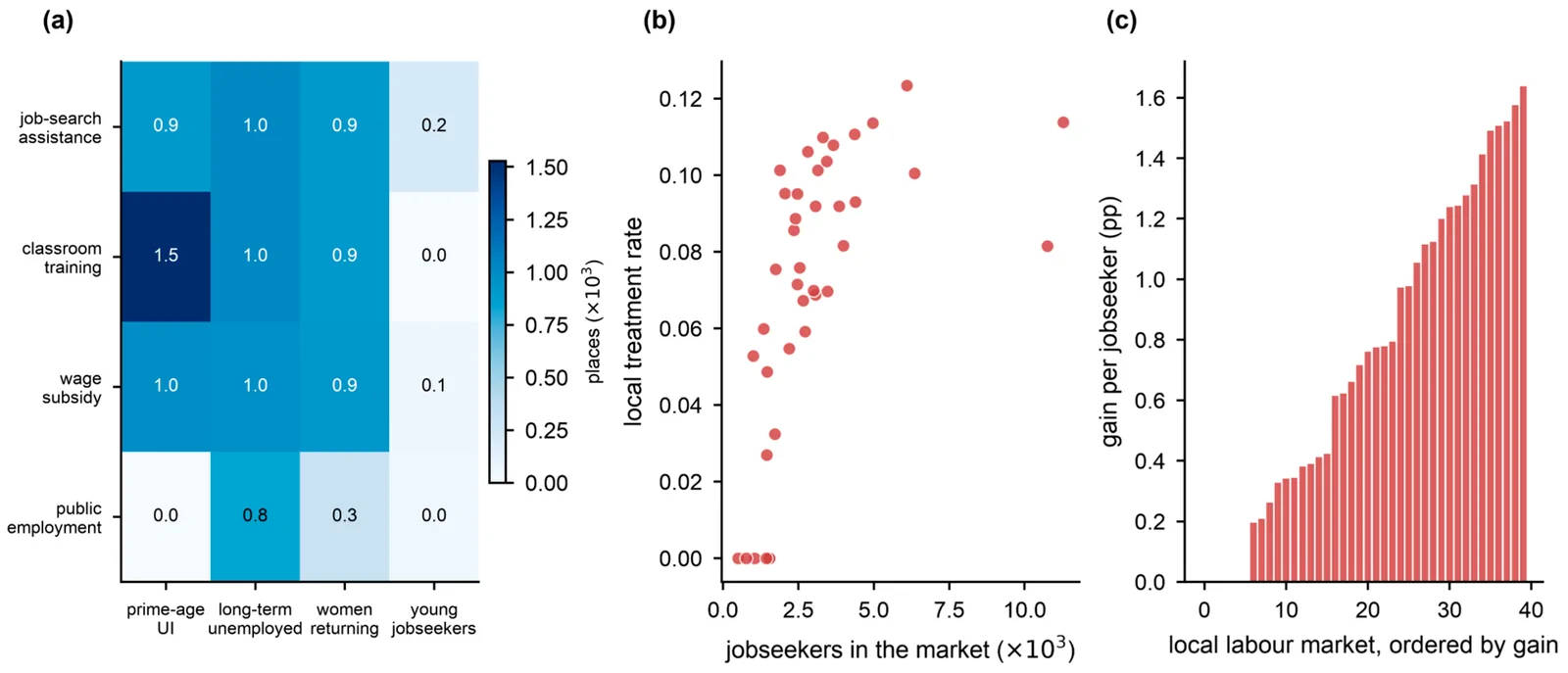
The optimised portfolio on ALPT-4: (**a**) places by programme and stratum; (**b**) the treatment rate against market size; (**c**) gain per jobseeker.

Table 16 reads the best CRBMO solution on each instance back as a policy. Three features recur. The solutions spend 0.935 to 1.000 of the budget. Because Equation (20) contains no reward for leaving money unspent, that alone does not show that the budget binds, and a relaxation test shows that on three of the four instances it does not. Re-solved ten times with a budget 25% larger and the capacity grid held fixed, the median solution spends the extra money only on ALPT-3, where it spends 1.07 of the original budget and the net employment gain rises by 1.3%; on ALPT-1, ALPT-2 and ALPT-4 it spends 0.90 to 0.98 of the original budget, as at the calibrated budget, and the net gain changes by 0.1% to 1.6%, less than its interquartile range over the 30 runs at the calibrated budget. On those three instances money is not what limits the allocation. The mean local treatment rate settles at 0.065 to 0.081. The budget pays for only 50% of the capacity grid, and at the optimum the congestion term removes 8.3% of the direct effect and lock-in a further 36.0%. And the portfolio is concentrated: only 117 of the 160 market programme combinations on the largest instance are opened at all, because the fixed charge makes a thinly spread programme worse than none. In Figure 15b each point is one local labour market. Markets at a treatment rate of zero are of two kinds, and the figure does not distinguish them: six of the 40 markets on this instance are too small for any stratum to reach the viable programme size of 25 places, so no solution can treat them; the rest are markets the optimiser chose to leave untreated. Figure 15 shows the composition of the optimised portfolio.

Figure 14c re-solves one instance at nine budgets spanning $10 to $76 million. The capacity grid is held fixed across the sweep, so what changes between points is the money available and not the set of cells a solution is allowed to open; a sweep that rescaled capacity with the budget would make small markets unreachable at the low end and manufacture a trend in both curves. Up to $38 million the best solutions spend the whole budget, and over that range the cost per additional employed person rises monotonically, from $31.4k to $72.0k. The fixed charges are paid once per opened programme, so the planner opens the cheapest effective cells first and works outward; every additional dollar therefore buys a slightly worse cell than the one before. Beyond that point the budget no longer binds: spending levels off at $40 to $42 million while the budget rises to $76 million, and the employment gain levels off with it. Because Equation (20) does not reward saving money, what is spent in that range buys equity rather than employment, so the cost per job there is not a marginal return, and the range is shaded in the figure. The finding over the binding range does not depend on that feature of the objective: with a cost term 0.1 *C*(**q**)/*B* added to Equation (20), the solutions spend the whole budget up to $38 million and the cost per job rises over that range from $31.2k to $71.9k. That is an empirical finding about the calibrated model, not a theorem, but it is the kind of finding a budget-setting department would want and it is not available from a per-programme evaluation. The mean local treatment rate runs from 0.034 to 0.090 across the sweep.

Equity moves the other way. The population-weighted coefficient of variation of the gain per jobseeker falls overall but not monotonically, from 0.662 at the smallest budget to 0.489 at the largest, because a larger budget lets the planner open programmes in markets that would otherwise be skipped. A planner choosing a budget is therefore choosing a point on two curves that do not have their optima in the same place, and the trade-off between them is the choice, not an artefact of the encoding: the same capacity grid is available at every budget in the sweep.

### 6.2. Occupational Retraining Pathway Design

#### 6.2.1. The Planner’s Problem

The second problem starts from the worker rather than the programme. When an occupation contracts, the policy instrument is a retraining pathway to an occupation that is growing, and the design question is which pathways to open and how many workers to send along each.

Skill distance decides whether a pathway can work at all. Workers who change occupation move to occupations with similar task requirements, and task-specific human capital accounts for a large share of wage growth, so a pathway that spans a wide task distance completes at a lower rate and costs more to deliver [45]. The occupational mobility network is itself a friction rather than a neutral substrate: steady-state unemployment is 5.3% on the empirical network against 4.1% on a complete one, a 29% increase, and under an automation shock the peak rises further [46]. Doing nothing is expensive and the expense is permanent, not transitory: workers displaced in recession mass layoffs earn about 15% less per year for at least fifteen years [47]. Doing the wrong thing is also expensive. Displacement raises the probability of an occupational change elevenfold, and averaged over the fifteen years that follow, switchers earn 16.5% less than before they were displaced; the gap is 22.4% for those who move down the skill distribution against 8.9% for those who move up [48]. It is that asymmetry, not the average, that a pathway designer acts on. Where the source occupation sits in skill space matters too: workers laid off from jobs above the median in local skill remoteness lose substantially more over the following four years [49]. Against all of this, retraining has a measured return: in randomised evaluations of sector-focused training, earnings rose by 12% to 34% after training, and the earnings gains persisted after the employment gains had faded [41].

Cast as an optimisation problem this is a fixed-charge transportation problem: a linear cost per unit of flow plus a fixed charge triggered by any positive flow on an arc [50]. The problem is NP-hard even when the network is reduced to a single sink served by capacitated sources [51], and that case is a restriction of the bipartite network used here. Exact methods for the linear problem are strong and still improving: a recent reformulation solves the single-sink case several orders of magnitude faster than earlier exact methods [51]. What puts the problem below out of the reach of such methods is its objective. Completion probabilities scale each flow, a target that is oversubscribed absorbs its arrivals pro rata, and the equity term is a coefficient of variation, so the objective is neither linear nor separable over arcs. A metaheuristic is used because the model is nonlinear, not because the instances are large.

Call this the occupational retraining pathway problem, ORP. A national agency must decide which retraining pathways to open between *I* source occupations, where workers are being displaced, and *J* target occupations, where vacancies are unfilled, and how many workers to send along each. Write *f_ij_* for the number of workers directed from source *i* to target *j*. Each occupation is a point in a six-dimensional task space, and the decision variable is again a vector of random keys, decoded against a per-arc capacity, rounded down, with any arc below a viable cohort size set to zero and any source row scaled back if it would send more workers than it has.

(21) $${𝛿}_{\mathrm{ij}}=\frac{{\left\|t_{i}-t_{j}\right\|}_{2}}{\sqrt{T}\max_{i’,j’}{\left\|t_{i’}-t_{j’}\right\|}_{2}/\sqrt{T}},\;\;\;p_{\mathrm{ij}}=\exp\left(-{𝛿}_{\mathrm{ij}}/{𝛿}_{0}\right)$$

**t***_i_* is the task vector of occupation *i* and *𝛿_ij_* the normalised task distance between a source and a target. The probability that a worker directed along an arc completes the transition falls exponentially in that distance, with *𝛿*_0_ fixed by bisection so that the mean completion probability over the distance matrix equals the observed occupational-switching share of displaced workers. This is the mechanism that makes the problem a design problem rather than an accounting exercise: the cheapest arcs are the short ones, the vacancies are not where the displaced workers are, and the planner must trade the two against each other.

(22) $$V(f)=\frac{1-(1+r{)}^{-H}}{r}\sum_{i,j}p_{\mathrm{ij}}{\tilde{f}}_{\mathrm{ij}}\left[w_{j}\left(1-\pi_{\mathrm{ij}}\right)\left(1+\gamma \right)-w_{i}\left(1-\pi_{0}\right)\right]$$

The value of a pathway is the discounted earnings difference over a horizon *H* at discount rate *r*, between completing the transition and not retraining at all. *w_i_* and *w_j_* are the source and target wages, *γ* the measured return to retraining, *π_ij_* the switching penalty—larger when the move is downskilling than when it is upskilling—and *π*_0_ the permanent earnings loss of a displaced worker who does not retrain. Both penalties are applied to the wage the worker ends up on, which is the modelling choice the source estimates support least directly: they are reported relative to the worker’s own pre-displacement earnings, and are used here as multiplicative discounts on the destination wage. ${\tilde{f}}_{\mathrm{ij}}$ is the flow after two feasibility limits: a source occupation cannot send more workers than it has, and a target cannot absorb more than its vacancies, in which case completions are squeezed pro rata. Both limits are slack at the budgets used here—at full capacity the most heavily subscribed target is filled to 0.20 of its vacancies—so the binding constraint on these instances is money, not absorption capacity.

(23) $$C(f)=\sum_{i,j}\left(c_{0}+c_{1}{𝛿}_{\mathrm{ij}}\right)f_{\mathrm{ij}}+\Phi \sum_{i,j}1\left[f_{\mathrm{ij}}\geq f^{\min}\right]$$

*c*_0_ is the base cost of retraining one worker, *c*_1_ the additional cost per unit of task distance and *Φ* the fixed charge for opening a pathway at all—curriculum development, accreditation and provider contracts, which are incurred whether one worker or a thousand walk through the door.

(24) minz∈[0,1]I×JFORP(z)=−V(f)Γ+μ CV(f)+P maxC(f)−BB,0

with *Γ* a scale constant, *P* the budget penalty and *μ* the weight on CV(**f**), which here is the coefficient of variation across source occupations of the share of that occupation’s displaced workers who are placed on a pathway—a coverage measure, not the per-jobseeker gain of Equation (19). The combination of a per-unit flow cost and a fixed charge per opened arc is the structure that makes the problem hard; the completion probabilities, the absorption limit and the equity term make it nonlinear on top of that.

#### 6.2.2. Instances

Four instances are used, from eight sources and 10 targets to 25 and 34, giving search dimensions of 80, 192, 432, 850. Table 17 gives their size. The budget is deliberately tight: it would serve about 30% of the displaced stock at the base cost and less than that once distance and fixed charges are paid, so the interesting question is which pathways to open rather than how many workers to move.

#### 6.2.3. Results

**Figure 16:**
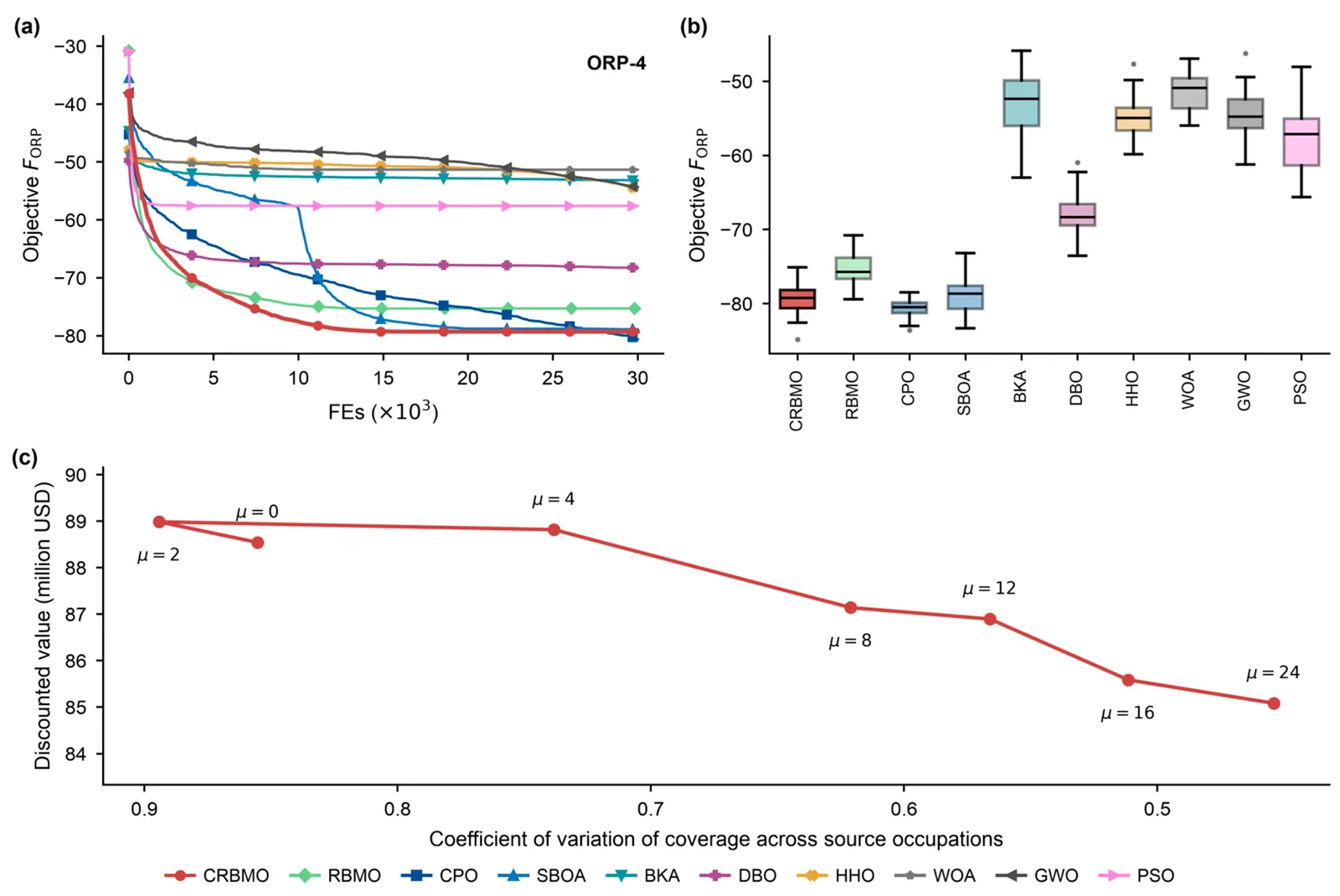
Performance on the ORP problem: (**a**) convergence (ORP-4); (**b**) the final objective over 30 runs (ORP-4); (**c**) the equity-value frontier (ORP-3).

Table 18 gives the result. CRBMO has the best mean objective on one of the four instances and a mean rank of 2.25, which places it joint second with SBOA, behind CPO (1.75), and ahead of RBMO (4.00). Against RBMO the Wilcoxon rank-sum test at the 0.05 level gives four wins, zero ties and zero losses over the four instances. Figure 16 shows the convergence curves, the distribution of the final objective and the equity-value frontier.

**Figure 17:**
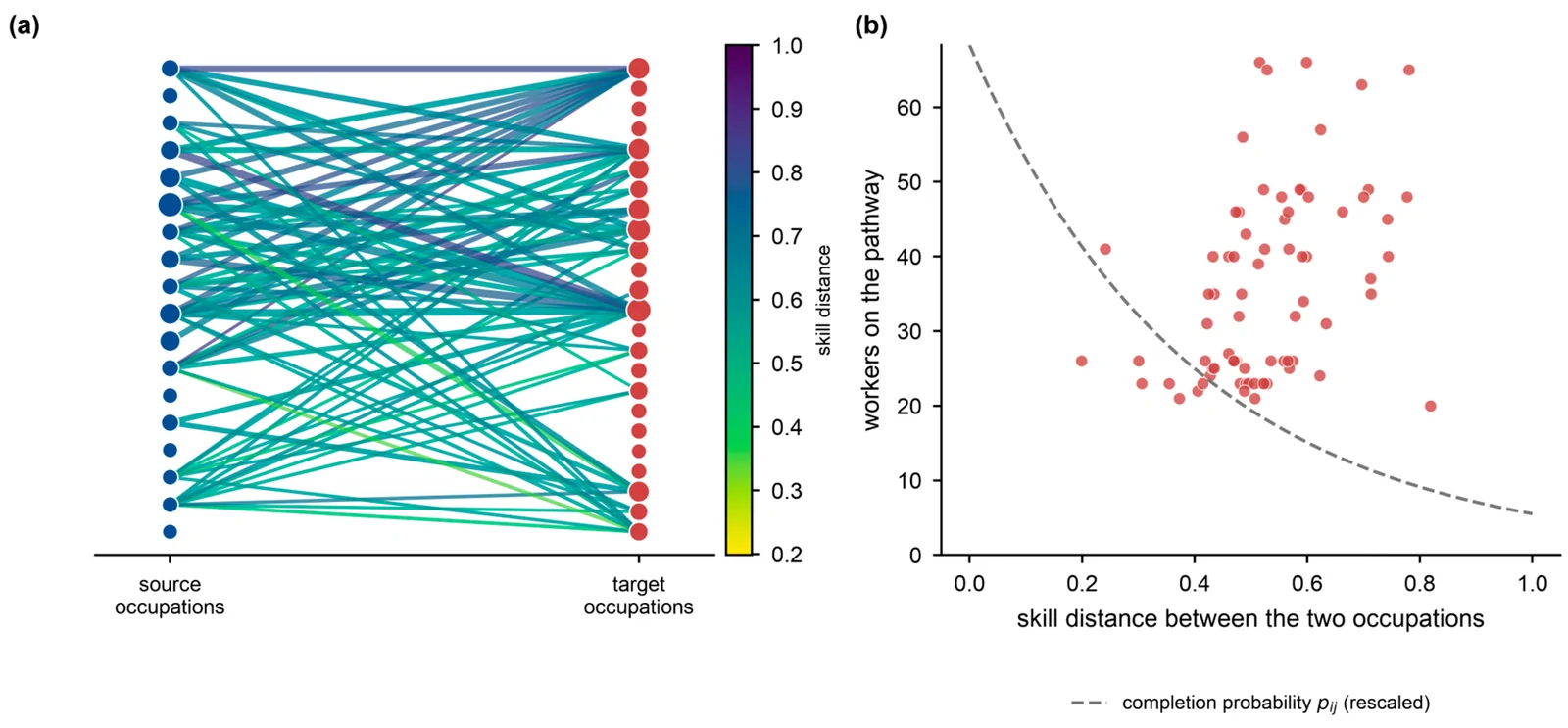
The optimised retraining programme on ORP-3: (**a**) the opened pathways; (**b**) workers on a pathway against the skill distance it spans. In (**a**), blue source circles increase in area with the displaced-worker stock and red target circles with the vacancies; line width increases with the workers allocated to a pathway, and colour gives its skill distance.

Table 19 reads the best CRBMO solution back as a programme. The pattern is consistent across instances and is not one that was designed in. The optimiser opens 109 of the 850 possible pathways on the largest instance, spends 1.000 to 1.001 of the budget (a budget 25% larger is spent in full and raises the discounted value of the solutions by 7.6% to 18.6%, so on this problem money binds), and serves 9.8% to 12.8% of the displaced stock. The upskilling share of expected completions runs from 20% to 71% across the four instances. The objective function contains no preference for either direction; what it contains is the measured asymmetry in the switching penalty, which is more than twice as large for a downskilling move as for an upskilling one. Where the share is high the planner is buying that asymmetry; where it is low, the short-distance pathways on offer lead to target wages high enough to pay the penalty. Figure 17a draws the opened pathways, with the width of each line increasing with the flow along it and its colour giving the skill distance it spans; the node areas increase with the displaced stock on the left and the unfilled vacancies on the right.

The equity weight *μ* is a policy choice, not a model parameter, so the instance was re-solved across seven values of it. Raising *μ* from 0 to 12 cuts the coefficient of variation of coverage from 0.855 to 0.566, a reduction of 34%, at a cost of 1.9% of the discounted value. Reaching 0.454, half the original inequality, costs 3.9%. The number of opened pathways is almost unchanged across the frontier (72 to 76), so the equity term is redistributing workers among a stable set of pathways rather than buying new ones. For a planner the frontier is therefore more informative than any single optimum, because it states what each reduction in inequality costs.

### 6.3. What the Applications Say About the Ranking

The two applications do not reproduce the ordering of Section 5.4. CPO is first on both, with mean ranks of 1.00 and 1.75; CRBMO is third on the programme problem, behind CPO and SBOA, and joint second with SBOA on the retraining problem. On the suite the ordering runs the other way: CRBMO leads at 1.72 against 3.32 for CPO. The applications are not small: their decision vectors have 128 to 640 components on the programme problem and 80 to 850 on the retraining problem, seven of the eight at or above the largest dimension of the suite, where CRBMO’s advantage at 30,000 evaluations is largest. CPO’s lead here therefore cannot be explained by dimension: the dimensional pattern of Section 5.4 does not carry over to these problems, and what separates the two settings is the structure of the encoding.

Two features of the application encodings are absent from CEC2017. The first is plateaus. The decision vector is a set of random keys that a deterministic decoder maps to a policy [52], and such encodings are many-to-one [53]; here the decoder also rounds places or workers down to integers and closes every cell below a viable size, so whole regions of key space decode to the same policy. The plateaus are measurable: around CRBMO’s final solutions, a perturbation of 2% of the coordinates at scale 10^−3^ leaves the objective exactly unchanged in 41% to 87% of trials. They do not, however, explain the ranking. CPO keeps a candidate of equal value, RBMO’s released code does the same, and CRBMO, following Equation (5), does not; yet a version of CRBMO that keeps ties is statistically indistinguishable from CRBMO on eight of the eight instances. The second feature is the kind of move that succeeds. Each coordinate of an application encodes one cell (a market, programme and stratum, or one pathway) under a single shared budget, so a good solution is improved by changing a few cells and moving money between them. Around CRBMO’s final solutions, perturbing 2% of the coordinates improves the objective in up to 11% of trials, whereas perturbing every coordinate by a step of the same length does so in at most 1.2%; on the rotated CEC2017 functions neither kind of move succeeds in more than 1% of trials. RBMO and CRBMO move every coordinate at once, through the population difference and the Gaussian factor of Equation (4) and Equation (7), whereas CPO’s second and third defences change a random subset of coordinates through a binary mask. As a diagnostic, and not as a proposal, CRBMO was re-run with its attacking candidates restricted to a random tenth of their coordinates. The restricted version is better than CRBMO on eight of the eight instances by the Wilcoxon rank-sum test, and against CPO it wins eight, ties none and loses none. The benchmark improvement of this paper concerns the scale of a dense step; what the applications reward in the end game is the sparsity of the move, a property the collapse repair neither provides nor was designed to provide.

Two things temper how much either ordering will bear. The application ranks rest on four instances per problem, too few for a rank test to carry weight; they are an ordering, not a tested result. And the gaps are small: CPO is ahead of CRBMO by 3.7% to 6.0% of the objective on the programme instances and 1.7% to 3.3% on the retraining instances where it leads, while CRBMO is ahead of RBMO by 5.0% to 10.4% and 4.6% to 7.3% on the same instances. The comparison this paper is about does carry over: CRBMO beats the algorithm it repairs on every instance of both problems, 4/0/0 and 4/0/0 by Wilcoxon. What these applications establish is that the repair survives a change of problem class. They do not establish that CRBMO is the instrument to reach for on problems of this kind; on the evidence assembled here, an algorithm that makes sparse moves, as CPO does, is better suited to them.

## 7. Discussion

The main result of this paper is not the rank table but the identification of a specific, measurable mechanism in RBMO, together with the finding that repairing it produced, at the 30,000-evaluation budget, the improvement the mechanism predicts. The prediction was dimensional: because a larger box takes longer to cover, the collapse costs more where there is more left to do, so a repair aimed at the collapse should pay increasingly with dimension. The measured gain against RBMO is 1.03 rank units at *D* = 10, 1.24 rank units at *D* = 30, 1.31 rank units at *D* = 50 and 2.14 rank units at *D* = 100. That is the predicted ordering, but it is not an independent test of the prediction: *τ* and *ϕ*_min_ were set by the sweeps of Section 5.2 on the same suite at *D* = 30 and *D* = 100, so the chosen values have seen two of the four dimensions on which the ordering is judged. The two dimensions the sweeps did not use, *D* = 10 and *D* = 50, fall into the predicted order as well, but they are not untouched by the tuning either: the chosen *ϕ*_min_ trades rank at *D* = 30 for rank at *D* = 100 (Table 5), and the allocation it controls costs 0.77 rank units at *D* = 10 but next to nothing at *D* = 50 (Section 5.3), which steepens the gradient between the two. At the standard CEC2017 budget of Section 5.7, at which no parameter was tuned, the prediction holds only in part: the gain over RBMO is 0.00, 0.48, 0.62 and 0.45 rank units from *D* = 10 to *D* = 100, growing up to *D* = 50 but not beyond, and Holm’s procedure does not separate the two in any dimension.

The diagnosis is not specific to RBMO. Two ingredients produce it: a step whose scale is a statistic of the population, and an operator that moves accepted individuals onto a single incumbent. Any algorithm with both has the same loop available to it, and the pairing is common—it is what centroid-based and best-anchored update rules look like when they are combined. RBMO’s own operators have already been transplanted into another optimiser, carrying the coupling with them [54]. The transferable result is the measurement rather than the specific repair: instrument the population spread and the acceptance rate against the consumed budget, and the presence or absence of the loop is visible in one figure. The three repairs proposed here follow from it directly, and they are cheap.

The benchmark evidence has three limits. First, the separation reported above is a statement about this suite and these budgets, not about the algorithms in general; at 30,000 evaluations CMA-ES ranks ahead of CRBMO at *D* = 10, *D* = 30 and *D* = 50 and L-SHADE and jSO at *D* = 10 and *D* = 30 (Section 5.8); all three rank ahead of it in every dimension at the standard CEC2017 budget (Section 5.7); and CRBMO’s lead over them at *D* = 100 therefore holds only at the shorter budget. At the standard budget CRBMO also ends far behind RBMO on the ill-conditioned F1 in every dimension, and the instrumentation at *D* = 30 shows the search stalling there despite the radius control (Section 5.7). Second, the repair costs something at the lowest dimension, where CRBMO ranks 2.28 against 1.79 for CPO, the best of the ten swarm algorithms at *D* = 10; jSO, L-SHADE and CMA-ES rank ahead of CRBMO there as well (Section 5.8). This is consistent with the diagnosis rather than an exception to it: the collapse does not bite within the budget at low dimension, so the machinery that prevents it is overhead there. Third, CEC2017 is a suite of shifted and rotated synthetic functions, and a suite is not a population of problems, which is why Section 6 tests the algorithm on two problems outside it.

The applications have their own limits, and they are the limits of the evidence they are built from. The effect sizes, lock-in profiles, displacement slope, skill-distance gradient and returns to retraining are point estimates transported from the settings in which they were estimated, mostly European and North American, mostly from the two decades before 2020. They are used as a calibration, not as a forecast. The instances are synthetic in their market sizes and task vectors, which are drawn from fixed seeds rather than taken from an administrative register; what is not synthetic is the structure of the objective function or the magnitudes of its coefficients. A planner with access to real registers would replace the draws and keep the model. Section 6 should therefore be read as evidence about the optimiser and about the shape of the design problem, not as a recommendation for any particular jurisdiction.

With that said, two features of the optimised solutions are worth a policy reader’s attention because neither was built into the model. First, the cost per additional employed person and the inequality of the gain across local markets move in opposite directions as the budget rises, so a planner choosing a budget is choosing a point on a trade-off rather than an optimum. Second, on the retraining problem a third of the inequality in coverage can be removed for 1.9% of the discounted value, because the equity term redistributes workers among a stable set of pathways rather than buying new ones. Both are statements about the calibrated model, and both are the kind of statement that an ex post evaluation body of literature cannot make, because they concern a counterfactual allocation that was never run.

## 8. Conclusions

This paper measured what limits the red-billed blue magpie optimizer and repaired it. The measurement identified a feedback loop between the scale of the step and the spread of the population, and the repair—a dual-anchor attack, a collapse-triggered radius control and an adaptive foraging allocation—adds no new search operator and no new metaphor. The resulting algorithm was evaluated on the full CEC2017 suite against nine swarm algorithms, three published RBMO variants and three evolutionary algorithms at 30,000 evaluations, and against RBMO and the three evolutionary algorithms at the standard CEC2017 budget, and it was applied to two design problems in employment policy.

1. Instrumenting RBMO shows that its population spread falls below 10^−12^ within the evaluation budget on all five instrumented functions, and that on four of them the error stops changing in its fourth significant figure over the last 28% to 58% of the run. At ten times the budget the stagnant share stays the same or grows on four of the five functions, so the loss scales with the budget rather than being amortised by it.
2. At 30,000 evaluations and against the nine swarm algorithms, CRBMO attains the best Friedman mean rank at *D* = 30, *D* = 50, *D* = 100 and 1.72 overall, against 3.16 for RBMO, and its advantage over RBMO grows with dimension, as the diagnosis predicts, although the parameters were set by sweeps on two of these dimensions. At the standard CEC2017 budget the gain grows only up to *D* = 50 and is not significant in any dimension (Section 5.7).
3. In every dimension CRBMO outranks the three published RBMO variants that could be run. Against the evolutionary algorithms, at 30,000 evaluations CMA-ES ranks ahead of CRBMO at *D* = 10, *D* = 30 and *D* = 50 and L-SHADE and jSO at *D* = 10 and *D* = 30, while CRBMO has the best mean rank at *D* = 100; at the standard CEC2017 budget all three rank ahead of it in every dimension, and it ranks fourth of five (Section 5.7 and Section 5.8).
4. A complete factorial ablation attributes the gain to the dual-anchor attack and the radius control, puts the foraging allocation at −0.069 rank units against −2.526 and −1.957 for the other two, and shows all three pairwise interactions to be positive: the strategies overlap rather than compound, which is what three repairs to one loop should do and what a sequence of one-at-a-time removals could not have shown. The foraging allocation costs rank up to *D* = 30 and pays at *D* = 100, which is why it is retained for high-dimensional problems.
5. On the two employment-policy problems the suite ordering does not carry over. CRBMO beats RBMO on every instance of both, 4/0/0 and 4/0/0 by Wilcoxon and with no parameter retuning, but CPO ranks first on both at 1.00 and 1.75 against 2.75 and 2.25 for CRBMO. The repair transfers to a new problem class; the ranking does not.
6. The optimised policies are themselves informative: cost-effectiveness and equity move in opposite directions as the programme budget rises, and on the retraining problem a third of the inequality in coverage can be bought for 1.9% of the discounted value.

Three directions follow. The diagnosis should be applied as a test rather than a result: any optimiser whose step scale is a population statistic and whose exploiting operator anchors on a single incumbent can be screened for the same loop with the same instrumentation, and the screening costs one run. The radius control is deliberately the smallest possible controller—one scalar under one classical rule—and the natural next question is what a richer one would buy, and at what cost in being able to attribute the gain to anything. Finally, the two policy models are calibrated on published estimates rather than estimated on administrative data; replacing the synthetic instances with a national register would turn the budget curve and the equity-value frontier from properties of a calibrated model into advice.

## Figures and Tables

**Table 1:** Published improvements to RBMO: the defect each paper states and the operators it adds.

Variant	Year	Stated Defect	Operators Added
IRBMO [9]	2025	premature convergence and degradation on high-dimensional constrained problems, from over-reliance on population mean vectors	Logistic-Tent chaotic map; dynamic balance factor; Jacobi-curve and Levy perturbation
MRBMO [11]	2025	trapping in local optima, slow convergence	good-nodes-set initialisation; enhanced search; siege-style attack; lens-imaging opposition-based learning
AR-RBMO [17]	2025	restricted global exploration, premature convergence to local optima	attraction–repulsion mechanism; best-solution attraction; escape strategy; feedback-driven balancing
CLD-RBMO [10]	2026	declining population diversity and low convergence efficiency in the middle and later stages	Logistic chaotic map; Levy hierarchical perturbation; Cauchy–Gauss mutation; stochastic differential mutation
ERBMO [18]	2026	weak population diversity, premature convergence, exploration–exploitation imbalance	diversity-adaptive weight updating; periodic pattern search; evolutionary probabilistic combinatorial mutation

The variant names are the acronyms the source papers give their own algorithms. Defects are given in the source papers’ own terms. Chaotic initialisation, opposition-based learning, Levy flights and Cauchy or differential mutation are general-purpose devices that can be attached to any population-based optimiser. IRBMO, the improved red-billed blue magpie algorithm of [9]; MRBMO, the enhanced red-billed blue magpie optimization algorithm of Lu et al. [11]; AR-RBMO, the enhanced red-billed blue magpie optimizer with attraction–repulsion and dynamic balancing strategies [17]; CLD-RBMO, the multi-strategy improved red-billed blue magpie optimization algorithm of Ni et al. [10]; ERBMO, the enhanced red-billed blue magpie optimizer of Wang et al. [18]. The labels follow the source papers.

**Table 2:** The mean rank of RBMO and the three strongest comparison algorithms within each CEC2017 function group.

Function Group	RBMO	SBOA	CPO	PSO
unimodal	1.50	2.50	2.13	3.88
simple multimodal	1.93	3.00	2.39	2.68
hybrid	2.08	2.18	2.08	3.68
composition	2.60	1.68	2.08	3.65

The ranks are taken within these four algorithms only, over the functions of each group (2 unimodal, 7 simple multimodal, 10 hybrid and 10 composition functions) in each of the four dimensions, on the mean error of 30 independent runs. CRBMO is not included, so that the diagnosis does not rest on the algorithm it motivates. SBOA, secretary bird optimisation algorithm; CPO, crested porcupine optimizer; PSO, particle swarm optimisation.

**Table 3:** The parameter settings of CRBMO and the comparison algorithms.

**Algorithm**	**Year**	Parameter Settings
CRBMO	2026	dual anchor with probability 1/2; collapse threshold *τ* = 10^−3^; one-fifth success control with *c* = 0.94, p^*^ = 0.20 and *ε* = 10^−3^; scouting fraction *ϕ*(*t*) = 1 − *t*/*T* (*ϕ*_min_ = 0)
RBMO	2024	sequential update of the released code; small group of *p*∼𝒰{2,…,5} and cluster of *q*∼𝒰{10,…,*n*}, drawn without replacement from the whole population; group split *ϵ* = 0.5; *CF* = (1 − *t*/*T*)^2*t*/*T*^
CPO	2024	cyclic population reduction with *N*_min_ = 0.8*n* = 24 and *T*_cycle_ = 2; convergence rate *α* = 0.2; trade-off *T_f_* = 0.8
SBOA	2024	hunting stages split at *T*/3 and 2*T*/3; *CF* = (1 − *t*/*T*)^2*t*/*T*^; Levy exponent *β* = 1.5 with *RL* = 0.5 Levy; escape threshold *r* = 0.5; *K* ∈ {1,2}
BKA	2024	*p* = 0.9; attack step $n=0.05e^{-2(t/T{)}^{2}}$; migration coefficient *m* = 2sin (*r* + *π*/2); standard Cauchy step
DBO	2023	roles 6 ball-rolling, 6 brood, 7 small and 11 thief beetles; *k* = 0.1; *b* = 0.3; *S* = 1; dancing branch with probability 0.1; *α* = ±1 with probabilities 0.9 and 0.1; *R* = 1 − *t*/*T*
HHO	2019	*E*_0_∼𝒰(−1,1) per hawk and iteration, *E* = 2*E*_0_(1 − *t*/*T*); jump strength *J* = 2(1 − *r*); Levy exponent *β* = 1.5 with scale 0.01
WOA	2016	*a* decreasing linearly from 2 to 0; *a*_2_ from −1 to −2; spiral constant *b* = 1
GWO	2014	*a* decreasing linearly from 2 to 0; *A* = 2*ar*_1_ − *a* and *C* = 2*r*_2_ redrawn per wolf, dimension and leader
PSO	1995	inertia weight *w* decreasing linearly from 0.9 to 0.4; *c*_1_ = *c*_2_ = 2.0; *V*_max_ = 0.2(*ub* − *lb*)

The values are those of the source publications, except the parameters of CRBMO (Section 5.2) and the inertia schedule and velocity clamp of PSO (Section 5.1); the population size and the evaluation budget are standardised across all algorithms. CRBMO, collapse-resistant red-billed blue magpie optimizer; RBMO, red-billed blue magpie optimizer; DBO, dung beetle optimizer; SBOA, secretary bird optimisation algorithm; BKA, black-winged kite algorithm; CPO, crested porcupine optimizer; PSO, particle swarm optimisation; GWO, grey wolf optimizer; WOA, whale optimisation algorithm; HHO, Harris hawks optimisation.

**Table 4:** The Friedman mean ranks of CRBMO for four values of the collapse threshold *τ*.

Variant	D = 30	D = 100	Overall
*τ* = 10^−2^	2.52	1.66	2.09
*τ* = 10^−3^	**2.10**	**1.79**	**1.95**
*τ* = 10^−4^	2.28	2.90	2.59
*τ* = 10^−5^	3.10	3.66	3.38

The ranks are over the 29 CEC2017 functions in each dimension column and over the 58 function–dimension pairs in the Overall column, 30 runs each. The best overall rank is in bold. Friedman *χ*^2^ = 43.72, *p* < 0.001.

**Table 5:** The Friedman mean ranks of CRBMO for six values of the scouting floor *ϕ*_min_.

Variant	D = 30	D = 100	Overall
*ϕ*_min_ = 0.00	**3.72**	**2.45**	**3.09**
*ϕ*_min_ = 0.20	3.45	3.03	3.24
*ϕ*_min_ = 0.40	3.45	2.90	3.17
*ϕ*_min_ = 0.60	3.34	3.62	3.48
*ϕ*_min_ = 0.80	3.79	4.55	4.17
*ϕ*_min_ = 1.00	3.24	4.45	3.84

The ranks are over the 29 CEC2017 functions in each dimension column and over the 58 function–dimension pairs in the Overall column, 30 runs each. The best overall rank is in bold. Friedman *χ*^2^ = 15.19, *p* = 0.010.

**Table 6:** The Friedman mean ranks of CRBMO for five values of the isotropic constant *ε*.

Variant	D = 30	D = 100	Overall	Rank
*ε* = 0	3.21	4.00	3.60	5
*ε* = 10^−5^	2.38	3.10	2.74	2
*ε* = 10^−4^	**2.79**	**2.45**	**2.62**	**1**
*ε* = 10^−3^	3.10	2.76	2.93	3
*ε* = 10^−2^	3.52	2.69	3.10	4

The ranks are over the 29 CEC2017 functions in each dimension column and over the 58 function–dimension pairs in the Overall column, 30 runs each. The best overall rank is in bold. Friedman *χ*^2^ = 13.70, *p* = 0.008.

**Table 7:** The eight variants of the ablation study.

Variant	DAA	CRC	AFA	Description
V1	—	—	—	vectorised RBMO, the common base of CRBMO (Section 5.1)
V2	yes	—	—	V1 plus DAA
V3	—	yes	—	V1 plus CRC
V4	—	—	yes	V1 plus AFA
V5	yes	yes	—	V1 plus DAA and CRC
V6	yes	—	yes	V1 plus DAA and AFA
V7	—	yes	yes	V1 plus CRC and AFA
V8	yes	yes	yes	the full algorithm of Section 4

DAA is the dual-anchor attack of Section 4.1, CRC the collapse-triggered radius control of Section 4.2 and AFA the adaptive foraging allocation of Section 4.3.

**Table 8:** The Friedman mean ranks of the eight ablation variants on the CEC2017 suite.

Variant	D = 30	D = 100	Overall
V1	6.59	7.21	6.90
V2	3.62	4.86	4.24
V3	5.24	4.34	4.79
V4	6.83	6.79	6.81
**V5**	**1.83**	**2.59**	**2.21**
V6	3.59	4.34	3.97
V7	5.41	3.69	4.55
V8	2.90	2.17	2.53

The **r**anks are over the 29 CEC2017 functions in each dimension column and over the 58 function–dimension pairs in the Overall column, 30 runs each. The best overall rank is in bold. Friedman *χ*^2^ = 199.56, *p* < 0.001.

**Table 9:** The Friedman mean ranks of CRBMO and the comparison algorithms on the CEC2017 suite.

Algorithm	D = 10	D = 30	D = 50	D = 100	Overall	Final Rank
**CRBMO**	**2.28**	**1.62**	**1.76**	**1.24**	**1.72**	**1**
RBMO	3.31	2.86	3.07	3.38	3.16	2
CPO	1.79	3.31	3.90	4.28	3.32	4
SBOA	3.55	3.28	2.90	2.97	3.17	3
BKA	5.97	6.79	6.79	6.79	6.59	7
DBO	6.76	6.86	6.93	6.83	6.84	8
HHO	9.48	9.59	9.66	9.48	9.55	10
WOA	9.28	9.41	9.34	9.34	9.34	9
GWO	6.86	6.24	5.52	5.03	5.91	6
PSO	5.72	5.03	5.14	5.66	5.39	5

The **r**anks are over the 29 CEC2017 functions in each dimension column and over the 116 function–dimension pairs in the Overall column, 30 runs each. The best algorithm is in bold.

**Table 10:** The Friedman mean ranks of CRBMO and the comparison algorithms within the four CEC2017 function groups.

Algorithm	Unimodal	Simple Multimodal	Hybrid	Composition
CRBMO	**1.63**	**1.61**	**1.73**	**1.83**
RBMO	2.00	3.00	2.90	3.75
CPO	3.75	3.75	3.05	3.20
SBOA	3.75	4.11	3.18	2.40
BKA	5.75	6.89	5.90	7.23
DBO	6.38	7.36	7.08	6.35
HHO	9.00	9.18	9.58	9.90
WOA	9.50	9.79	9.25	9.10
GWO	6.75	5.29	6.53	5.58
PSO	6.50	4.04	5.83	5.68

The best mean rank in each function group is in bold.

**Table 11:** The Wilcoxon rank-sum test results of CRBMO versus the comparison algorithms on the CEC2017 suite (*α* = 0.05).

CRBMO vs.	D = 10	D = 30	D = 50	D = 100	Total
RBMO	10/13/6	18/10/1	20/7/2	25/4/0	**73/34/9**
CPO	8/7/14	17/9/3	22/4/3	26/2/1	**73/22/21**
SBOA	17/10/2	19/9/1	19/9/1	22/7/0	**77/35/4**
BKA	27/2/0	29/0/0	29/0/0	28/1/0	**113/3/0**
DBO	28/1/0	29/0/0	29/0/0	29/0/0	**115/1/0**
HHO	29/0/0	29/0/0	29/0/0	29/0/0	**116/0/0**
WOA	29/0/0	29/0/0	29/0/0	29/0/0	**116/0/0**
GWO	28/1/0	27/2/0	25/4/0	25/4/0	**105/11/0**
PSO	24/4/1	25/4/0	24/5/0	27/2/0	**100/15/1**
**All**					**888/121/35**

The entries give the wins/ties/losses of CRBMO against the algorithm in the row at the 0.05 level, over the 29 functions in each dimension column and over the 116 function–dimension pairs in the Total column. Bold entries in the Total column and the All row mark totals pooled over all dimensions; bold does not indicate significance or the best algorithm.

**Table 12:** The Friedman mean ranks of CRBMO, RBMO, L-SHADE, jSO and CMA-ES at the CEC2017 standard budget of 10,000*D* evaluations.

Algorithm	D = 10	D = 30	D = 50	D = 100	Overall	W/T/L
CRBMO	4.00	4.03	4.14	4.21	4.09	—
RBMO	4.00	4.52	4.76	4.66	4.48	28/78/10
L-SHADE	2.05	1.95	1.81	1.93	1.94	1/12/103
jSO	**1.57**	**1.43**	**1.53**	**1.69**	**1.56**	1/6/109
CMA-ES	3.38	3.07	2.76	2.52	2.93	11/33/72

The ranks are over the 29 CEC2017 functions in each dimension column and over the 116 function–dimension pairs in the Overall column, 10 runs each at 10,000*D* evaluations. The best mean rank in each column is in bold. W/T/L gives the wins, ties and losses of CRBMO against the algorithm in the row by the Wilcoxon rank-sum test at the 0.05 level, over the 116 function–dimension pairs.

**Table 13:** The Friedman mean ranks of CRBMO, RBMO, three published RBMO variants, L-SHADE, jSO and CMA-ES on the CEC2017 suite (30,000 evaluations).

Algorithm	D = 10	D = 30	D = 50	D = 100	Overall	W/T/L
CRBMO	4.24	3.66	3.31	**2.28**	3.37	—
RBMO	5.21	5.21	4.90	4.69	5.00	73/34/9
CLD-RBMO	5.10	5.07	5.07	5.34	5.15	82/27/7
MRBMO	7.69	8.00	7.90	7.76	7.84	115/1/0
ERBMO	5.76	5.10	3.97	3.21	4.51	65/32/19
L-SHADE	2.19	3.55	4.97	5.52	4.06	53/13/50
jSO	**1.64**	2.97	3.66	4.34	3.15	44/13/59
CMA-ES	4.17	**2.45**	**2.24**	2.86	**2.93**	29/16/71

The ranks are over the 29 CEC2017 functions in each dimension column and over the 116 function–dimension pairs in the Overall column, 30 runs each. The best mean rank in each column is in bold. W/T/L gives the wins, ties and losses of CRBMO against the algorithm in the row by the Wilcoxon rank-sum test at the 0.05 level, over the 116 function–dimension pairs.

**Table 14:** The four active labour-market programme instances.

Instance	Markets *L*	Dimension *D*	Jobseekers	Budget ($M)	Places Affordable
ALPT-1	8	128	24,456	10.8	2110
ALPT-2	14	224	40,814	18.0	3521
ALPT-3	24	384	86,715	38.2	7481
ALPT-4	40	640	124,336	54.7	10,727

Every instance has four programme types and four strata, so *D* = 16*L*. Market sizes, stratum shares and baseline employment probabilities are drawn from fixed seeds and are identical for every algorithm. The budget is set at 11% of the jobseeker stock times a mean place cost, and the last column gives the places that budget would buy at the unweighted mean programme cost.

**Table 15:** The objective values and mean ranks of CRBMO and the comparison algorithms on the four active labour-market programme instances.

Instance	Metric	CRBMO	RBMO	CPO	SBOA	BKA	DBO	HHO	WOA	GWO	PSO
ALPT-1	Mean	−0.7908	−0.7086	**−0.8364**	−0.7729	−0.4450	−0.5493	−0.1942	−0.1348	−0.5475	−0.4987
	Std	0.0360	0.0608	0.0009	0.0525	0.0814	0.1292	0.2292	0.1381	0.0744	0.0828
	Best	−0.8372	−0.8160	−0.8372	−0.8372	−0.6670	−0.7705	−0.5727	−0.3711	−0.6800	−0.6349
ALPT-2	Mean	−1.6536	−1.5561	**−1.7520**	−1.6883	−0.9699	−1.2153	−0.9516	−0.7153	−1.2073	−1.1200
	Std	0.0443	0.0691	0.0036	0.0385	0.1660	0.1138	0.0999	0.1179	0.0831	0.1226
	Best	−1.7309	−1.6573	−1.7567	−1.7521	−1.4132	−1.4007	−1.1175	−1.0310	−1.3503	−1.2861
ALPT-3	Mean	−4.6803	−4.4447	**−4.8516**	−4.7565	−2.9457	−3.7846	−2.7832	−2.3985	−3.7035	−3.4308
	Std	0.0659	0.1280	0.0258	0.0748	0.3297	0.2416	0.1517	0.1869	0.1270	0.2443
	Best	−4.8134	−4.6801	−4.9069	−4.9067	−3.6423	−4.3218	−3.0402	−2.8541	−3.9105	−3.7639
ALPT-4	Mean	−5.6030	−5.2693	**−5.9284**	−5.6664	−3.3754	−4.4541	−3.4258	−3.0382	−4.0546	−3.9127
	Std	0.1187	0.1098	0.0320	0.1095	0.3468	0.2561	0.1959	0.2607	0.1847	0.2948
	Best	−5.7797	−5.5297	−5.9893	−5.9073	−4.1191	−4.9009	−3.8287	−3.6528	−4.4471	−4.5608
**Mean rank**		2.75	4.00	**1.00**	2.25	8.25	5.00	8.75	10.00	6.00	7.00

The objective values are the negative normalised net employment gain plus the equity and budget terms, so smaller is better; 30 independent runs per algorithm and instance. The best mean on each instance is in bold. Friedman *χ*^2^ = 35.67, *p* < 0.001. Std, standard deviation. Bold numeric entries mark the lowest mean on each instance and the lowest mean rank; the bold Mean rank row label is structural.

**Table 16:** Policy read-out of the best CRBMO solution on each instance.

Instance	Places	Programmes Opened	Net Employed	Spend/Budget	Cost per Job ($k)
ALPT-1	1980	31	104	0.952	98.5
ALPT-2	3159	41	215	0.935	78.0
ALPT-3	7822	78	531	1.000	71.9
ALPT-4	10,657	117	626	0.971	84.8

Net employed is the congested gross gain less the lock-in loss, in additional employed persons at the medium-run horizon. The cost per job is total spend divided by net employed.

**Table 17:** The four occupational retraining instances.

Instance	Sources *I*	Targets *J*	Dimension *D*	Displaced Workers	Budget ($M)
ORP-1	8	10	80	9843	13.2
ORP-2	12	16	192	13,989	18.7
ORP-3	18	24	432	24,316	32.5
ORP-4	25	34	850	32,839	43.9

Task vectors, displaced stocks, vacancies and wages are drawn from fixed seeds and are identical for every algorithm. The budget is set at 30% of the displaced stock times the base retraining cost, so that only a minority of displaced workers can be served and the planner is forced to choose.

**Table 18:** The objective values and mean ranks of CRBMO and the comparison algorithms on the four occupational retraining instances.

Instance	Metric	CRBMO	RBMO	CPO	SBOA	BKA	DBO	HHO	WOA	GWO	PSO
ORP-1	Mean	−39.7757	−36.8646	**−41.0779**	−39.9417	−24.9645	−30.2216	−22.2889	−19.3010	−37.0796	−32.1592
	Std	1.0998	2.4362	0.4755	0.9586	4.6459	2.8048	2.5514	2.9341	1.6715	2.5139
	Best	−41.5096	−40.3526	−41.9202	−41.7993	−34.1214	−34.1903	−28.1338	−25.5005	−39.9286	−35.7462
ORP-2	Mean	−53.2022	−50.7727	**−54.9771**	−53.6486	−37.0100	−42.9609	−30.6672	−26.3940	−49.1548	−50.3302
	Std	1.4771	2.1700	0.6919	1.0923	5.1213	2.8420	2.3814	2.9007	1.9203	1.9899
	Best	−55.6316	−53.4793	−56.1281	−56.2493	−45.0668	−48.8775	−35.4959	−31.3571	−52.0424	−54.2774
ORP-3	Mean	**−81.1158**	−78.5497	−78.3624	−80.1049	−58.4996	−70.8283	−55.9593	−51.9879	−75.3638	−73.6018
	Std	1.2830	2.0498	1.0301	1.7882	3.0029	2.2385	1.7577	2.7806	1.5697	2.8316
	Best	−83.7131	−82.1872	−80.2083	−83.2927	−64.3076	−75.7745	−60.9521	−58.8187	−79.3382	−79.3049
ORP-4	Mean	−79.2959	−75.2932	**−80.6287**	−78.8963	−53.1549	−68.2720	−54.7581	−51.3326	−54.3319	−57.5783
	Std	2.1638	2.2302	1.2103	2.6599	4.2099	2.8472	2.7893	2.4081	3.2479	4.2720
	Best	−84.8208	−79.4183	−83.6068	−83.3228	−62.9985	−73.5405	−59.8598	−55.9668	−61.2315	−65.6391
**Mean rank**		2.25	4.00	**1.75**	2.25	8.25	6.50	8.50	10.00	5.75	5.75

The objective values are the negative normalised discounted value plus the equity and budget terms, so smaller is better; 30 independent runs per algorithm and instance. The best mean on each instance is in bold. Friedman *χ*^2^ = 32.89, *p* < 0.001. Std, standard deviation. Bold numeric entries mark the lowest mean on each instance and the lowest mean rank; the bold Mean rank row label is structural.

**Table 19:** Policy read-out of the best CRBMO solution on each instance.

Instance	Workers	Pathways	Value ($M)	Spend/Budget	Upskilling Share
ORP-1	1263	25	45.5	1.000	0.195
ORP-2	1654	40	60.4	1.000	0.699
ORP-3	2653	74	89.7	1.001	0.714
ORP-4	3216	109	90.3	1.000	0.689

Value is the discounted earnings difference over the ten-year horizon against the no-retraining counterfactual, summed over completed transitions. Upskilling share is the fraction of expected completions that move a worker to a higher-skill occupation.

## Data Availability

All data generated and analysed in this study are contained in the accompanying data package. The two policy models are calibrated entirely from published estimates, cited at the point of use; no individual-level data were used.

## Supplementary Materials

The following supporting information can be downloaded at https://www.mdpi.com/article/10.3390/biomimetics11100723/s1. The data and code package accompanying this article contains the implementations of CRBMO and of the comparison algorithms, the binding to the official CEC2017 code with its validation check, the code of the two policy models, the raw results of all 30-run experiments together with the supplementary parameter sweeps and validation runs, the scripts that regenerate every table and figure in this paper, and a README.

## Appendix A

The four tables below report the full error statistics behind Section 5.4: for every algorithm, every CEC2017 function and every dimension, the mean and standard deviation of the error over the 30 independent runs.

**Table A1:** The error statistics of CRBMO and the comparison algorithms on the CEC2017 suite (*D* = 10).

Function	Metric	CRBMO	RBMO	CPO	SBOA	BKA	DBO	HHO	WOA	GWO	PSO
F1	Mean	1.38 × 10^3^	**8.27 × 10^−1^**	1.05 × 10^3^	1.73 × 10^3^	1.24 × 10^5^	1.79 × 10^6^	6.95 × 10^9^	2.25 × 10^9^	6.83 × 10^7^	2.93 × 10^3^
	Std	2.50 × 10^3^	1.52 × 10^0^	1.29 × 10^3^	2.05 × 10^3^	2.25 × 10^5^	9.50 × 10^6^	3.48 × 10^9^	1.84 × 10^9^	1.90 × 10^8^	2.44 × 10^3^
F3	Mean	**3.60 × 10^−14^**	6.90 × 10^−6^	5.47 × 10^−4^	7.34 × 10^−3^	5.15 × 10^1^	1.11 × 10^1^	1.05 × 10^4^	1.18 × 10^4^	1.81 × 10^3^	2.58 × 10^2^
	Std	3.50 × 10^−14^	2.35 × 10^−5^	5.90 × 10^−4^	1.40 × 10^−2^	8.36 × 10^1^	3.25 × 10^1^	3.25 × 10^3^	4.73 × 10^3^	1.90 × 10^3^	7.92 × 10^2^
F4	Mean	**4.39 × 10^−1^**	3.89 × 10^0^	2.24 × 10^0^	3.84 × 10^0^	8.26 × 10^0^	2.93 × 10^1^	4.32 × 10^2^	1.94 × 10^2^	1.27 × 10^1^	2.13 × 10^1^
	Std	3.47 × 10^−1^	1.41 × 10^1^	9.19 × 10^−1^	1.87 × 10^0^	1.64 × 10^1^	4.47 × 10^1^	3.08 × 10^2^	1.35 × 10^2^	1.22 × 10^1^	3.00 × 10^1^
F5	Mean	8.24 × 10^0^	1.10 × 10^1^	**6.21 × 10^0^**	1.14 × 10^1^	3.68 × 10^1^	3.91 × 10^1^	7.06 × 10^1^	7.44 × 10^1^	1.71 × 10^1^	1.40 × 10^1^
	Std	3.40 × 10^0^	4.27 × 10^0^	2.62 × 10^0^	5.97 × 10^0^	1.55 × 10^1^	1.28 × 10^1^	1.86 × 10^1^	2.03 × 10^1^	6.89 × 10^0^	5.02 × 10^0^
F6	Mean	2.24 × 10^−3^	6.30 × 10^−4^	**1.41 × 10^−4^**	2.08 × 10^−3^	2.52 × 10^1^	8.86 × 10^0^	4.24 × 10^1^	4.17 × 10^1^	1.05 × 10^0^	2.21 × 10^−1^
	Std	3.06 × 10^−3^	1.56 × 10^−3^	1.19 × 10^−4^	5.03 × 10^−3^	1.21 × 10^1^	8.88 × 10^0^	1.15 × 10^1^	1.35 × 10^1^	1.43 × 10^0^	8.44 × 10^−1^
F7	Mean	**1.69 × 10^1^**	1.93 × 10^1^	2.19 × 10^1^	2.41 × 10^1^	5.78 × 10^1^	4.50 × 10^1^	1.00 × 10^2^	1.09 × 10^2^	3.66 × 10^1^	2.16 × 10^1^
	Std	3.07 × 10^0^	3.67 × 10^0^	3.71 × 10^0^	6.01 × 10^0^	1.53 × 10^1^	1.46 × 10^1^	2.42 × 10^1^	2.57 × 10^1^	1.09 × 10^1^	5.37 × 10^0^
F8	Mean	7.16 × 10^0^	1.18 × 10^1^	**5.61 × 10^0^**	1.21 × 10^1^	2.30 × 10^1^	3.24 × 10^1^	4.30 × 10^1^	4.99 × 10^1^	1.73 × 10^1^	9.99 × 10^0^
	Std	2.51 × 10^0^	4.39 × 10^0^	2.72 × 10^0^	5.06 × 10^0^	8.53 × 10^0^	1.13 × 10^1^	1.12 × 10^1^	1.29 × 10^1^	8.71 × 10^0^	4.54 × 10^0^
F9	Mean	2.98 × 10^−3^	5.36 × 10^−2^	**1.34 × 10^−6^**	8.81 × 10^−2^	2.82 × 10^2^	5.61 × 10^1^	6.85 × 10^2^	7.29 × 10^2^	3.08 × 10^1^	2.11 × 10^−2^
	Std	1.63 × 10^−2^	1.93 × 10^−1^	5.12 × 10^−6^	1.79 × 10^−1^	1.17 × 10^2^	6.78 × 10^1^	2.52 × 10^2^	3.48 × 10^2^	4.84 × 10^1^	1.00 × 10^−1^
F10	Mean	5.09 × 10^2^	5.13 × 10^2^	**2.89 × 10^2^**	5.41 × 10^2^	8.43 × 10^2^	8.71 × 10^2^	1.43 × 10^3^	1.37 × 10^3^	6.68 × 10^2^	4.50 × 10^2^
	Std	2.41 × 10^2^	2.28 × 10^2^	1.88 × 10^2^	2.57 × 10^2^	2.43 × 10^2^	2.48 × 10^2^	2.90 × 10^2^	3.20 × 10^2^	4.25 × 10^2^	1.94 × 10^2^
F11	Mean	4.09 × 10^0^	4.62 × 10^0^	**1.84 × 10^0^**	8.58 × 10^0^	4.65 × 10^1^	1.05 × 10^2^	7.51 × 10^2^	7.59 × 10^2^	3.57 × 10^1^	1.49 × 10^1^
	Std	1.77 × 10^0^	2.22 × 10^0^	1.53 × 10^0^	5.07 × 10^0^	3.00 × 10^1^	9.30 × 10^1^	9.52 × 10^2^	9.33 × 10^2^	4.10 × 10^1^	4.66 × 10^1^
F12	Mean	3.17 × 10^2^	**1.79 × 10^2^**	4.43 × 10^3^	1.15 × 10^4^	1.87 × 10^4^	3.43 × 10^6^	8.86 × 10^7^	1.20 × 10^7^	5.31 × 10^5^	8.34 × 10^5^
	Std	1.36 × 10^2^	9.01 × 10^1^	6.07 × 10^3^	9.13 × 10^3^	1.81 × 10^4^	4.42 × 10^6^	4.07 × 10^8^	1.92 × 10^7^	7.99 × 10^5^	2.49 × 10^6^
F13	Mean	**1.06 × 10^1^**	1.10 × 10^1^	1.34 × 10^1^	3.32 × 10^3^	9.98 × 10^2^	1.65 × 10^4^	1.78 × 10^4^	1.88 × 10^4^	1.03 × 10^4^	7.01 × 10^3^
	Std	5.93 × 10^0^	5.22 × 10^0^	4.27 × 10^0^	4.18 × 10^3^	1.30 × 10^3^	1.61 × 10^4^	1.23 × 10^4^	1.49 × 10^4^	1.02 × 10^4^	7.40 × 10^3^
F14	Mean	**1.06 × 10^1^**	1.37 × 10^1^	1.08 × 10^1^	2.66 × 10^2^	8.84 × 10^1^	3.73 × 10^2^	9.58 × 10^2^	1.80 × 10^3^	1.51 × 10^3^	1.33 × 10^2^
	Std	7.95 × 10^0^	7.94 × 10^0^	6.16 × 10^0^	6.36 × 10^2^	3.77 × 10^1^	5.94 × 10^2^	1.39 × 10^3^	1.54 × 10^3^	1.91 × 10^3^	1.92 × 10^2^
F15	Mean	4.02 × 10^0^	3.96 × 10^0^	**2.13 × 10^0^**	5.35 × 10^2^	1.27 × 10^2^	1.10 × 10^3^	8.55 × 10^3^	8.15 × 10^3^	2.54 × 10^3^	4.03 × 10^2^
	Std	1.85 × 10^0^	2.09 × 10^0^	8.30 × 10^−1^	7.24 × 10^2^	8.70 × 10^1^	1.42 × 10^3^	3.28 × 10^3^	6.10 × 10^3^	2.98 × 10^3^	8.22 × 10^2^
F16	Mean	**6.74 × 10^0^**	3.09 × 10^1^	1.19 × 10^1^	1.67 × 10^1^	1.63 × 10^2^	1.81 × 10^2^	4.22 × 10^2^	3.69 × 10^2^	1.08 × 10^2^	1.69 × 10^2^
	Std	5.06 × 10^0^	4.31 × 10^1^	2.54 × 10^1^	3.88 × 10^1^	1.33 × 10^2^	1.42 × 10^2^	1.74 × 10^2^	1.92 × 10^2^	6.56 × 10^1^	1.44 × 10^2^
F17	Mean	3.32 × 10^1^	3.69 × 10^1^	**8.79 × 10^0^**	3.04 × 10^1^	6.02 × 10^1^	7.29 × 10^1^	1.41 × 10^2^	1.42 × 10^2^	6.33 × 10^1^	5.00 × 10^1^
	Std	1.12 × 10^1^	1.56 × 10^1^	5.52 × 10^0^	1.24 × 10^1^	1.49 × 10^1^	3.91 × 10^1^	8.24 × 10^1^	7.84 × 10^1^	3.48 × 10^1^	3.38 × 10^1^
F18	Mean	2.05 × 10^1^	1.35 × 10^1^	**9.88 × 10^0^**	5.05 × 10^3^	7.66 × 10^2^	2.14 × 10^4^	1.36 × 10^4^	2.01 × 10^4^	2.41 × 10^4^	2.02 × 10^4^
	Std	7.80 × 10^0^	8.26 × 10^0^	5.32 × 10^0^	3.99 × 10^3^	1.83 × 10^3^	1.64 × 10^4^	1.13 × 10^4^	1.35 × 10^4^	1.51 × 10^4^	1.75 × 10^4^
F19	Mean	3.50 × 10^0^	3.00 × 10^0^	**1.29 × 10^0^**	1.14 × 10^3^	4.82 × 10^1^	5.82 × 10^3^	8.90 × 10^4^	7.84 × 10^4^	6.23 × 10^3^	4.48 × 10^3^
	Std	1.21 × 10^0^	9.32 × 10^−1^	4.06 × 10^−1^	1.67 × 10^3^	3.53 × 10^1^	1.44 × 10^4^	2.10 × 10^5^	1.11 × 10^5^	6.26 × 10^3^	1.06 × 10^4^
F20	Mean	2.69 × 10^1^	4.74 × 10^1^	**2.45 × 10^−1^**	1.38 × 10^1^	9.48 × 10^1^	1.00 × 10^2^	2.00 × 10^2^	2.19 × 10^2^	9.97 × 10^1^	5.51 × 10^1^
	Std	2.12 × 10^1^	4.65 × 10^1^	3.59 × 10^−1^	1.16 × 10^1^	4.62 × 10^1^	7.02 × 10^1^	8.37 × 10^1^	8.98 × 10^1^	6.16 × 10^1^	5.52 × 10^1^
F21	Mean	1.71 × 10^2^	1.91 × 10^2^	1.78 × 10^2^	1.42 × 10^2^	1.81 × 10^2^	**1.19 × 10^2^**	2.53 × 10^2^	2.50 × 10^2^	2.10 × 10^2^	1.91 × 10^2^
	Std	5.22 × 10^1^	4.65 × 10^1^	5.06 × 10^1^	5.35 × 10^1^	6.60 × 10^1^	3.75 × 10^1^	4.38 × 10^1^	5.02 × 10^1^	3.01 × 10^1^	4.96 × 10^1^
F22	Mean	8.80 × 10^1^	9.86 × 10^1^	**8.74 × 10^1^**	9.20 × 10^1^	1.05 × 10^2^	1.08 × 10^2^	5.90 × 10^2^	3.84 × 10^2^	1.32 × 10^2^	1.49 × 10^2^
	Std	3.35 × 10^1^	1.38 × 10^1^	3.38 × 10^1^	2.71 × 10^1^	1.60 × 10^1^	1.34 × 10^1^	4.83 × 10^2^	4.06 × 10^2^	1.15 × 10^2^	1.77 × 10^2^
F23	Mean	**3.09 × 10^2^**	3.17 × 10^2^	3.09 × 10^2^	3.11 × 10^2^	3.46 × 10^2^	3.42 × 10^2^	4.01 × 10^2^	3.84 × 10^2^	3.26 × 10^2^	3.28 × 10^2^
	Std	3.23 × 10^0^	5.29 × 10^0^	3.81 × 10^0^	4.93 × 10^0^	2.85 × 10^1^	1.61 × 10^1^	4.06 × 10^1^	2.72 × 10^1^	1.13 × 10^1^	1.28 × 10^1^
F24	Mean	3.32 × 10^2^	3.27 × 10^2^	3.22 × 10^2^	3.24 × 10^2^	3.60 × 10^2^	**3.11 × 10^2^**	4.40 × 10^2^	4.09 × 10^2^	3.45 × 10^2^	3.50 × 10^2^
	Std	4.41 × 10^1^	6.19 × 10^1^	6.04 × 10^1^	6.13 × 10^1^	5.59 × 10^1^	1.02 × 10^2^	4.78 × 10^1^	6.37 × 10^1^	4.77 × 10^1^	7.14 × 10^1^
F25	Mean	4.18 × 10^2^	4.22 × 10^2^	4.19 × 10^2^	**4.15 × 10^2^**	4.20 × 10^2^	4.31 × 10^2^	7.67 × 10^2^	6.63 × 10^2^	4.36 × 10^2^	4.45 × 10^2^
	Std	2.34 × 10^1^	2.37 × 10^1^	2.33 × 10^1^	2.24 × 10^1^	6.23 × 10^1^	6.65 × 10^1^	2.34 × 10^2^	1.55 × 10^2^	1.56 × 10^1^	3.64 × 10^1^
F26	Mean	3.31 × 10^2^	3.84 × 10^2^	3.03 × 10^2^	**2.91 × 10^2^**	4.85 × 10^2^	4.83 × 10^2^	1.52 × 10^3^	1.19 × 10^3^	6.99 × 10^2^	4.58 × 10^2^
	Std	1.63 × 10^2^	2.73 × 10^2^	1.20 × 10^1^	8.56 × 10^1^	3.55 × 10^2^	2.26 × 10^2^	4.26 × 10^2^	4.25 × 10^2^	4.54 × 10^2^	2.47 × 10^2^
F27	Mean	3.92 × 10^2^	3.98 × 10^2^	**3.90 × 10^2^**	3.92 × 10^2^	4.05 × 10^2^	4.03 × 10^2^	5.05 × 10^2^	4.94 × 10^2^	4.06 × 10^2^	4.16 × 10^2^
	Std	2.61 × 10^0^	1.64 × 10^1^	1.10 × 10^0^	3.06 × 10^0^	1.83 × 10^1^	1.61 × 10^1^	4.85 × 10^1^	6.46 × 10^1^	2.78 × 10^1^	2.40 × 10^1^
F28	Mean	4.61 × 10^2^	5.04 × 10^2^	4.33 × 10^2^	**3.53 × 10^2^**	5.24 × 10^2^	5.22 × 10^2^	8.36 × 10^2^	8.18 × 10^2^	5.72 × 10^2^	5.83 × 10^2^
	Std	1.43 × 10^2^	1.76 × 10^2^	1.48 × 10^2^	1.24 × 10^2^	1.76 × 10^2^	1.04 × 10^2^	1.95 × 10^2^	1.59 × 10^2^	9.28 × 10^1^	1.28 × 10^2^
F29	Mean	**2.48 × 10^2^**	2.64 × 10^2^	2.54 × 10^2^	2.66 × 10^2^	3.16 × 10^2^	3.43 × 10^2^	5.72 × 10^2^	5.32 × 10^2^	3.02 × 10^2^	2.98 × 10^2^
	Std	1.31 × 10^1^	3.75 × 10^1^	1.02 × 10^1^	1.34 × 10^1^	4.39 × 10^1^	6.89 × 10^1^	1.07 × 10^2^	1.38 × 10^2^	4.78 × 10^1^	5.62 × 10^1^
F30	Mean	1.42 × 10^5^	2.70 × 10^5^	**1.73 × 10^3^**	4.45 × 10^4^	2.83 × 10^5^	1.00 × 10^6^	6.40 × 10^6^	4.62 × 10^6^	7.07 × 10^5^	6.21 × 10^5^
	Std	3.08 × 10^5^	7.70 × 10^5^	1.63 × 10^3^	1.62 × 10^5^	7.13 × 10^5^	1.53 × 10^6^	7.54 × 10^6^	5.99 × 10^6^	1.31 × 10^6^	9.89 × 10^5^

Values are given to three significant figures in scientific notation. Mean, mean error over 30 runs; Std, sample standard deviation. Bold marks the lowest mean error on each function, determined from the unrounded values.

**Table A2:** The error statistics of CRBMO and the comparison algorithms on the CEC2017 suite (*D* = 30).

Function	Metric	CRBMO	RBMO	CPO	SBOA	BKA	DBO	HHO	WOA	GWO	PSO
F1	Mean	**2.56 × 10^3^**	1.40 × 10^4^	4.21 × 10^3^	2.00 × 10^4^	7.23 × 10^9^	5.58 × 10^7^	4.53 × 10^10^	3.73 × 10^10^	1.99 × 10^9^	2.65 × 10^9^
	Std	2.74 × 10^3^	1.45 × 10^4^	3.16 × 10^3^	1.84 × 10^4^	4.37 × 10^9^	4.97 × 10^7^	8.95 × 10^9^	8.61 × 10^9^	1.40 × 10^9^	4.57 × 10^9^
F3	Mean	2.13 × 10^3^	**1.52 × 10^3^**	4.21 × 10^4^	2.84 × 10^4^	2.96 × 10^4^	7.28 × 10^4^	9.03 × 10^4^	2.37 × 10^5^	4.45 × 10^4^	3.55 × 10^4^
	Std	1.89 × 10^3^	9.62 × 10^2^	8.72 × 10^3^	7.42 × 10^3^	7.32 × 10^3^	1.50 × 10^4^	3.27 × 10^3^	7.66 × 10^4^	1.09 × 10^4^	1.72 × 10^4^
F4	Mean	**9.81 × 10^1^**	1.12 × 10^2^	1.03 × 10^2^	1.06 × 10^2^	5.94 × 10^2^	1.78 × 10^2^	1.13 × 10^4^	7.70 × 10^3^	2.26 × 10^2^	6.81 × 10^2^
	Std	1.67 × 10^1^	2.31 × 10^1^	2.06 × 10^1^	2.20 × 10^1^	4.33 × 10^2^	4.89 × 10^1^	3.59 × 10^3^	2.82 × 10^3^	1.07 × 10^2^	1.26 × 10^3^
F5	Mean	**5.51 × 10^1^**	7.43 × 10^1^	1.40 × 10^2^	1.07 × 10^2^	2.32 × 10^2^	2.56 × 10^2^	3.94 × 10^2^	4.11 × 10^2^	1.36 × 10^2^	9.77 × 10^1^
	Std	1.51 × 10^1^	2.52 × 10^1^	1.86 × 10^1^	2.98 × 10^1^	4.35 × 10^1^	4.86 × 10^1^	3.08 × 10^1^	3.84 × 10^1^	5.54 × 10^1^	2.27 × 10^1^
F6	Mean	4.71 × 10^−1^	1.16 × 10^0^	**4.74 × 10^−2^**	5.98 × 10^0^	5.91 × 10^1^	4.87 × 10^1^	8.33 × 10^1^	8.76 × 10^1^	1.03 × 10^1^	5.63 × 10^0^
	Std	2.08 × 10^−1^	8.34 × 10^−1^	3.08 × 10^−2^	6.07 × 10^0^	4.79 × 10^0^	1.02 × 10^1^	8.81 × 10^0^	1.01 × 10^1^	4.31 × 10^0^	3.07 × 10^0^
F7	Mean	**9.00 × 10^1^**	1.13 × 10^2^	2.01 × 10^2^	1.84 × 10^2^	5.44 × 10^2^	3.02 × 10^2^	6.64 × 10^2^	7.13 × 10^2^	2.08 × 10^2^	1.38 × 10^2^
	Std	1.80 × 10^1^	2.67 × 10^1^	1.57 × 10^1^	5.28 × 10^1^	5.79 × 10^1^	9.31 × 10^1^	4.15 × 10^1^	6.79 × 10^1^	6.03 × 10^1^	5.41 × 10^1^
F8	Mean	**5.14 × 10^1^**	7.16 × 10^1^	1.34 × 10^2^	9.35 × 10^1^	1.75 × 10^2^	2.03 × 10^2^	2.94 × 10^2^	3.17 × 10^2^	1.12 × 10^2^	8.34 × 10^1^
	Std	1.29 × 10^1^	1.74 × 10^1^	2.03 × 10^1^	1.58 × 10^1^	2.26 × 10^1^	5.29 × 10^1^	3.23 × 10^1^	2.94 × 10^1^	5.47 × 10^1^	2.23 × 10^1^
F9	Mean	7.53 × 10^1^	6.76 × 10^1^	**3.58 × 10^1^**	7.85 × 10^2^	4.26 × 10^3^	4.89 × 10^3^	6.87 × 10^3^	1.00 × 10^4^	1.79 × 10^3^	6.39 × 10^2^
	Std	1.51 × 10^2^	6.04 × 10^1^	4.29 × 10^1^	4.86 × 10^2^	7.71 × 10^2^	2.14 × 10^3^	1.12 × 10^3^	1.63 × 10^3^	1.16 × 10^3^	1.17 × 10^3^
F10	Mean	3.59 × 10^3^	4.00 × 10^3^	5.82 × 10^3^	3.75 × 10^3^	4.23 × 10^3^	5.06 × 10^3^	6.86 × 10^3^	7.42 × 10^3^	4.77 × 10^3^	**3.52 × 10^3^**
	Std	7.47 × 10^2^	7.02 × 10^2^	4.13 × 10^2^	6.44 × 10^2^	4.59 × 10^2^	9.02 × 10^2^	7.51 × 10^2^	6.16 × 10^2^	1.89 × 10^3^	8.81 × 10^2^
F11	Mean	**7.15 × 10^1^**	1.14 × 10^2^	1.17 × 10^2^	1.39 × 10^2^	3.65 × 10^2^	4.37 × 10^2^	9.98 × 10^3^	1.06 × 10^4^	8.36 × 10^2^	1.89 × 10^2^
	Std	4.39 × 10^1^	3.56 × 10^1^	2.90 × 10^1^	4.76 × 10^1^	1.12 × 10^2^	2.20 × 10^2^	2.68 × 10^3^	2.79 × 10^3^	7.23 × 10^2^	5.79 × 10^1^
F12	Mean	**1.36 × 10^5^**	2.55 × 10^5^	6.69 × 10^5^	1.18 × 10^6^	7.36 × 10^7^	5.71 × 10^7^	9.79 × 10^9^	4.07 × 10^9^	6.05 × 10^7^	1.11 × 10^8^
	Std	1.58 × 10^5^	4.26 × 10^5^	5.61 × 10^5^	7.72 × 10^5^	1.34 × 10^8^	9.69 × 10^7^	2.91 × 10^9^	1.57 × 10^9^	8.87 × 10^7^	4.39 × 10^8^
F13	Mean	**1.12 × 10^4^**	1.28 × 10^4^	1.35 × 10^4^	1.14 × 10^4^	3.34 × 10^5^	4.76 × 10^6^	3.24 × 10^9^	7.21 × 10^8^	2.10 × 10^7^	1.14 × 10^8^
	Std	1.35 × 10^4^	1.68 × 10^4^	1.31 × 10^4^	1.37 × 10^4^	6.51 × 10^5^	1.17 × 10^7^	4.53 × 10^9^	4.59 × 10^8^	6.95 × 10^7^	3.21 × 10^8^
F14	Mean	1.16 × 10^2^	**1.10 × 10^2^**	2.48 × 10^2^	2.31 × 10^4^	1.22 × 10^4^	1.64 × 10^5^	3.92 × 10^6^	3.92 × 10^6^	3.03 × 10^5^	6.24 × 10^4^
	Std	2.42 × 10^1^	2.78 × 10^1^	2.57 × 10^2^	1.97 × 10^4^	2.18 × 10^4^	1.91 × 10^5^	4.20 × 10^6^	4.02 × 10^6^	5.08 × 10^5^	8.44 × 10^4^
F15	Mean	**8.98 × 10^2^**	2.09 × 10^3^	3.56 × 10^3^	2.22 × 10^3^	3.44 × 10^4^	7.34 × 10^5^	2.18 × 10^8^	1.75 × 10^8^	8.91 × 10^5^	3.06 × 10^4^
	Std	4.71 × 10^2^	7.44 × 10^3^	4.85 × 10^3^	3.10 × 10^3^	2.45 × 10^4^	3.41 × 10^6^	1.97 × 10^8^	1.98 × 10^8^	1.71 × 10^6^	5.11 × 10^4^
F16	Mean	8.58 × 10^2^	9.11 × 10^2^	**8.52 × 10^2^**	9.02 × 10^2^	1.44 × 10^3^	1.73 × 10^3^	3.88 × 10^3^	3.20 × 10^3^	1.02 × 10^3^	9.04 × 10^2^
	Std	2.63 × 10^2^	2.76 × 10^2^	2.96 × 10^2^	2.76 × 10^2^	2.81 × 10^2^	3.82 × 10^2^	1.29 × 10^3^	6.33 × 10^2^	4.10 × 10^2^	3.15 × 10^2^
F17	Mean	2.15 × 10^2^	3.46 × 10^2^	**1.45 × 10^2^**	2.50 × 10^2^	6.09 × 10^2^	9.06 × 10^2^	2.13 × 10^3^	1.60 × 10^3^	3.67 × 10^2^	4.26 × 10^2^
	Std	1.00 × 10^2^	1.50 × 10^2^	8.62 × 10^1^	1.52 × 10^2^	2.03 × 10^2^	2.76 × 10^2^	3.43 × 10^3^	1.09 × 10^3^	2.01 × 10^2^	1.96 × 10^2^
F18	Mean	2.90 × 10^3^	**4.92 × 10^2^**	6.82 × 10^4^	2.24 × 10^5^	1.94 × 10^5^	1.67 × 10^6^	4.64 × 10^7^	3.56 × 10^7^	2.26 × 10^6^	1.02 × 10^6^
	Std	2.61 × 10^3^	1.45 × 10^2^	7.11 × 10^4^	1.93 × 10^5^	1.87 × 10^5^	2.50 × 10^6^	4.99 × 10^7^	3.62 × 10^7^	2.49 × 10^6^	7.39 × 10^5^
F19	Mean	**2.18 × 10^2^**	7.16 × 10^2^	4.01 × 10^3^	5.31 × 10^3^	1.66 × 10^5^	1.61 × 10^6^	4.83 × 10^8^	2.58 × 10^8^	3.40 × 10^6^	3.37 × 10^6^
	Std	1.24 × 10^2^	2.83 × 10^3^	7.23 × 10^3^	5.34 × 10^3^	2.11 × 10^5^	3.54 × 10^6^	4.53 × 10^8^	3.08 × 10^8^	9.47 × 10^6^	1.62 × 10^7^
F20	Mean	2.48 × 10^2^	3.61 × 10^2^	**2.18 × 10^2^**	3.35 × 10^2^	5.66 × 10^2^	7.13 × 10^2^	9.50 × 10^2^	1.02 × 10^3^	4.51 × 10^2^	3.31 × 10^2^
	Std	1.08 × 10^2^	1.29 × 10^2^	1.12 × 10^2^	1.39 × 10^2^	1.37 × 10^2^	2.18 × 10^2^	1.85 × 10^2^	2.20 × 10^2^	1.47 × 10^2^	1.85 × 10^2^
F21	Mean	**2.50 × 10^2^**	2.79 × 10^2^	3.29 × 10^2^	2.89 × 10^2^	4.48 × 10^2^	4.44 × 10^2^	5.92 × 10^2^	6.16 × 10^2^	3.14 × 10^2^	3.02 × 10^2^
	Std	1.52 × 10^1^	2.47 × 10^1^	2.06 × 10^1^	2.66 × 10^1^	5.84 × 10^1^	5.42 × 10^1^	6.10 × 10^1^	5.75 × 10^1^	4.63 × 10^1^	3.13 × 10^1^
F22	Mean	1.34 × 10^3^	1.88 × 10^3^	2.66 × 10^3^	**2.49 × 10^2^**	4.20 × 10^3^	2.37 × 10^3^	7.06 × 10^3^	7.18 × 10^3^	3.35 × 10^3^	1.61 × 10^3^
	Std	1.80 × 10^3^	2.13 × 10^3^	3.00 × 10^3^	8.07 × 10^2^	1.52 × 10^3^	2.07 × 10^3^	1.07 × 10^3^	1.20 × 10^3^	2.24 × 10^3^	1.50 × 10^3^
F23	Mean	**4.11 × 10^2^**	4.60 × 10^2^	4.76 × 10^2^	4.43 × 10^2^	7.92 × 10^2^	6.97 × 10^2^	1.22 × 10^3^	1.08 × 10^3^	4.82 × 10^2^	5.74 × 10^2^
	Std	1.72 × 10^1^	4.02 × 10^1^	1.91 × 10^1^	3.02 × 10^1^	8.34 × 10^1^	8.50 × 10^1^	2.10 × 10^2^	1.50 × 10^2^	4.65 × 10^1^	8.61 × 10^1^
F24	Mean	**4.81 × 10^2^**	5.38 × 10^2^	5.47 × 10^2^	5.30 × 10^2^	8.35 × 10^2^	7.61 × 10^2^	1.26 × 10^3^	1.10 × 10^3^	5.69 × 10^2^	6.74 × 10^2^
	Std	1.80 × 10^1^	5.06 × 10^1^	3.32 × 10^1^	3.15 × 10^1^	1.03 × 10^2^	9.45 × 10^1^	1.54 × 10^2^	2.05 × 10^2^	6.80 × 10^1^	8.29 × 10^1^
F25	Mean	3.99 × 10^2^	4.01 × 10^2^	**3.90 × 10^2^**	4.06 × 10^2^	6.08 × 10^2^	4.52 × 10^2^	2.14 × 10^3^	1.50 × 10^3^	5.15 × 10^2^	4.26 × 10^2^
	Std	1.83 × 10^1^	1.75 × 10^1^	8.48 × 10^0^	1.86 × 10^1^	8.92 × 10^1^	5.42 × 10^1^	4.74 × 10^2^	3.00 × 10^2^	6.08 × 10^1^	6.81 × 10^1^
F26	Mean	**1.49 × 10^3^**	2.07 × 10^3^	2.37 × 10^3^	1.91 × 10^3^	5.10 × 10^3^	4.19 × 10^3^	8.33 × 10^3^	7.61 × 10^3^	2.27 × 10^3^	2.53 × 10^3^
	Std	4.60 × 10^2^	5.70 × 10^2^	1.87 × 10^2^	1.15 × 10^3^	1.43 × 10^3^	1.04 × 10^3^	1.10 × 10^3^	1.37 × 10^3^	3.54 × 10^2^	7.17 × 10^2^
F27	Mean	5.22 × 10^2^	5.39 × 10^2^	**5.16 × 10^2^**	5.30 × 10^2^	7.09 × 10^2^	6.49 × 10^2^	1.60 × 10^3^	1.13 × 10^3^	5.64 × 10^2^	5.85 × 10^2^
	Std	1.28 × 10^1^	2.47 × 10^1^	9.75 × 10^0^	1.63 × 10^1^	1.09 × 10^2^	7.07 × 10^1^	4.26 × 10^2^	2.72 × 10^2^	2.45 × 10^1^	6.36 × 10^1^
F28	Mean	**4.17 × 10^2^**	4.55 × 10^2^	4.31 × 10^2^	4.45 × 10^2^	8.57 × 10^2^	7.84 × 10^2^	3.78 × 10^3^	3.17 × 10^3^	6.43 × 10^2^	6.25 × 10^2^
	Std	3.28 × 10^1^	3.03 × 10^1^	2.03 × 10^1^	2.83 × 10^1^	2.61 × 10^2^	7.43 × 10^2^	6.91 × 10^2^	6.29 × 10^2^	8.45 × 10^1^	2.39 × 10^2^
F29	Mean	8.11 × 10^2^	9.58 × 10^2^	**7.59 × 10^2^**	7.80 × 10^2^	1.63 × 10^3^	1.44 × 10^3^	4.50 × 10^3^	3.57 × 10^3^	9.95 × 10^2^	9.20 × 10^2^
	Std	1.76 × 10^2^	1.61 × 10^2^	1.22 × 10^2^	1.88 × 10^2^	3.73 × 10^2^	4.18 × 10^2^	1.37 × 10^3^	1.25 × 10^3^	1.92 × 10^2^	2.25 × 10^2^
F30	Mean	2.31 × 10^4^	1.61 × 10^4^	2.16 × 10^4^	**9.27 × 10^3^**	2.70 × 10^6^	2.95 × 10^6^	6.44 × 10^8^	2.79 × 10^8^	8.30 × 10^6^	8.65 × 10^5^
	Std	1.96 × 10^4^	1.07 × 10^4^	1.93 × 10^4^	5.40 × 10^3^	2.44 × 10^6^	4.10 × 10^6^	3.27 × 10^8^	1.94 × 10^8^	6.53 × 10^6^	2.25 × 10^6^

Values are given to three significant figures in scientific notation. Mean, mean error over 30 runs; Std, sample standard deviation. Bold marks the lowest mean error on each function, determined from the unrounded values.

**Table A3:** The error statistics of CRBMO and the comparison algorithms on the CEC2017 suite (*D* = 50).

Function	Metric	CRBMO	RBMO	CPO	SBOA	BKA	DBO	HHO	WOA	GWO	PSO
F1	Mean	**5.38 × 10^3^**	2.50 × 10^7^	2.06 × 10^5^	3.21 × 10^7^	3.52 × 10^10^	2.51 × 10^9^	1.02 × 10^11^	8.59 × 10^10^	1.02 × 10^10^	6.79 × 10^9^
	Std	6.25 × 10^3^	1.97 × 10^7^	8.95 × 10^4^	9.33 × 10^7^	8.57 × 10^9^	8.28 × 10^9^	7.20 × 10^9^	9.95 × 10^9^	4.92 × 10^9^	4.43 × 10^9^
F3	Mean	4.30 × 10^4^	**3.75 × 10^4^**	1.55 × 10^5^	9.67 × 10^4^	9.22 × 10^4^	2.14 × 10^5^	2.31 × 10^5^	2.96 × 10^5^	1.10 × 10^5^	1.85 × 10^5^
	Std	1.18 × 10^4^	1.34 × 10^4^	2.25 × 10^4^	1.20 × 10^4^	1.40 × 10^4^	3.96 × 10^4^	3.42 × 10^4^	9.41 × 10^4^	1.47 × 10^4^	4.97 × 10^4^
F4	Mean	**1.53 × 10^2^**	2.46 × 10^2^	1.90 × 10^2^	2.34 × 10^2^	4.76 × 10^3^	4.70 × 10^2^	3.20 × 10^4^	2.43 × 10^4^	8.68 × 10^2^	7.97 × 10^2^
	Std	5.76 × 10^1^	4.85 × 10^1^	4.27 × 10^1^	5.69 × 10^1^	1.60 × 10^3^	1.36 × 10^2^	5.76 × 10^3^	5.34 × 10^3^	4.45 × 10^2^	6.29 × 10^2^
F5	Mean	**1.29 × 10^2^**	1.82 × 10^2^	3.46 × 10^2^	2.55 × 10^2^	3.88 × 10^2^	4.85 × 10^2^	6.42 × 10^2^	6.75 × 10^2^	2.33 × 10^2^	1.97 × 10^2^
	Std	3.08 × 10^1^	3.49 × 10^1^	2.84 × 10^1^	3.28 × 10^1^	3.80 × 10^1^	1.18 × 10^2^	3.40 × 10^1^	3.71 × 10^1^	5.96 × 10^1^	4.92 × 10^1^
F6	Mean	4.23 × 10^0^	9.11 × 10^0^	**9.59 × 10^−1^**	2.05 × 10^1^	7.09 × 10^1^	6.53 × 10^1^	9.59 × 10^1^	1.01 × 10^2^	2.50 × 10^1^	1.62 × 10^1^
	Std	1.85 × 10^0^	3.54 × 10^0^	5.89 × 10^−1^	1.09 × 10^1^	4.77 × 10^0^	9.99 × 10^0^	6.33 × 10^0^	5.88 × 10^0^	5.79 × 10^0^	5.79 × 10^0^
F7	Mean	**1.98 × 10^2^**	2.67 × 10^2^	4.63 × 10^2^	5.14 × 10^2^	1.05 × 10^3^	6.57 × 10^2^	1.21 × 10^3^	1.33 × 10^3^	4.24 × 10^2^	3.84 × 10^2^
	Std	2.60 × 10^1^	4.57 × 10^1^	3.79 × 10^1^	1.15 × 10^2^	7.97 × 10^1^	1.31 × 10^2^	5.48 × 10^1^	7.61 × 10^1^	1.09 × 10^2^	1.14 × 10^2^
F8	Mean	**1.38 × 10^2^**	1.82 × 10^2^	3.50 × 10^2^	2.57 × 10^2^	4.15 × 10^2^	5.08 × 10^2^	6.37 × 10^2^	6.80 × 10^2^	2.57 × 10^2^	2.02 × 10^2^
	Std	2.89 × 10^1^	3.28 × 10^1^	2.51 × 10^1^	3.42 × 10^1^	3.81 × 10^1^	1.03 × 10^2^	4.31 × 10^1^	5.85 × 10^1^	7.32 × 10^1^	6.35 × 10^1^
F9	Mean	**8.01 × 10^2^**	1.67 × 10^3^	2.45 × 10^3^	5.67 × 10^3^	1.56 × 10^4^	2.29 × 10^4^	2.66 × 10^4^	3.69 × 10^4^	1.20 × 10^4^	8.52 × 10^3^
	Std	5.79 × 10^2^	1.45 × 10^3^	1.51 × 10^3^	2.08 × 10^3^	1.45 × 10^3^	7.58 × 10^3^	3.46 × 10^3^	5.83 × 10^3^	4.64 × 10^3^	6.60 × 10^3^
F10	Mean	7.02 × 10^3^	8.47 × 10^3^	1.16 × 10^4^	**6.58 × 10^3^**	8.19 × 10^3^	9.26 × 10^3^	1.32 × 10^4^	1.35 × 10^4^	8.16 × 10^3^	6.61 × 10^3^
	Std	8.59 × 10^2^	9.18 × 10^2^	7.37 × 10^2^	1.11 × 10^3^	5.79 × 10^2^	1.77 × 10^3^	7.58 × 10^2^	8.37 × 10^2^	3.03 × 10^3^	1.06 × 10^3^
F11	Mean	**2.11 × 10^2^**	3.55 × 10^2^	3.53 × 10^2^	3.15 × 10^2^	2.80 × 10^3^	1.34 × 10^3^	2.13 × 10^4^	1.93 × 10^4^	5.03 × 10^3^	6.65 × 10^2^
	Std	5.17 × 10^1^	1.05 × 10^2^	6.32 × 10^1^	7.32 × 10^1^	1.56 × 10^3^	1.13 × 10^3^	3.51 × 10^3^	4.22 × 10^3^	2.60 × 10^3^	1.18 × 10^3^
F12	Mean	**3.64 × 10^6^**	9.13 × 10^6^	6.01 × 10^6^	9.99 × 10^6^	3.95 × 10^9^	4.74 × 10^8^	6.49 × 10^10^	3.30 × 10^10^	1.74 × 10^9^	3.32 × 10^9^
	Std	2.78 × 10^6^	7.75 × 10^6^	2.88 × 10^6^	5.23 × 10^6^	3.34 × 10^9^	3.53 × 10^8^	1.73 × 10^10^	9.76 × 10^9^	1.74 × 10^9^	2.76 × 10^9^
F13	Mean	1.00 × 10^4^	1.86 × 10^4^	2.03 × 10^4^	**6.31 × 10^3^**	2.61 × 10^8^	3.46 × 10^7^	2.91 × 10^10^	1.32 × 10^10^	1.53 × 10^8^	8.60 × 10^8^
	Std	9.54 × 10^3^	1.31 × 10^4^	1.54 × 10^4^	7.67 × 10^3^	7.87 × 10^8^	3.77 × 10^7^	1.09 × 10^10^	8.14 × 10^9^	1.48 × 10^8^	1.28 × 10^9^
F14	Mean	5.55 × 10^2^	**4.06 × 10^2^**	4.30 × 10^4^	2.61 × 10^5^	1.56 × 10^5^	2.14 × 10^6^	5.98 × 10^7^	2.32 × 10^7^	9.77 × 10^5^	1.72 × 10^6^
	Std	2.29 × 10^2^	7.24 × 10^1^	4.26 × 10^4^	1.72 × 10^5^	1.53 × 10^5^	2.93 × 10^6^	8.54 × 10^7^	2.17 × 10^7^	1.04 × 10^6^	4.27 × 10^6^
F15	Mean	8.72 × 10^3^	8.56 × 10^3^	9.63 × 10^3^	**5.87 × 10^3^**	3.22 × 10^6^	5.81 × 10^7^	5.87 × 10^9^	2.56 × 10^9^	1.90 × 10^7^	1.40 × 10^7^
	Std	7.77 × 10^3^	7.36 × 10^3^	6.90 × 10^3^	5.08 × 10^3^	8.12 × 10^6^	1.74 × 10^8^	3.63 × 10^9^	3.03 × 10^9^	2.42 × 10^7^	7.64 × 10^7^
F16	Mean	1.65 × 10^3^	1.90 × 10^3^	2.10 × 10^3^	**1.51 × 10^3^**	2.77 × 10^3^	3.06 × 10^3^	6.48 × 10^3^	6.32 × 10^3^	1.80 × 10^3^	1.62 × 10^3^
	Std	3.61 × 10^2^	3.81 × 10^2^	5.20 × 10^2^	4.51 × 10^2^	5.75 × 10^2^	7.21 × 10^2^	1.47 × 10^3^	1.52 × 10^3^	4.50 × 10^2^	4.84 × 10^2^
F17	Mean	**1.11 × 10^3^**	1.38 × 10^3^	1.17 × 10^3^	1.19 × 10^3^	1.90 × 10^3^	2.28 × 10^3^	7.28 × 10^3^	4.37 × 10^3^	1.38 × 10^3^	1.59 × 10^3^
	Std	3.30 × 10^2^	2.79 × 10^2^	2.61 × 10^2^	2.69 × 10^2^	4.53 × 10^2^	5.86 × 10^2^	5.98 × 10^3^	1.63 × 10^3^	3.81 × 10^2^	3.45 × 10^2^
F18	Mean	6.32 × 10^4^	**5.82 × 10^4^**	9.03 × 10^5^	2.64 × 10^6^	1.33 × 10^6^	9.17 × 10^6^	1.28 × 10^8^	1.36 × 10^8^	8.39 × 10^6^	5.01 × 10^6^
	Std	2.08 × 10^4^	3.23 × 10^4^	8.47 × 10^5^	1.55 × 10^6^	1.53 × 10^6^	1.11 × 10^7^	9.07 × 10^7^	1.16 × 10^8^	6.70 × 10^6^	3.84 × 10^6^
F19	Mean	1.68 × 10^4^	2.05 × 10^4^	1.70 × 10^4^	**1.53 × 10^4^**	1.12 × 10^6^	5.87 × 10^6^	2.93 × 10^9^	9.38 × 10^8^	5.12 × 10^6^	4.55 × 10^7^
	Std	1.25 × 10^4^	1.35 × 10^4^	7.60 × 10^3^	9.46 × 10^3^	8.50 × 10^5^	7.23 × 10^6^	1.57 × 10^9^	5.90 × 10^8^	8.58 × 10^6^	2.41 × 10^8^
F20	Mean	9.93 × 10^2^	1.04 × 10^3^	1.15 × 10^3^	9.73 × 10^2^	1.25 × 10^3^	1.63 × 10^3^	2.02 × 10^3^	2.13 × 10^3^	1.11 × 10^3^	**9.62 × 10^2^**
	Std	2.94 × 10^2^	3.24 × 10^2^	2.52 × 10^2^	2.76 × 10^2^	2.68 × 10^2^	2.95 × 10^2^	3.28 × 10^2^	2.16 × 10^2^	5.13 × 10^2^	3.07 × 10^2^
F21	Mean	**3.12 × 10^2^**	4.09 × 10^2^	5.28 × 10^2^	4.09 × 10^2^	7.49 × 10^2^	7.60 × 10^2^	1.06 × 10^3^	1.07 × 10^3^	4.53 × 10^2^	4.30 × 10^2^
	Std	1.89 × 10^1^	4.06 × 10^1^	2.70 × 10^1^	3.51 × 10^1^	8.30 × 10^1^	7.36 × 10^1^	1.07 × 10^2^	8.71 × 10^1^	7.30 × 10^1^	6.54 × 10^1^
F22	Mean	7.59 × 10^3^	8.67 × 10^3^	1.17 × 10^4^	**6.69 × 10^3^**	8.79 × 10^3^	9.86 × 10^3^	1.40 × 10^4^	1.39 × 10^4^	7.77 × 10^3^	7.63 × 10^3^
	Std	1.15 × 10^3^	1.38 × 10^3^	2.27 × 10^3^	2.35 × 10^3^	6.43 × 10^2^	1.93 × 10^3^	6.01 × 10^2^	1.01 × 10^3^	2.41 × 10^3^	1.59 × 10^3^
F23	Mean	**5.69 × 10^2^**	7.37 × 10^2^	7.63 × 10^2^	6.91 × 10^2^	1.47 × 10^3^	1.21 × 10^3^	1.97 × 10^3^	1.92 × 10^3^	7.39 × 10^2^	1.03 × 10^3^
	Std	2.62 × 10^1^	8.23 × 10^1^	3.70 × 10^1^	5.91 × 10^1^	1.66 × 10^2^	1.59 × 10^2^	1.81 × 10^2^	1.89 × 10^2^	1.02 × 10^2^	1.17 × 10^2^
F24	Mean	**6.33 × 10^2^**	8.07 × 10^2^	8.42 × 10^2^	7.49 × 10^2^	1.49 × 10^3^	1.36 × 10^3^	2.21 × 10^3^	2.01 × 10^3^	8.14 × 10^2^	1.17 × 10^3^
	Std	3.23 × 10^1^	7.23 × 10^1^	2.82 × 10^1^	5.61 × 10^1^	1.37 × 10^2^	1.73 × 10^2^	2.14 × 10^2^	1.91 × 10^2^	1.28 × 10^2^	1.43 × 10^2^
F25	Mean	**5.69 × 10^2^**	6.40 × 10^2^	6.28 × 10^2^	6.74 × 10^2^	2.95 × 10^3^	7.54 × 10^2^	1.17 × 10^4^	9.61 × 10^3^	1.30 × 10^3^	8.44 × 10^2^
	Std	3.12 × 10^1^	4.62 × 10^1^	2.54 × 10^1^	3.42 × 10^1^	8.61 × 10^2^	1.24 × 10^2^	1.35 × 10^3^	1.65 × 10^3^	3.01 × 10^2^	3.17 × 10^2^
F26	Mean	**2.64 × 10^3^**	3.77 × 10^3^	4.58 × 10^3^	4.12 × 10^3^	9.70 × 10^3^	7.79 × 10^3^	1.43 × 10^4^	1.41 × 10^4^	4.45 × 10^3^	4.73 × 10^3^
	Std	2.91 × 10^2^	1.25 × 10^3^	3.83 × 10^2^	2.15 × 10^3^	1.78 × 10^3^	1.44 × 10^3^	6.93 × 10^2^	1.01 × 10^3^	7.75 × 10^2^	1.35 × 10^3^
F27	Mean	7.60 × 10^2^	7.44 × 10^2^	**6.63 × 10^2^**	8.49 × 10^2^	1.52 × 10^3^	1.26 × 10^3^	4.16 × 10^3^	3.84 × 10^3^	9.67 × 10^2^	1.04 × 10^3^
	Std	9.24 × 10^1^	1.16 × 10^2^	7.18 × 10^1^	1.32 × 10^2^	3.42 × 10^2^	2.60 × 10^2^	9.70 × 10^2^	1.14 × 10^3^	1.21 × 10^2^	2.77 × 10^2^
F28	Mean	**5.44 × 10^2^**	7.46 × 10^2^	6.12 × 10^2^	6.69 × 10^2^	3.28 × 10^3^	2.28 × 10^3^	9.78 × 10^3^	7.68 × 10^3^	1.84 × 10^3^	1.93 × 10^3^
	Std	2.86 × 10^1^	2.43 × 10^2^	4.79 × 10^1^	5.91 × 10^1^	7.15 × 10^2^	2.26 × 10^3^	1.20 × 10^3^	1.02 × 10^3^	4.63 × 10^2^	1.51 × 10^3^
F29	Mean	1.46 × 10^3^	1.64 × 10^3^	1.43 × 10^3^	**1.43 × 10^3^**	3.76 × 10^3^	3.51 × 10^3^	5.13 × 10^4^	2.05 × 10^4^	1.94 × 10^3^	1.56 × 10^3^
	Std	3.37 × 10^2^	3.35 × 10^2^	3.14 × 10^2^	3.50 × 10^2^	1.04 × 10^3^	8.34 × 10^2^	7.63 × 10^4^	1.23 × 10^4^	4.51 × 10^2^	4.08 × 10^2^
F30	Mean	4.02 × 10^6^	2.37 × 10^6^	1.75 × 10^6^	**1.19 × 10^6^**	6.28 × 10^7^	3.91 × 10^7^	5.27 × 10^9^	1.97 × 10^9^	1.42 × 10^8^	7.80 × 10^7^
	Std	2.03 × 10^6^	1.18 × 10^6^	5.80 × 10^5^	2.48 × 10^5^	3.06 × 10^7^	4.67 × 10^7^	2.56 × 10^9^	1.36 × 10^9^	8.95 × 10^7^	3.67 × 10^8^

Values are given to three significant figures in scientific notation. Mean, mean error over 30 runs; Std, sample standard deviation. Bold marks the lowest mean error on each function, determined from the unrounded values.

**Table A4:** The error statistics of CRBMO and the comparison algorithms on the CEC2017 suite (*D* = 100).

Function	Metric	CRBMO	RBMO	CPO	SBOA	BKA	DBO	HHO	WOA	GWO	PSO
F1	Mean	**1.34 × 10^5^**	5.55 × 10^9^	3.09 × 10^8^	3.59 × 10^9^	1.45 × 10^11^	4.23 × 10^10^	2.55 × 10^11^	2.37 × 10^11^	5.02 × 10^10^	4.76 × 10^10^
	Std	1.09 × 10^5^	1.49 × 10^9^	1.33 × 10^8^	2.61 × 10^9^	1.78 × 10^10^	5.23 × 10^10^	1.16 × 10^10^	1.71 × 10^10^	1.12 × 10^10^	1.97 × 10^10^
F3	Mean	2.39 × 10^5^	**2.23 × 10^5^**	4.55 × 10^5^	2.94 × 10^5^	2.70 × 10^5^	4.71 × 10^5^	3.65 × 10^5^	8.57 × 10^5^	4.08 × 10^5^	5.72 × 10^5^
	Std	3.42 × 10^4^	3.52 × 10^4^	5.21 × 10^4^	1.93 × 10^4^	1.59 × 10^4^	1.67 × 10^5^	1.04 × 10^4^	1.63 × 10^5^	5.72 × 10^4^	1.04 × 10^5^
F4	Mean	**4.57 × 10^2^**	1.29 × 10^3^	6.90 × 10^2^	9.84 × 10^2^	2.22 × 10^4^	7.28 × 10^3^	9.40 × 10^4^	7.26 × 10^4^	4.60 × 10^3^	5.49 × 10^3^
	Std	6.45 × 10^1^	4.75 × 10^2^	7.68 × 10^1^	2.00 × 10^2^	7.51 × 10^3^	9.42 × 10^3^	1.19 × 10^4^	1.12 × 10^4^	1.29 × 10^3^	3.53 × 10^3^
F5	Mean	**3.84 × 10^2^**	6.85 × 10^2^	1.02 × 10^3^	7.56 × 10^2^	1.02 × 10^3^	1.14 × 10^3^	1.53 × 10^3^	1.60 × 10^3^	7.26 × 10^2^	7.29 × 10^2^
	Std	5.62 × 10^1^	8.92 × 10^1^	5.49 × 10^1^	6.20 × 10^1^	7.94 × 10^1^	2.46 × 10^2^	5.93 × 10^1^	6.26 × 10^1^	1.29 × 10^2^	1.58 × 10^2^
F6	Mean	1.98 × 10^1^	3.10 × 10^1^	**1.61 × 10^1^**	5.00 × 10^1^	7.84 × 10^1^	7.39 × 10^1^	1.05 × 10^2^	1.11 × 10^2^	4.28 × 10^1^	4.29 × 10^1^
	Std	3.60 × 10^0^	5.96 × 10^0^	3.34 × 10^0^	9.85 × 10^0^	3.56 × 10^0^	1.21 × 10^1^	4.28 × 10^0^	6.50 × 10^0^	3.33 × 10^0^	7.18 × 10^0^
F7	Mean	**7.50 × 10^2^**	1.12 × 10^3^	1.45 × 10^3^	1.70 × 10^3^	2.66 × 10^3^	2.12 × 10^3^	3.08 × 10^3^	3.30 × 10^3^	1.41 × 10^3^	1.33 × 10^3^
	Std	1.21 × 10^2^	1.40 × 10^2^	1.18 × 10^2^	2.63 × 10^2^	1.21 × 10^2^	1.65 × 10^2^	7.04 × 10^1^	1.06 × 10^2^	1.46 × 10^2^	2.66 × 10^2^
F8	Mean	**3.74 × 10^2^**	6.63 × 10^2^	1.02 × 10^3^	8.02 × 10^2^	1.16 × 10^3^	1.26 × 10^3^	1.69 × 10^3^	1.78 × 10^3^	7.48 × 10^2^	7.36 × 10^2^
	Std	5.72 × 10^1^	7.65 × 10^1^	6.04 × 10^1^	8.00 × 10^1^	9.33 × 10^1^	2.39 × 10^2^	8.10 × 10^1^	6.34 × 10^1^	1.63 × 10^2^	1.30 × 10^2^
F9	Mean	**8.38 × 10^3^**	1.84 × 10^4^	3.48 × 10^4^	2.85 × 10^4^	3.45 × 10^4^	6.65 × 10^4^	5.96 × 10^4^	7.75 × 10^4^	4.39 × 10^4^	5.42 × 10^4^
	Std	1.97 × 10^3^	5.28 × 10^3^	6.72 × 10^3^	3.62 × 10^3^	3.12 × 10^3^	1.39 × 10^4^	4.57 × 10^3^	6.74 × 10^3^	8.56 × 10^3^	2.07 × 10^4^
F10	Mean	1.76 × 10^4^	2.23 × 10^4^	2.88 × 10^4^	**1.66 × 10^4^**	1.86 × 10^4^	2.51 × 10^4^	3.04 × 10^4^	3.09 × 10^4^	1.91 × 10^4^	2.04 × 10^4^
	Std	2.23 × 10^3^	2.06 × 10^3^	6.55 × 10^2^	1.75 × 10^3^	1.34 × 10^3^	5.33 × 10^3^	1.14 × 10^3^	1.42 × 10^3^	6.39 × 10^3^	3.97 × 10^3^
F11	Mean	**7.20 × 10^3^**	1.12 × 10^4^	4.31 × 10^4^	3.16 × 10^4^	5.99 × 10^4^	1.74 × 10^5^	3.13 × 10^5^	3.56 × 10^5^	6.13 × 10^4^	4.52 × 10^4^
	Std	2.40 × 10^3^	4.36 × 10^3^	8.29 × 10^3^	9.41 × 10^3^	1.54 × 10^4^	5.38 × 10^4^	9.20 × 10^4^	1.17 × 10^5^	1.41 × 10^4^	2.24 × 10^4^
F12	Mean	**6.61 × 10^7^**	4.05 × 10^8^	8.05 × 10^7^	3.16 × 10^8^	4.92 × 10^10^	3.36 × 10^9^	1.80 × 10^11^	1.50 × 10^11^	8.23 × 10^9^	1.45 × 10^10^
	Std	3.23 × 10^7^	3.25 × 10^8^	3.41 × 10^7^	1.07 × 10^8^	1.74 × 10^10^	1.22 × 10^9^	2.05 × 10^10^	2.36 × 10^10^	3.24 × 10^9^	9.39 × 10^9^
F13	Mean	**2.07 × 10^4^**	4.66 × 10^4^	3.50 × 10^4^	3.23 × 10^4^	7.47 × 10^9^	1.99 × 10^8^	4.15 × 10^10^	3.09 × 10^10^	1.30 × 10^9^	1.35 × 10^9^
	Std	8.24 × 10^3^	1.81 × 10^4^	1.01 × 10^4^	1.28 × 10^4^	4.49 × 10^9^	2.00 × 10^8^	7.08 × 10^9^	5.78 × 10^9^	1.31 × 10^9^	1.53 × 10^9^
F14	Mean	**3.31 × 10^5^**	4.78 × 10^5^	2.03 × 10^6^	2.07 × 10^6^	2.58 × 10^6^	1.14 × 10^7^	7.30 × 10^7^	4.00 × 10^7^	8.56 × 10^6^	6.61 × 10^6^
	Std	2.94 × 10^5^	4.57 × 10^5^	1.14 × 10^6^	9.30 × 10^5^	1.60 × 10^6^	9.90 × 10^6^	4.42 × 10^7^	2.57 × 10^7^	5.26 × 10^6^	6.27 × 10^6^
F15	Mean	4.52 × 10^3^	1.19 × 10^4^	1.48 × 10^4^	**4.45 × 10^3^**	9.23 × 10^8^	2.85 × 10^7^	1.89 × 10^10^	1.14 × 10^10^	2.57 × 10^8^	3.80 × 10^8^
	Std	4.70 × 10^3^	6.61 × 10^3^	4.69 × 10^3^	2.96 × 10^3^	1.06 × 10^9^	5.72 × 10^7^	5.03 × 10^9^	3.96 × 10^9^	2.76 × 10^8^	6.44 × 10^8^
F16	Mean	**4.18 × 10^3^**	5.41 × 10^3^	7.25 × 10^3^	4.34 × 10^3^	8.29 × 10^3^	7.29 × 10^3^	2.24 × 10^4^	2.10 × 10^4^	4.93 × 10^3^	4.88 × 10^3^
	Std	5.64 × 10^2^	7.23 × 10^2^	6.40 × 10^2^	7.33 × 10^2^	1.47 × 10^3^	1.19 × 10^3^	4.15 × 10^3^	3.28 × 10^3^	1.07 × 10^3^	8.14 × 10^2^
F17	Mean	**3.26 × 10^3^**	3.93 × 10^3^	4.45 × 10^3^	3.27 × 10^3^	9.08 × 10^3^	7.22 × 10^3^	6.23 × 10^6^	2.49 × 10^6^	4.13 × 10^3^	5.58 × 10^3^
	Std	3.54 × 10^2^	4.76 × 10^2^	5.13 × 10^2^	5.26 × 10^2^	6.21 × 10^3^	1.33 × 10^3^	7.94 × 10^6^	3.96 × 10^6^	2.03 × 10^3^	4.19 × 10^3^
F18	Mean	**6.35 × 10^5^**	6.79 × 10^5^	3.16 × 10^6^	2.98 × 10^6^	3.06 × 10^6^	2.03 × 10^7^	1.04 × 10^8^	6.11 × 10^7^	1.03 × 10^7^	1.15 × 10^7^
	Std	4.02 × 10^5^	5.13 × 10^5^	2.12 × 10^6^	1.33 × 10^6^	1.62 × 10^6^	9.51 × 10^6^	6.63 × 10^7^	6.48 × 10^7^	7.05 × 10^6^	5.39 × 10^6^
F19	Mean	**3.27 × 10^3^**	1.44 × 10^4^	1.74 × 10^4^	4.33 × 10^3^	8.90 × 10^8^	4.60 × 10^7^	2.05 × 10^10^	1.04 × 10^10^	1.38 × 10^8^	4.70 × 10^8^
	Std	4.96 × 10^3^	2.54 × 10^4^	1.43 × 10^4^	4.17 × 10^3^	1.43 × 10^9^	4.90 × 10^7^	5.96 × 10^9^	4.00 × 10^9^	1.24 × 10^8^	9.06 × 10^8^
F20	Mean	3.12 × 10^3^	3.27 × 10^3^	4.78 × 10^3^	**2.96 × 10^3^**	3.34 × 10^3^	4.54 × 10^3^	5.37 × 10^3^	5.68 × 10^3^	3.93 × 10^3^	3.58 × 10^3^
	Std	5.29 × 10^2^	4.29 × 10^2^	5.83 × 10^2^	4.37 × 10^2^	4.24 × 10^2^	7.58 × 10^2^	4.70 × 10^2^	5.12 × 10^2^	1.30 × 10^3^	8.21 × 10^2^
F21	Mean	**6.15 × 10^2^**	1.06 × 10^3^	1.18 × 10^3^	9.11 × 10^2^	2.02 × 10^3^	1.83 × 10^3^	2.73 × 10^3^	2.61 × 10^3^	9.55 × 10^2^	1.22 × 10^3^
	Std	6.91 × 10^1^	1.22 × 10^2^	5.52 × 10^1^	6.16 × 10^1^	2.21 × 10^2^	1.61 × 10^2^	1.85 × 10^2^	2.12 × 10^2^	1.84 × 10^2^	1.71 × 10^2^
F22	Mean	1.91 × 10^4^	2.31 × 10^4^	2.97 × 10^4^	**1.88 × 10^4^**	2.10 × 10^4^	2.51 × 10^4^	3.15 × 10^4^	3.21 × 10^4^	2.08 × 10^4^	2.11 × 10^4^
	Std	2.26 × 10^3^	1.72 × 10^3^	9.26 × 10^2^	1.34 × 10^3^	1.30 × 10^3^	5.31 × 10^3^	1.15 × 10^3^	8.71 × 10^2^	6.19 × 10^3^	3.90 × 10^3^
F23	Mean	**1.01 × 10^3^**	1.62 × 10^3^	1.36 × 10^3^	1.17 × 10^3^	2.87 × 10^3^	2.53 × 10^3^	4.10 × 10^3^	3.94 × 10^3^	1.35 × 10^3^	2.38 × 10^3^
	Std	9.20 × 10^1^	1.86 × 10^2^	6.22 × 10^1^	7.71 × 10^1^	2.80 × 10^2^	2.65 × 10^2^	6.09 × 10^2^	4.57 × 10^2^	9.04 × 10^1^	3.15 × 10^2^
F24	Mean	**1.44 × 10^3^**	2.28 × 10^3^	1.87 × 10^3^	1.87 × 10^3^	4.23 × 10^3^	3.60 × 10^3^	7.24 × 10^3^	6.96 × 10^3^	1.97 × 10^3^	3.94 × 10^3^
	Std	1.24 × 10^2^	2.54 × 10^2^	6.82 × 10^1^	1.60 × 10^2^	4.71 × 10^2^	4.28 × 10^2^	7.10 × 10^2^	1.19 × 10^3^	1.81 × 10^2^	7.25 × 10^2^
F25	Mean	**1.05 × 10^3^**	1.95 × 10^3^	1.27 × 10^3^	1.56 × 10^3^	9.50 × 10^3^	3.93 × 10^3^	2.39 × 10^4^	2.10 × 10^4^	4.09 × 10^3^	3.26 × 10^3^
	Std	7.56 × 10^1^	2.68 × 10^2^	7.92 × 10^1^	1.78 × 10^2^	1.78 × 10^3^	4.14 × 10^3^	1.70 × 10^3^	2.63 × 10^3^	1.05 × 10^3^	1.68 × 10^3^
F26	Mean	**8.25 × 10^3^**	1.36 × 10^4^	1.38 × 10^4^	1.87 × 10^4^	3.14 × 10^4^	2.18 × 10^4^	4.85 × 10^4^	4.65 × 10^4^	1.38 × 10^4^	1.85 × 10^4^
	Std	1.02 × 10^3^	1.93 × 10^3^	6.78 × 10^2^	3.69 × 10^3^	2.49 × 10^3^	3.04 × 10^3^	2.66 × 10^3^	3.33 × 10^3^	1.67 × 10^3^	3.37 × 10^3^
F27	Mean	**9.06 × 10^2^**	1.02 × 10^3^	9.12 × 10^2^	1.07 × 10^3^	2.99 × 10^3^	1.79 × 10^3^	1.03 × 10^4^	7.84 × 10^3^	1.55 × 10^3^	1.58 × 10^3^
	Std	7.48 × 10^1^	1.02 × 10^2^	7.90 × 10^1^	1.37 × 10^2^	1.15 × 10^3^	3.26 × 10^2^	1.86 × 10^3^	1.49 × 10^3^	2.36 × 10^2^	5.52 × 10^2^
F28	Mean	**1.06 × 10^3^**	3.57 × 10^3^	1.21 × 10^3^	1.80 × 10^3^	1.36 × 10^4^	1.55 × 10^4^	2.59 × 10^4^	2.39 × 10^4^	6.50 × 10^3^	6.73 × 10^3^
	Std	2.29 × 10^2^	1.72 × 10^3^	1.28 × 10^2^	3.44 × 10^2^	2.10 × 10^3^	7.44 × 10^3^	1.55 × 10^3^	2.19 × 10^3^	1.43 × 10^3^	3.41 × 10^3^
F29	Mean	4.53 × 10^3^	5.14 × 10^3^	6.04 × 10^3^	**4.45 × 10^3^**	1.07 × 10^4^	7.94 × 10^3^	3.28 × 10^5^	1.68 × 10^5^	6.65 × 10^3^	4.93 × 10^3^
	Std	7.28 × 10^2^	5.71 × 10^2^	4.36 × 10^2^	5.33 × 10^2^	1.73 × 10^3^	2.57 × 10^3^	2.19 × 10^5^	1.69 × 10^5^	4.54 × 10^3^	7.99 × 10^2^
F30	Mean	**3.27 × 10^5^**	1.55 × 10^6^	6.77 × 10^5^	9.88 × 10^5^	4.12 × 10^9^	1.68 × 10^8^	3.57 × 10^10^	2.34 × 10^10^	1.14 × 10^9^	1.25 × 10^9^
	Std	4.50 × 10^5^	1.70 × 10^6^	3.00 × 10^5^	5.67 × 10^5^	3.69 × 10^9^	1.26 × 10^8^	6.58 × 10^9^	6.43 × 10^9^	8.16 × 10^8^	1.37 × 10^9^

Values are given to three significant figures in scientific notation. Mean, mean error over 30 runs; Std, sample standard deviation. Bold marks the lowest mean error on each function, determined from the unrounded values.
